# Interprofessional collaboration and patient-reported outcomes in inpatient care: a systematic review

**DOI:** 10.1186/s13643-022-02027-x

**Published:** 2022-08-13

**Authors:** Laura Kaiser, Susann Conrad, Edmund A. M. Neugebauer, Barbara Pietsch, Dawid Pieper

**Affiliations:** 1 Witten/Herdecke University, Witten, Germany; 2 Berlin, Germany; 3 Brandenburg Medical School Theodor Fontane, Neuruppin, Germany; 4 Ludwigsburg, Germany; 5 Institute for Research in Operative Medicine, Witten, Germany; 6 Institute for Health Services and Health System Research (IVGF), Brandenburg Medical School Theodor Fontane, Rüdersdorf, Germany; 7 Center for Health Services Research, Brandenburg Medical School Theodor Fontane (ZVF-BB), Rüdersdorf, Germany; 8 Faculty of Health Sciences Brandenburg (FGW), Potsdam, Germany

**Keywords:** Interprofessional, Interdisciplinary, Collaboration, Patient-reported outcomes, Patient-reported experiences, Quality improvement, Quality of care, Inpatient

## Abstract

**Background:**

Interprofessional collaboration (IPC) is seen as the “gold standard” of comprehensive care, but credible evidence concerning the effects on patient-reported outcomes (PRO) is lacking. The aim of this systematic review is to study the effect of IPC on PRO in inpatient care.

**Methods:**

We systematically searched six electronic databases (PubMed, Web of Science/Social Science Citation Index, CENTRAL (Cochrane Library), Current Contents (LIVIVO), CINAHL, and Embase) for studies published between 1997 and 2021. Additional studies were identified through citation tracking, manually searching the Internet and Google Scholar, and consultation of experts. Risk of bias (RoB) was assessed using the RoB 2 tool for randomized controlled trials (RCTs) and ROBINS-I for non-randomized studies (NRS). The included controlled before-and-after study (CBA) was assessed using both the ROBINS-I and the Effective Practice and Organization of Care (EPOC) quality criteria. Results were synthesized through narrative description, grouping, and thematic analysis of extracted data.

**Results:**

The search yielded 10,213 records, from which 22 studies (16 RCTs, five NRS, and one CBA) fulfilled the inclusion criteria. In all but five studies, RoB was assessed as being high (RoB 2) resp. critical or serious (ROBINS-I). Within these 22 studies, nine inductively derived outcomes were assessed: (i) quality of life, (ii) coping, (iii) functional ability and health status, (iv) psychiatric morbidity, (v) pain, (vi) managing one’s own health care, (vii) treatment success, (viii) satisfaction, and (ix) therapeutic relationship. While some studies do not report effect estimates, and some of the reported effects appear to be imprecisely estimated, the overall results indicate that IPC may affect PRO positively across all outcomes.

**Conclusions:**

Due to high clinical heterogeneity and high RoB, the question whether IPC affects PRO cannot be answered conclusively. Methodically rigorous studies are needed in order to answer the question of effectiveness of IPC.

**Systematic review registration:**

PROSPERO CRD42017073900

**Supplementary Information:**

The online version contains supplementary material available at 10.1186/s13643-022-02027-x.

## Background

Interprofessional collaboration (IPC) has become the key approach to comprehensive care [1], especially in the treatment of multimorbid patients or illnesses that require the involvement of different professions. As “involvement” does not necessarily mean “collaboration,” there is a steadily growing number of projects searching for the “best practice” of IPC [2]. Not only researchers but also clinicians, healthcare professionals, and policy makers are interested in IPC as it seems to be very promising not only for the quality of care but also for economic reasons.

When examining the impact of a complex intervention like IPC, it is one approach to focus on patient-reported outcomes (PRO). PRO are an essential part of treatment evaluation and “of most importance to patients and families” [3]. The inclusion of patients’ perspectives may lead to a better understanding of patient-centered care, treatment quality, and patients’ treatment decisions and can be relevant to provide the best possible health care for this reason. However, most of the studies still concentrate on objective outcomes, such as mortality, rehospitalizations, length of stay, and healthcare costs.

Pannick et al. [4] reviewed the literature with regard to length of stay, readmission, or mortality rates for general medical wards and found only small effects of IPC. Although they consider PRO to be “valuable,” the effect of IPC on PRO has not been studied. The Cochrane review by Reeves et al. (2017) [1] aimed to assess the impact of IPC on, among other objective outcomes, quality of life, and patient-assessed quality of care. Only one of nine included randomized controlled trials (RCTs) focused on patient-reported quality of care, but due to a very low certainty of evidence, the authors concluded to be “uncertain” whether IPC improves PRO. Considering the steadily growing role of IPC in the health sector, it is therefore important to constantly (re-)evaluate its impact on PRO, and studies are needed which review the current state of literature on this topic.

As the inpatient setting represents a place in which different healthcare professions work next to each other, it is probably easier to implement and evaluate interprofessional interventions in inpatient than in outpatient care. Moreover, compared to ambulatory setting, inpatient care operates in a more controlled setting. For this reason, this systematic review focuses on the question whether IPC affects PRO in inpatient care and, if so, whether there are any heterogenous effects of IPC within different medical fields and/or study population or by type of intervention.

The review follows the Preferred Reporting Items for Systematic Reviews and Meta-Analyses PRISMA [5] (Additional file 1). This systematic review is registered in PROSPERO (registration number CRD42017073900).

## Methods

The full study protocol [2] has been published after undergoing peer review. There are three modifications — all of them methodical extensions — in comparison with the study protocol:

- We extended the exclusion criteria in accordance with the PICO scheme (addition of “population not suitable,” “study design not suitable,” and “methodical limitations”).
- Moreover, the risk of bias of included controlled before-and-after studies (CBAs) is evaluated using the quality criteria of the Cochrane Effective Practice and Organization of Care Groups (EPOC) [6] and the “Risk Of Bias In Non-randomized Studies” of Interventions tool (ROBINS-I) [7], as well.
- The extraction includes more variables than previously planned: indication, number of professions involved in the intervention, average treatment intensity (in hours), average length of stay of the intervention group (in days), statistical balance at baseline (y/n), and outcome measure.

### Literature search

We developed the search strategies (see Additional file 2) in collaboration with an information specialist as well as a researcher with long-term experience in conducting systematic reviews and developing search strategies. The Peer Review of Electronic Search Strategies (PRESS) Checklist [8] was used to develop the search strategies.

We searched the electronic databases PubMed, Web of Science/Social Science Citation Index (SSCI), CENTRAL (Cochrane Library), Current Contents (LIVIVO), CINAHL (EBSCO), and Embase for records published between 1997 and April 2021. The first literature search was carried out in July 2017 and was updated in July 2019. A third update was performed in April 2021 using the two databases which revealed the most records in the previous searches (SSCI and E). Additional studies were identified through forward and backward citation tracking, manual search of Google Scholar (using the keywords “interprofessional collaboration” alone or in combination with “impact,” “effect,” or “patient-reported outcomes”), and consultation of authors when full texts were not available.

### Inclusion criteria (PICO)

Our inclusion criteria are based on the PICO scheme.

#### Types of participants

The review only includes study populations of patients who received interprofessional interventions in an inpatient setting.

#### Types of interventions

The inclusion of interventions is based on a pre-specified definition of IPC. Specifically, IPC is defined as a work-sharing cooperation in which professionals from more than one health or social care profession cooperate with the explicit goal of improving the healthcare quality. This definition is adapted from two key publications, the Cochrane review by Reeves et al. [1] and the systematic review by Pannick et al. [4]. According to Reeves et al. [9], interprofessional interventions can be classified into three different types. The interventions described in the included studies are assigned to these individual types of intervention:

1. “Interprofessional education defined as interventions that included a curriculum with explicitly stated learning objectives/outcomes and learning activities (e.g., seminars and simulation) aimed at improving collaboration”
2. Interprofessional practice defined as interventions that aimed to improve how professionals interacted in practice through the use of activities such as meetings or checklists
3. “Interprofessional organization defined as interventions that aimed to promote collaboration by the use of institutional policies, clinical guidelines or the redesign of workspaces” [9]

#### Types of included studies

As we have learned from studies published at the time of the initial literature search (e.g., the Cochrane Review by Reeves et al. 2017 [1]), PRO of IPC interventions are rarely investigated within randomized controlled studies (see Lidstone et al. 2020 [10] as current evidence of this). Thus, to ensure that all available evidence is reviewed on this question, we decided to include not only RCTs but also non-randomized studies (NRS), CBAs, and interrupted time series (ITS) in this review and to present our results, both in the manuscript and in the tables, with regard to study designs. The EPOC criteria and terminology [11] are used to define the different study types.

#### Types of outcomes

Studies focusing on PRO, such as overall satisfaction, willingness to recommend, quality of life, or self-reported success of treatment are included in this review, regardless of whether the outcome is defined as primary or secondary.

### Exclusion criteria

There were eleven reasons for the exclusion of studies (see Table 1). Studies were excluded if they did not meet the criteria of the PICO scheme, if they were duplicates, animal studies, written in a language other than English or German, or if the full text was not available.

**Table 1:** Exclusion criteria

Exclusion criteria	
**A1**	Thematically not relevant (research question not suitable, no interprofessional collaboration as defined, no patient-reported outcomes)
**A2**	Population not suitable (outpatient)
**A3**	Study design not suitable
**A4**	Methodical limitations (e.g., partially or completely missing results, study does not report on an effect resulting from the intervention)
**A5**	Form of publication (e.g., Comment, Letter to the Editor)
**A6**	Duplicate
**A7**	Context not transferable (WHO mortality stratum B to E)
**A8**	Date of publication (date before 1997)
**A9**	Language (not in German or English)
**A10**	Full text not available
**A11**	Animal study

As a result of the decisions of the Bologna Conference in 1999, many changes have taken place in the vocational education and professional position of healthcare providers in many countries. Due to these changes, both responsibilities and awareness concerning “collaboration” have shifted. To allow a general comparability between studies of different countries, we decided to limit the search period to the previous 20 years relative to the initial date the literature search was carried out in 2017. For this reason, the final search period covers years from 1997 to April 2021 (i.e., 24 years). Additionally, we restricted the countries to those which belong to the World Health Organizations’ (WHO) mortality strata A [12] for external validity reasons.

### Selection of studies

The selection of studies occurred in a two-stage screening process, where the first screening focused on title and abstracts and the second screening on full texts. Two reviewers (LK and SB) screened a random subsample of 10% of the full sample of studies independently. Since the inter-rater reliability within this subset of studies was sufficiently high (kappa statistic of 0.84), subsequent screening of the remaining 90% of the sample was conducted with each screener covering 50% of the remaining sample. In update literature searches, LK carried out the first screening. Next, the full texts of all included studies have been screened independently by LK and SC and were either included or excluded according to the defined criteria (second screening). In case of any disagreements or uncertainties during the screening process, studies were discussed regarding their eligibility.

### Quality assessment

RoB was assessed by LK using the Cochrane “risk of bias 2” (RoB 2) tool [13] for RCTs as well as the ROBINS-I tool [7] for NRS and CBAs. Moreover, the EPOCs’ quality criteria [6] was used to assess the methodological quality of the included CBA.

### Data extraction

All studies included in the second screening were subject to data extraction. LK extracted data on country, setting (medical field), indication, definition of IPC, description of intervention and the authors’ suggested causal mechanism, details to control conditions, number of professions involved (intervention), treatment intensity (hours, mean), length of stay (intervention group, mean, days), study design, outcome measure, study population size, participant demographics, intervention classification to one of the three intervention groups (interprofessional education, interprofessional practice, interprofessional organization), times of measurement, outcomes (such as overall satisfaction, willingness to recommend, quality of life or self-reported success of treatment), baseline imbalances, and statistical data for calculation of effect sizes and/or reported effect sizes.

### Data synthesis

As there is high clinical heterogeneity in the included studies and only one study with low risk of bias, the authors decided to waive the originally planned quantitative meta-analysis. Therefore, results are presented for each outcome concept using narrative synthesis of effect estimates (unstandardized mean differences (MD), standardized effect estimates (Cohens’ d, Hedges’ g), and/or *p*-values) as they are reported in the included studies.

## Results

### Search results

**Fig. 1:**
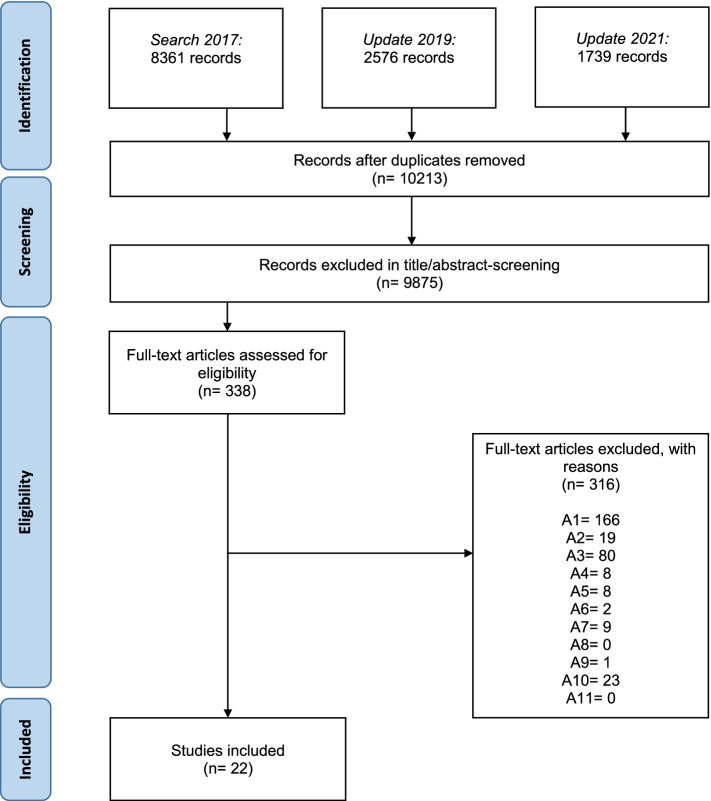
Flow diagram

The systematic searches yielded 10,213 records (see Fig. 1). After the first screening, there were 338 records eligible for the second screening. Twenty-two studies (16 RCTs [14–29], five NRS [30–34], and one CBA [35]) were included as a result of the second screening and subject to data extraction. Studies excluded in the second screening can be found in the supplementary appendix (see Additional file 3).

### Study characteristics

Studies include between 20 [19] and 1531 [15] patients and are conducted in Australia [19, 28], Denmark [14, 34], France [31], GB [17, 22], Germany [21, 23, 27, 29, 32, 33], Italy [24], Netherlands [35], Norway [18], Switzerland [30], and the USA [15, 16, 20, 25, 26]. Five studies focus on patients with chronic pain [21, 23, 30, 32, 33], four studies on patients undergoing palliative care [16, 19, 20, 26], and two studies on patients with neurological diseases (Parkinson’s disease (PD) [24], multiple sclerosis [14]), cancer [27, 31], or severe mental illness [34, 35]. Cognitive impairment [17] in old age, fibromyalgia [18], general medical patients [25], patients with anorexia nervosa [29], critical care survivors [28], homeless patients [22], and patients in old age [15] are study subject in one study each. Based on the categorization by Reeves et al. [9] (see above), nine studies describe an intervention that can be categorized as “interprofessional practice” intervention [15, 16, 19, 22, 25, 26, 28–30], and one study [24] evaluates an “interprofessional organization” intervention. The remaining studies assess the effect of interventions containing elements of at least two [14, 18, 20, 23, 27, 31–33, 35] or even all three types of interventions [17, 21, 34]. The number of professions involved varies from two [22, 25] to 10 [14]. Only one study (Gade et al. 2008 [16]) provides tentative evidence of a suggested causal mechanism. No information concerning the number of professions could have been extracted from four studies [23, 27, 30, 32]. The observational period between baseline t0 and follow-up t1 ranges from 2 or 3 days [16] (control resp. intervention group) to 26 [14] weeks. The study characteristics, including indications, details of intervention and control, number of patients, time points, and both outcomes and outcome measures, can be found in Table 2; the complete extraction sheet is in Additional file 4.

**Table 2:** Characteristics of included studies

Source (country)	Indication	Intervention				Control	***N*** patients (assign.)	Follow-up(s) (weeks since T0)	Outcome (measure)
		Description	IPC type	No. of prof.	Treatment intensity (hours, mean)				
**RCT**									
Boesen et al. 2018 [14] (Denmark)	Multiple sclerosis	4 weeks of continuous hospitalization with 20 days of scheduled rehabilitation	IP + IO	10	70	No treatment (wait-list)	427	26	QoL — generic (EQ-5D-5L; EQ-VAS; 15-D questionnaire) QoL — disease-specific (FAMS, MSIS-29: physical, psychological)
Cheung et al. 2010 [19] (Australia)	Palliative care (preterminal or terminal condition)	Usual care + consultation from a palliative care team (intensive care unit)	IP	4	N/A	Usual care	20	0.7 (IG) resp. 0.4 (CG)	Satisfaction (unknown outcome measure/self-developed)
Counsell et al. 2000 [15] (USA)	Old age (> 70)	Acute Care for Elders (ACE): adaption of environment + patient-centered care (e.g., nursing care plans) + discharge planning + review of medical care + daily interdisciplinary team rounds	IP	4	N/A	Usual care	1531	6	Satisfaction (unknown outcome measure/self-developed) Functional ability and health status — generic (ADL decline: generic; IADL)
Gade et al. 2008 [16] (USA)	Palliative care	Consultative interdisciplinary palliative care service (IPCS)	IP	4	N/A	Usual care	517	0.42 (IG) resp. 0.28 (CG)	QoL — generic (MCOHPQ: physical area, emotional/relationship area, spiritual area, quality of life) Satisfaction — generic (MCOHPQ: place of care environment scale, doctors, nurses/other healthcare providers communication scale)
Goldberg et al. 2013 [17] (GB)	Cognitive impairment in old age (> 65)	Acute geriatric medical ward with specialist mental health staff + staff training and education + therapeutic program + adaption of environment + inclusive approach to family carers	IP + IE + IO	7	N/A	Usual care	600	13	QoL — generic (EQ-5D: self-completed, proxy completed) QoL — disease-specific (DEMQOL: self-completed, proxy completed) Functional ability and health status — generic (LHS)
Grudzen et al. 2016 [20] (USA)	Palliative care for patients with advanced cancer	Palliative care consultation: symptom assessment and treatment + goals of care + advance care plan + transition planning	IP+ IO	4	N/A	Usual care	136	6, 12	QoL — disease-specific (FACT-G) Psychiatric morbidity (PHQ-9)
Hamnes et al. 2012 [18] (Norway)	Fibromyalgia	Self-management program: individual consultations + individual and group exercises + writing goals + group discussion + patient education + walking + presentation of organizations + creating activity + visiting museum	IP + IO	8	N/A	No treatment (wait-list)	150	3	Psychiatric morbidity (GHQ-20) Coping — disease-specific (ASES: pain, symptoms, function) Functional ability and health status — disease-specific (fibromyalgia impact questionnaire) Managing one’s own health care (EC-17)
Hechler et al. 2014 [21] (Germany)	Chronic pain (pediatric)	Intensive interdisciplinary pain treatment (IIPT): education and goal determination + acquisition of pain coping strategies + treatment of emotional distress + family therapy and weekly family sessions + optional therapy-related drug treatment + physiotherapy + relapse prevention	IP + IE + IO	7	137	No treatment (wait-list)	120	3.5	Pain — generic (faces pain scale — revised) Psychiatric morbidity (DIKJ: depression) Coping — generic (PRCQ-C: catastrophizing) Functional ability and health status — generic (P-PDI: pediatric; partly proxy completed)
Hewett et al. 2016 [22] (GB)	Homelessness	General practitioner ward rounds + nurse practitioner patient support + weekly multiagency meeting	IP	2	N/A	Usual care	414	6	QoL — generic (EQ-5D-5L)
Mangels et al. 2009 [23] (Germany)	Chronic low back pain	Behavioral-medical rehabilitation treatment: usual care + psychologic treatment elements (manualized group education/training for psychologic pain management, progressive muscle relaxation, individual sessions with psychotherapist)	IP + IE	N/A	N/A	Usual care	244	4 (IG) resp. 3.5 (CG), 57 (IG) resp. 55 (CG)	QoL — generic (LSQ-G; SF-12: physical health status, mental health status) Psychiatric morbidity (BDI) Coping — generic (FESV: action-oriented coping, subjective coping competence, cognitive restructuring, counter activities, mental distraction, relaxation) Functional ability and health status — generic (PDI, adults; PSEQ; SES, affective pain perception, sensory pain perception)
Monticone et al.2015 [24] (Italy)	Parkinson’s disease	Multidisciplinary rehabilitative care: motor training + cognitive training + ergonomic education	IO	4	N/A	General physiotherapy	70	8, 52 (except GPE)	QoL — disease-specific (I-PDQ-39: mobility, activities of daily living, emotional well-being, stigma, social support, cognition, communication, bodily discomfort) Functional ability and health status — disease-specific (MDS-UPDRS: part 3) Treatment success (GPE)
O'Leary et al. 2016 [25] (USA)	General medical patients	Daily patient-centered bedside rounds with communication tool as a framework for discussion	IP	2	N/A	Usual care	650	During treatment: Picker and PAM-SF: 0 Post-discharge: Press Ganey and HCAHPS: 0.2–6	Satisfaction — generic (Picker: do not say different things, do not give conflicting information, do not talk in front of you as if you were not there, involved in decisions, worked as a team, overall satisfaction; Press Ganey: work as a team (how often), staff effort; HCAHPS: overall rating hospital, likelihood to recommend) Therapeutic relationship (PAM-SF)
Sidebottom et al. 2015 [26] (USA)	Palliative care for patients with acute heart failure	Usual care + PCT consultation + assessment of symptom burden, depression and QoL + emotional, spiritual, and psychosocial aspects of care + coordination of care orders + recommendations for current or future treatment	IP	4	N/A	Usual care	232	4, 13	QoL — disease-specific (MLHF) Pain — generic (ESAS) Psychiatric morbidity (PHQ-9)
Singer et al. 2019 [27] (Germany)	Cancer patients with high distress level (HADS score >= 13); cancer patients with moderate or low distress level (HADS score < 13)	Stepped care: structured psychosocial care — integration of distress screening as a regular topic in doctor-patient consultation (and, if necessary, consultation of psychosocial services)	IE + IO	N/A	N/A	Usual care	1012	26	Satisfaction, generic (QPP (modified): possibility to converse with doctors and/or psychologists/social workers, shared decision-making, doctors (empathic), patient orientation)
Wu et al. 2019 [28] (Australia)	Critical care survivors	In-reach multidisciplinary rehabilitation program	IP	4	N/A	Usual care	66	Discharge or admission (no further details), 26, 52	QoL — generic (SF-12: physical health, mental health; AQoL-4D) Psychiatric morbidity (DASS-21) Functional ability and health status (Lawton’s Instrumental Activities of Daily Living Scale)
Ziser et al. 2021 [29] (Germany)	Patients with anorexia nervosa	Motivation-enhancing psychotherapy for inpatients with anorexia nervosa (MANNA) plus multidisciplinary inpatient treatment	IP	6	N/A	Usual care	22	5, 10	Psychiatric morbidity (EDE-Q) Treatment success (URICA-S: precontemplation, contemplation, action, maintenance) Therapeutic relationship (HAQ)
**NRS**									
Angst et al. 2009 [30] (Switzerland)	Chronic pain	Zurzach interdisciplinary pain program (ZISP): medical care including adapted drug therapy + activity exercises + psychotherapy + interdisciplinary meetings	IP	N/A	> 100	Usual care	331	4 (IG) resp.3 (CG); 26	QoL — generic (SF-36: physical functioning, social functioning) Pain – generic (WHYMPI: pain severity, life control) Psychiatric morbidity (HADS: depression, anxiety) Coping — generic (CSQ: catastrophizing, ability to decrease pain)
Brédart et al. 2009 [31] (France)	Cancer	Use of a complex health care needs screening tool + weekly multidisciplinary liaison staff meeting + adoption of clinical guidelines + five mobile teams + professionals as consultants in the various hospital ward + supervision by a physician + assistance by paramedical professional	IP + IO	6	N/A	Usual care	216	9	QoL — disease-specific (EORTC QLQ-C30: physical functioning, role functioning, emotional functioning, social functioning, overall health status) Satisfaction — disease-specific (EORTC IN-PATSAT32: doctors: technical competence, interpersonal quality, information, availability; nurses/paramedical personnel: technical competence, interpersonal quality, information, availability, general satisfaction)
Hampel et al. 2015 [32] (Germany)	Chronic low back pain and depressive symptoms	Multidisciplinary orthopedic rehabilitation program + cognitive-behavioral group training of depressive symptoms	IP + IE	N/A	86	Usual care	84	4, 26, 52, 104	Psychiatric morbidity (ADS: depressive symptoms, HADS: anxiety, SCL-90-R: somatization)
Marcussen et al. 2020 [34] (Denmark)	Severe mental illness	Interprofessional training unit: interactive workshop, small-group work, clinical care teamwork, interprofessional group tutorials for students, patient participation	IP + IE + IO	6	N/A	Usual care	552	Day of discharge (no further details)	QoL — generic (SF-36: physical functioning, mental functioning) Psychiatric morbidity (K10) Satisfaction, generic (CSQ-8)
Semrau et al. 2015 [33] (Germany)	Chronic low back pain	Interdisciplinary rehabilitation program (PASTOR): biopsychosocial health education + behavioral exercise therapy + cognitive-behavioral psychological therapy + workplace-related information + team meetings	IP + IE	9	48	Usual care	554	3 (except FFkA), 52	QoL — generic (SF-12: physical health, mental health) Pain — generic (German Pain Questionnaire) Coping — generic (FESV: action-oriented coping, subjective coping competence, cognitive restructuring, counter activities, mental distraction, relaxation; AEQ: help-/hopelessness, catastrophizing, thought suppression, anxiety/depression, positive mood, avoidance of physical activities when dealing with severe pain, humor/distraction when dealing with severe pain, pain persistence behavior when dealing with severe pain) Functional ability and health status — generic (FFkA: sport activity (hours/week), total physical activity (hours/week)) Functional ability and health status — disease-specific (FFbH-R)
**CBA**									
Deenik et al. 2018 [35] (Netherlands)	Severe mental illness	Multidisciplinary lifestyle enhancing treatment (MULTI): lifestyle interventions + improved daily structure + review of existing policies + participation of nurses in day-to-day program + supervision by a psychiatrist	IP + IO	4	N/A	Usual care	123	N/A	QoL — generic (EQ-5D; WHOQOL-BREF: physical, psychological, social, environmental)

*ADS* General Depression Scale (Allgemeine Depressions-Skala), *AQoL-4D* assessment of quality of life, *ASES*, Arthritis Self-Efficacy Scale, *Assign* assignment, *AEQ* avoidance-endurance questionnaire, *ADL* activities of daily living, *BDI* Beck Depression Inventory, *CG* control group, *CSQ* coping strategies questionnaire, *CSQ-8* client satisfaction questionnaire, *DASS-21* Depression Anxiety Stress Scale, *DEMQOL* dementia quality of life measure, *DIKJ* depression inventory for children and adolescents, *EDE-Q* Eating Disorder Examination Questionnaire, *ESAS* Edmonton System Assessment Scale, *EC-17* Effective Musculoskeletal Consumer Scale, *EORTC IN-PATSAT32* EORTC Inpatient Satisfaction with Cancer Care Questionnaire, *EORTC QLQ-C30* EORTC Quality of Life with Cancer Questionnaire, *EQ VAS* EuroQol Visual Analogue Scale, *EQ-5D (-5L)* EuroQol 5D (-5L, long version), *FACT-G* Functional Assessment of Cancer Therapy-General measure, *FAMS* functional assessment of multiple sclerosis questionnaire, *FESV* pain management questionnaire, *FIQ* fibromyalgia impact questionnaire, *FFbH-R* Hannover Functional Ability Questionnaire-back pain, *FFkA* Freiburg Questionnaire of physical activity, *GHQ-20* General Health Questionnaire, *GPE* global perceived effect, *HADS* Hospital Anxiety and Depression Scale, *HAQ* Helping Alliance Questionnaire, *HCAHPS* Hospital Consumer Assessment of Healthcare Providers and Systems, *IADL* independent activities of daily living, *IE* interprofessional education, *IG* intervention group, *IP* interprofessional practice, *I-PDQ-39* Italian 39-question Parkinson’s disease questionnaire, *IO* interprofessional organization, *K10* Kessler psychological distress scale, *LHS* London handicap scale, *LSQ-G* German Life Satisfaction Questionnaire (Fragebogen zur Lebenszufriedenheit), *MCOHPQ* Modified City of Hope QoL Patient Questionnaire, *MDS-UPDRS* Italian Movement Disorder Society Unified Parkinson’s Disease Rating Scale, *MLHF* Minnesota Living with Heart Failure Questionnaire, M*SIS-29* Multiple Sclerosis Impact Scale-29 version 2, *N/A* not available, *no. of prof* number of professions, *PAM-SF* Patient Activation Measure (Short Form), *PCT* palliative care team, *PDI* Pain Disability Index, *PHQ-9* Patient Health Questionnaire, *P-PDI* Pediatric Pain Disability Index, *PRCQ-C* pain-related cognitions questionnaire for children, *PSEQ* pain self-efficacy questionnaire, *QoL* quality of life, *QPP* quality of care from the patient’s perspective, *SCL-90-R* Symptom Checklist-90-R, *SES* Pain Perception Scale, *SF-12* Short Form 12, *SF-36* Short Form 36, *URICA-S* University of Rhode Island Change Assessment (short version), WHOOL-BREF World Health Organization Quality of Life Assessment scale, *WHYMPI* West Haven-Yale Multidimensional Pain Inventory

### Risk of bias

The results of risk of bias assessment are detailed in the Additional file 5 and summarized in Tables 3 and 4.

**Table 3:** Risk of bias in RCTs using the risk of bias 2 tool

Study	Randomization	Deviations from intended interventions	Missing data	Measurement of the outcome	Selection of reported results	Overall risk of bias
Boesen et al. 2018 [14]	++	+/-	++	--	+/-	--
Cheung et al. 2010 [19]	+/-	+/-	--	--	+/-	--
Counsell et al. 2000 [15]	++	--	++	--	+/-	--
Gade et al. 2008 [16]	+/-	+/-	++	--	+/-	--
Goldberg et al. 2013 [17]	++	--	+/-	++	+/-	--
Grudzen et al. 2016 [20]	++	--	++	--	+/-	--
Hamnes et al. 2012 [18]	++	++	++	++	+/-	+/-
Hechler et al. 2014 [21]	++	++	++	++	++	++
Hewett et al. 2016 [22]	+/-	++	--	--	+/-	--
Mangels et al. 2009 [23]	+/-	+/-	++	--	+/-	--
Monticone et al. 2015 [24]	++	++	++	++	+/-	+/-
O'Leary et al. 2016 [25]	--	--	++	--	+/-	--
Sidebottom et al. 2015 [26]	--	--	--	++	+/-	--
Singer et al. 2019 [27]	++	++	++	++	++	++
Wu et al. 2019 [28]	++	--	++	--	+/-	--
Ziser et al. 2021 [29]	+/-	++	++	--	+/-	--

++Low, +/-some concerns, --high

**Table 4:** Risk of bias in NRS and CBA using the ROBINS-I tool

Study	Study design	Confounding	Selection of participants	Classification of interventions	Deviations from intended interventions	Missing data	Measurement of the outcome	Selection of reported results	Overall RoB
Angst et al. 2009 [30]	NRS	+	++	++	++	-	-	+	-
Brédart et al. 2009 [31]	NRS	+	++	++	++	-	-	+	-
Hampel et al. 2015 [32]	NRS	-	++	++	++	-	-	--	--
Marcussen et al. 2020 [34]	NRS	-	++	++	++	-	-	+	-
Semrau et al. 2015 [33]	NRS	+	++	++	++	++	++	++	+
Deenik et al. 2018 [35]	CBA	+	++	++	++	++	-	++	-

++Low, +moderate, -serious, --critical; *NRS* non-randomized studies, *CBA* controlled before-and-after studies, *RoB* risk of bias

### RCTs

Only two studies [21, 27] are considered to have a “low,” while twelve studies [14–17, 19, 20, 22, 23, 25, 26, 28, 29] had a “high” RoB. Two RCTs [18, 24] were rated to have “some concerns” (see Table 3).

### NRS and CBA

There is no NRS for which the RoB was rated as “low” but instead rated as “moderate” [33], “serious,” [30, 31, 34] or “critical” [32] (see Table 4). Likewise, the CBA by Deenik et al. [35] is classified as having “serious” RoB. Three EPOC criteria are rated as “not done,” two as “done,” and one as “not clear.”

### Relationship between IPC and PRO

Whether IPC affects PRO is evaluated using 59 outcome measures (see Table 5). As not all outcome measures are publicly available and/or questions explicitly presented within the respective manuscripts, an overall statement regarding the questionnaire scaling is not possible. However, the information whether full or partial questionnaires were used and whether validation studies were quoted can be found in the Additional file 6. The following nine outcomes were defined inductively during extraction process (see Table 6): QoL (quality of Life), coping, satisfaction, functional ability and health status, pain, psychiatric morbidity, managing one’s own health care, therapeutic relationship, and treatment success.

**Table 5:** Outcome measures and concepts

**QoL (source)**	***Generic*** 15-D questionnaire (Boesen et al. [14]) AQoL-4D (Wu et al. [28]) EQ VAS (Boesen et al. [14]) EQ-5D (Deenik et al. [35], Goldberg et al. [17]) EQ-5D-5L (Boesen et al. [14], Hewett et al. [22]) German Life Satisfaction Questionnaire (Mangels et al. [23]); MCOHPQ (Gade et al. [16]) SF-12 (Semrau et al. [33], Mangels et al. [23], Wu et al. [28]) SF-36 (Angst et al. [30], Marcussen et al. [34]) WHOQoL bref (Deenik et al. [35])	***Disease-specific*** DEMQOL (Goldberg et al. [17]) EORTC QLQ-C30 (Brédart et al. [31]) FACT-G (Grudzen et al. [20]) FAMS (Boesen et al. [14]) I-PDQ-39 (Monticone et al. [24]) MLHF (Monticone et al. [24]) MSIS-29 (Boesen et al. [14])
**Coping (source)**	***Generic*** AEQ (Semrau et al. [33]) CSQ (Angst et al. [30]) FESV (Semrau et al. [33]; Mangels et al. [23]) PRCQ-C (Hechler et al. [21])	***Disease-specific*** ASES (Hamnes et al. [18])
**Satisfaction (source)**	***Generic*** CSQ-8 (Marcussen et al. [34]) HCAHPS (O'Leary et al. [25]) MCOHPQ (Gade et al. [16]) Picker (O'Leary et al. [25]) Press Ganey (O'Leary et al. [25]) QPP (Singer et al. [27]) Unknown measure/self-developed (Counsell et al. [15]; Cheung et al. [19])	***Disease-specific*** EORTC IN-PATSAT 32 (Brédart et al. [31])
**Functional ability and health status (source)**	***Generic*** ADL (Counsell et al. [15]) FFkA (Semrau et al. [33]) Lawton’s Instrumental Activities of Daily Living Scale (Wu et al. [28]) LHS (Goldberg et al. [17]) PDI (Mangels et al. [23]) P-PDI (Hechler et al. [21]) PSEQ (Mangels et al. [23]) SES (Mangels et al. [23])	***Disease-specific*** FFbH-R (Semrau et al. [33]) FIQ (Hamnes et al. [18]) MDS-UPDRS (Monticone et al. [24])
**Psychiatric morbidity (source)**	***Generic*** ADS (Hampel et al. [32]) BDI (Mangels et al. [23]) DASS-21 (Wu et al. [28]) DIKJ (Hechler et al. [21]) GHQ-20 (Hamnes et al. [18]) HADS (Angst et al. [30], Hampel et al. [32]) K10 (Marcussen et al. [34]) PHQ-9 (Grudzen et al. [20]; Sidebottom et al. [26]) SCL-90-R (Hampel et al. [32])	***Disease-specific*** EDE-Q (Ziser et al. [29])
**Pain (source)**	ESAS (Sidebottom et al. [26]) German Pain Questionnaire (Semrau et al. [33]) Faces Pain Scale – Revised (Hechler et al. [21]) WHYMPI (Angst et al. [30])	
**Managing one’s own health care (source)**	EC-17 (Hamnes et al. [18])	
**Treatment success (Source)**	GPE (Monticone et al. [24]) URICA-S (Ziser et al. [29])	
**Therapeutic relationship (source)**	HAQ (Ziser et al. [29]) PAM-SF (O'Leary et al. [25])	

*ADS* General Depression Scale (Allgemeine Depressions-Skala); *AQoL-4D* assessment of quality of life; *ASES* Arthritis Self-Efficacy Scale; *AEQ* avoidance-endurance questionnaire; *ADL* activities of daily living; *BDI* Beck Depression Inventory; *CSQ* coping strategies questionnaire; *CSQ-8* client satisfaction questionnaire; *DASS-21* depression anxiety stress scale; *DEMQOL* dementia quality of life measure; *DIKJ* depression inventory for children and adolescents; *EDE-Q* Eating Disorder Examination Questionnaire; *ESAS* Edmonton System Assessment Scale; *EC-17* Effective Musculoskeletal Consumer Scale; *EORTC IN-PATSAT32* EORTC Inpatient Satisfaction with Cancer Care Questionnaire; *EORTC QLQ-C30* EORTC Quality of Life with Cancer Questionnaire; *EQ VAS* EuroQol visual analogue scale; *EQ-5D (-5L)* EuroQol 5D (-5L, long version); *FACT-G* Functional Assessment of Cancer Therapy-General measure; *FAMS* functional assessment of multiple sclerosis questionnaire; *FESV* pain management questionnaire; *FIQ* fibromyalgia impact questionnaire; *FFbH-R* Hannover Functional Ability Questionnaire-back pain; *FFkA* Freiburg Questionnaire of physical activity; *GHQ-20* General Health Questionnaire; *GPE* global perceived effect; *HADS* Hospital Anxiety and Depression Scale; *HAQ* Helping Alliance Questionnaire; *HCAHPS* Hospital Consumer Assessment of Healthcare Providers and Systems; *I-PDQ-39* Italian 39-question Parkinson´s disease questionnaire; *K10* Kessler psychological distress scale; *LHS* London handicap scale; *LSQ-G* German Life Satisfaction Questionnaire (Fragebogen zur Lebenszufriedenheit); *MCOHPQ* Modified City of Hope QoL Patient Questionnaire; *MDS-UPDRS* Italian Movement Disorder Society Unified Parkinson’s Disease Rating Scale; *MLHF* Minnesota Living with Heart Failure Questionnaire; *MSIS-29* Multiple Sclerosis Impact Scale-29 version 2; *PAM-SF* Patient Activation Measure (Short Form); *PDI* Pain Disability Index; *PHQ-9* Patient Health Questionnaire; *P-PDI* Pediatric Pain Disability Index; *PRCQ-C* pain-related cognitions questionnaire for children; *PSEQ* pain self-efficacy questionnaire; *QoL* quality of life; *QPP* quality of care from the patient’s perspective; *SCL-90-R* Symptom Checklist-90-R; *SES* Pain Perception Scale; *SF-12* Short Form 12; *SF-36* Short Form 36; *URICA-S* University of Rhode Island Change Assessment (short version); *WHOQOL-BREF* World Health Organization Quality of Life Assessment scale; *WHYMPI* West Haven-Yale Multidimensional Pain Inventory

**Table 6:** Outcomes and risk of bias

Outcome	Study	No. of participants	Risk of bias
**QoL**	8 RCTs [6–13] 4 NRS [14–17] 1 CBA [18]	4250	RCTs 1 (some concerns) 7 (high) NRS 1 (moderate) 3 (serious) CBA 1 (serious)
**Coping**	3 RCTs [11, 19, 20] 2 NRS [14, 17]	1399	RCTs 1 (low) 1 (some concerns) 1 (high) NRS 1 (moderate) 1 (serious)
**Satisfaction**	5 RCTs [3, 4, 7, 21, 22] 2 NRS [15, 16]	4498	RCTs 1 (low) 4 (high) NRS 2 (serious)
**Functional ability and health status**	7 RCTs [4, 8, 11–13, 19, 20] 1 NRS [17]	3335	RCTs 1 (low) 2 (some concerns) 4 (high) NRS 1 (moderate)
**Psychiatric morbidity**	7 RCTs [9, 11, 13, 19, 20, 23, 24] 3 NRS [14, 16, 25]	1937	RCTs 1 (low) 1 (some concerns) 5 (high) NRS 2 (serious) 1 (critical)
**Pain**	2 RCTs [20, 23] 2 NRS [14, 17]	1237	RCTs 1 (low) 1 (high) NRS 1 (moderate) 1 (serious)
**Managing one’s own health care**	1 RCT [19]	150	RCT 1 (some concerns)
**Treatment success**	2 RCTs [12, 24]	92	RCTs 1 (some concerns) 1 (high)
**Therapeutic relationship**	2 RCTs [21, 24]	672	RCTs 2 (high)

As we decided to waive the originally planned quantitative meta-analysis, we describe the reported effect estimates within the manuscript text and, moreover, summarize them within structured tables of results across studies. Furthermore, results are presented regarding the differences between groups that occurred between baseline (t0) and follow-up (t1). Some studies also present results on second follow-up. However, as this only applies to a small number of studies, these results can be found in the complete extraction sheet in Additional file 4. Due to differences in the amount of reported adjusted MD, standardized effect sizes (ES), or *p*-values (between groups), we decided to present the effect estimates of the two outcomes with the most reported effect estimates in tables within the main manuscript (i.e., QoL, coping). The effect estimates of the remaining outcomes are reported in Additional files 7, 8, 9, 10, 11, 12 and 13.

*QoL* has been assessed with 17 questionnaires (ten generic, seven disease-specific) applied in nine RCTs [14, 16, 17, 20, 22–24, 26, 28], four NRS [30, 31, 33, 34], and one CBA [35] (see Table 7). ES could only be extracted from the NRS by Angst et al. [30] (Short Form (SF)-36) and Semrau et al. [33] (SF-12). Here, estimated treatment effects are small and positive, but the corresponding confidence sets can neither rule out relatively small or large positive or negative effects. Seven studies [14, 17, 22, 24, 26, 33–35] report unstandardized MD, and four studies [16, 20, 23, 31] only present *p*-values. The majority of MD do not show any positive or negative effect of IPC. However, positive and statistically significant effects of IPC were reported in four studies focusing on *patients with multiple sclerosis* [14], *PD* [24], *acute heart failure in palliative care* [26], and *severe mental illness* [34].

**Table 7:** Reported adjusted unstandardized mean differences, standardized effect sizes, and *p*-values (between groups) in studies measuring QoL

Source (Study type)	Study population	Outcome measures QoL (total score)		Adjusted mean differences (95% ***CI*** or ***SE***)	Standardized effect sizes	***p***-value
Boesen et al. 2018 [14] (RCT)	Multiple sclerosis	FAMS (0–176)		1.6 (−1.4, 4.7)*		0.232
		MSIS-29	Physical (0–1001)‡	−0.6 (−3.0, 1.8)*		0.640
			Psychological (0–100)‡	−2.7 (−5.6, −0.1)*		0.046
		EQ-5D-5L (−0.624 to −1.000)		0.006 (−0.015, 0.028)*		0.596
		EQ VAS (0–100)		2.5 (−1.1, 5.9)*		0.112
		15D questionnaire (0.106–1.000)		0.017 (0.005, 0.030)*		0.008
Gade et al. 2008 [16] (RCT)	Palliative care	MCOHPQ	Physical area (0–10)‡			0.91
			Emotional/relationship area (0–10)‡			0.07
			Spiritual area (0–10)			0.55
			Quality of life (0–10)			0.78
Goldberg et al. 2013 [17] (RCT)	Cognitive impairment in old age (> 65)	EQ-5D	Self-completed (0–1)‡	0.00 (−0.09, 0.09)		0.96
			Proxy completed (0–1)‡	−0.07 (−0.15, 0.00)		0.06
		DEMQOL	Self-completed (0–108)	0.7 (−2.8, 4.1)		0.70
			Proxy completed (0–124)	−0.4 (−4.6, 3.8)		0.84
Grudzen et al. 2016 [20] (RCT)	Palliative care for patients with advanced cancer	FACT-G (0–108)				0.054
Hewett et al. 2016 [22] (RCT)	Homelessness	EQ-5D-5L (.)		0.09 (−0.03, 0.22)		0.151
Mangels et al. 2009 [23] (RCT)	Chronic low back pain	LSQ-G (7–49)				NS
		SF-12	Physical health status (.)			
			Mental health status (.)			
Monticone et al. 2015 [24] (RCT)	Parkinson’s disease	I-PDQ-39	Mobility (0–100)‡	−14.1 (3.4)*		
			Activities of daily living (0–100)‡	−19.6 (2.2)*		
			Emotional well-being (0–100)‡	−14.8 (2.9)*		
			Stigma (0–100)‡	−14.9 (3.4)*		
			Social support (0–100)‡	−10.2 (3.4)*		
			Cognition (0–100)‡	−10.4 (2.6)*		
			Communication (0–100)‡	−8.4 (4.8)*		
			Bodily discomfort (0–100)‡	−12.2 (2.8)*		
Sidebottom et al. 2015 [26] (RCT)	Palliative care for patients with acute heart failure	MLHF (0–105)		4.92 (4.61, 5.23)		0.000
Wu et al. 2019 [28] (RCT)	Critical care survivors	AQoL-4D (0–1)				
		SF-12	Physical health status (.) Mental health status (.)			
Angst et al. 2009 [30] (NRS)	Chronic pain	SF-36	Physical functioning (0–100)		0.06 (Hedges’ g)	0.361
			Social functioning (0–100)		0.32 (Hedges’ g)	0.076
Brédart et al. 2009 [31] (NRS)	Cancer	EORTC QLQ-C30				
			Physical functioning (.)			NS
			Role functioning (.)			NS
			Emotional functioning (.)			NS
			Social functioning (.)			NS
			Overall health status (.)			NS
Marcussen et al. 2020 [34] (NRS)	Severe mental illness	SF-36	Physical functioning (0–100) Mental functioning (0–100)	0.40 (−2.3, 1.24) 5.30 (2.71, 7.89)		0.6 0.001
Semrau et al. 2015 [33] (NRS)	Chronic low back pain	SF-12	Physical health status (0–100)	0.50 (−0.99, 1.99)	0.029 (Cohens’ d)	
			Mental health status (0–100)	0.62 (−1.35, 2.58)	0.027 (Cohens’ d)	
Deenik et al. 2018 [35] (CBA)	Severe mental illness	EQ-5D (0–1)		−0.02 (−0.12, 0.08)		0.736
		WHOQOL-BREF	Physical (1–10)	0.14 (−0.80, 1.09)		0.765
			Psychological (1–10)	−0.37 (-1.38, 0.63)		0.465
			Social (1–10)	0.63 (−0.47, 1.73)		0.257
			Environmental (1–10)	0.42 (−0.97, 1.80)		0.537

Estimates of adjusted mean differences, standardized effect sizes, or *p*-values refer to tests for difference in means between treatment and control groups at the time of follow-up (t1) or to the difference in change scores (t0–t1) between groups.(.) Not reported; *unadjusted, ‡inverted scale (lower score indicates greater impact); *AQoL-4D*, assessment of quality of life, *DEMQOL* dementia quality of life measure, *EORTC QLQ-30* EORTC Quality of Life with Cancer Questionnaire, *EQ-5D* (-5L) EuroQol 5D (-5L, long version), *EQ VAS* EuroQol visual analogue scale, *ES* effect size, *FACT-G* Functional Assessment of Cancer Therapy — General measure, *FAMS* functional assessment of multiple sclerosis questionnaire, *I-PDQ-39* Italian 39-question Parkinson’s disease questionnaire, *LSQ-G* German Life Satisfaction Questionnaire (Fragebogen zur Lebenszufriedenheit), *MCOHPQ* Modified City of Hope QoL Patient Questionnaire, *MLHF* Minnesota Living with Heart Failure questionnaire, *MSIS-29* Multiple Sclerosis Impact Scale-29 version 2, *NA* not applicable, *NS* not significant, no further details reported, *QoL* quality of life, *SE* standard error, *SF-12* Short Form 12, *SF-36* Short Form 36, *WHOQOL-BREF* World Health Organization Quality of Life Assessment scale

Five studies (three RCTs [18, 21, 23], two NRS [30, 33]) evaluate the effect of IPC on *coping* using five outcome measures in total (FESV (pain management questionnaire), PRCQ-C (pain-related cognitions questionnaire for children), ASES (Arthritis Self-Efficacy Scale), AEQ (avoidance-endurance questionnaire), and CSQ (coping strategies questionnaire) (see Table 8). The indications are largely comparable across study types with *CLBP* (*chronic low back pain*) and *chronic pain* (*pediatric*) in two RCTs [21, 23] and *CLBP* and *CP* (*chronic pain*) in NRS. Three studies [18, 30, 33] report positive, but partly insignificant positive effects. Hechler et al. [21] does not report adjusted MD, standardized effect sizes or *p*-values, whereas Mangels et al. [23] report significant *p*-values between groups.

**Table 8:** Reported adjusted unstandardized mean differences, standardized effect sizes, and *p*-values (between groups) in studies measuring coping between baseline (t0) and follow-up (t1)

Source (study type)	Study population	Outcome measures coping (total score)		Adjusted mean differences (95% ***CI*** or ***SE***)	Standardized effect sizes	***p***-value
Hamnes et al. 2012 [18] (RCT)	Fibromyalgia	ASES	Pain (10–100)	−1.83 (−6.0, 2.3)	0.12 (Cohens’ d)	0.387
			Symptoms (10–100)	2.63 (−1.3, 6.6)	0.20 (Cohens’ d)	0.189
			Function (10–100)	1.02 (−2.4, 4.4)	0.06 (Cohens’ d)	0.556
Hechler et al. 2014 [21] (RCT)	Chronic pain (pediatric)	PRCQ-C	Catastrophizing (0–2)‡			
Mangels et al. 2009 [23] (RCT)	Chronic low back pain	FESV	Action-oriented coping (.)			< 0.001
			Subjective coping competence (.)			
			Cognitive restructuring (.)			< 0.01
			Counter activities (.)			
			Mental distraction (.)			< 0.01
			Relaxation (.)			< 0.001
Angst et al. 2009 [30] (NRS)	Chronic pain	CSQ	Catastrophizing (0–100)		0.07 (Hedges’ g)	0.169
			Ability to decrease pain (0–100)		0.13 (Hedges’ g)	0.148
Semrau et al. 2015 [33] (NRS)	Chronic low back pain	FESV	Action-oriented coping (1–6)	2.36 (1.50, 3.22)	0.232 (Cohens’ d)	
			Subjective coping competence (1–6)	1.60 (0.91, 2.29)	0.197 (Cohens’ d)	
			Cognitive restructuring (1–6)	2.47 (1.68, 3.26)	0.265 (Cohens’ d)	
			Counter activities (1–6)	2.21 (1.51, 2.94)	0.263 (Cohens’ d)	
			Mental distraction (1–6)	1.80 (1.0, 2.61)	0.190 (Cohens’ d)	
			Relaxation (1–6)	2.09 (1.25, 2.92)	0.213 (Cohens’ d)	
		AEQ	Help/hopelessness (0–6)‡	−0.29 (−0.45, −0.13)	−0.158 (Cohens’ d)	
			Catastrophizing (0–6)‡	−0.12 (−0.27, 0.05)	−0.065 (Cohens’ d)	
			Thought suppression (0–6)‡	−0.09 (−0.31, 0.14)	−0.032 (Cohens’ d)	
			Anxiety/depression (0–6)‡	−0.25 (−0.44, −0.06)	−0.114 (Cohens’ d)	
			Positive mood (0–6)	0.16 (−0.04, 0.36)	0.067 (Cohens’ d)	
			Avoidance of physical activities (0–6)‡	−0.32 (−0.50, −0.14)	−0.150 (Cohens’ d)	
			Avoidance of social activities (0–6)‡	−0.35 (−0.54, −0.16)	−0.157 (Cohens’ d)	
			Humor/distraction (0–6)	0.27 (0.09, 0.46)	0.125 (Cohens’ d)	
			Pain persistence behavior (0–6)	0.09 (−0.06, 0.24)	0.052 (Cohens’ d)	

Estimates of adjusted mean differences, standardized effect sizes, or *p*-values refer to tests for difference in means between treatment and control groups at the time of follow-up (t1) or to the difference in change scores (t0–t1) between groups. (.) Not reported; ‡inverted scale (lower score indicate greater impact; *AEQ*, avoidance-endurance questionnaire; *ASES*, Arthritis Self-Efficacy Scale; *CSQ*, coping strategies questionnaire; *FESV*, pain management questionnaire; *PRCQ-C*, pain-related cognitions questionnaire for children

Five RCTs [15, 16, 19, 25, 27] as well as two NRS [31, 34] assessed *satisfaction* using two unknown outcome measures, one (EORTC IN-PATSAT32, EORTC Inpatient Satisfaction with Cancer Care Questionnaire) disease-specific and six generic (CSQ-8 (client satisfaction questionnaire), MCOHPQ (Modified City of Hope Quality of Life Patient Questionnaire), Picker Questionnaire, Press Ganey, HCAHPS (Hospital Consumer Assessment of Healthcare Providers and Systems), and QPP (quality of care from the patients’ perspective) questionnaires. None of these studies report standardized ES. Singer et al. state statistically insignificant odds ratios, whereas O’Leary et al. [25] and Marcussen et al. [34] report positive and partly statistically significant adjusted MD when asking *general medical patients* resp. *patients with severe mental illness* (see Additional file 7). Gade et al. [16] (*old age* (*> 70 years old*)) as well as Counsell et al. [15] (*patients with palliative care*) report significant differences between groups at t1 (i.e., post treatment). However, no further effect estimates were reported in these studies, and nonsignificant *p*-values are reported in Cheung et al. [19] (*patients with preterminal or terminal condition in palliative care*) as well as Brédart et al. [31] (*patients with cancer*).

*Functional ability* has been assessed in seven RCTs [15, 17, 18, 21, 23, 24, 28] and one CT [33] using 12 different kinds of outcome measures (see Additional file 8). Three studies [21, 23, 28] do not report any MD, ES, or *p*-value but raw mean scores and standard deviations. One study only [15] provides information about *p*-values but no ES estimates. Statistically positive, but insignificant effects were reported in Goldberg et al. [17] (MD 0.5 (95% *CI*: −5.2, 6.2, *p* = 0.87; *patients with cognitive impairment in old age*) as well as Hamnes et al. [18] (*ES* = 0.15, *p* = 0.265; Cohens’ d); *patients with fibromyalgia*). Semrau et al. [33] provide impact estimates of IPC on patients with *CLBP*. Adjusted MD and ES are estimated to be positive (i.e., in favor of the experimental group), but only one estimate is significantly different from zero (FFbH-R (Hannover Functional Ability Questionnaire-back pain) *MD* 0.91 (95% *CI*: −1.43, 3.24); FFkA (Freiburg Questionnaire of physical activity), *MD* 0.63 (95% *CI* 0.12, 1.13)). Moreover, Monticone et al. [24] provide evidence of a statistically significant reduction in MDS-UPDRS (part 3) (Italian Movement Disorder Society Unified Parkinson’s Disease Rating Scale) (*MD* 24.5 (SE 3.2); inverted scale), i.e., a desirable effect favoring the treated group (*patients with PD*).

*Pain* was assessed using the ESAS (Edmonton System Assessment Scale) and the FPS-R (Faces Pain Scale–Revised) in RCTs [21, 26] and the WHYMPI (West Haven-Yale Multidimensional Pain Inventory) and German Pain Questionnaire in NRS [30, 33] (see Additional file 9). One study [21] neither reports on MD nor standardized ES or *p*-values. Semrau et al. [33] describe a positive, but statistically insignificant effect on pain after questioning *patients with CLBP* using the German Pain Questionnaire (*ES* = −0.013, *p* = 0.755, Cohens’ d; inverted scale). Positive and statistically significant effects have been reported in Sidebottom et al. [26] (*MD* 3.69 (95% *CI*: 3.39, 3.99), *p* = 0.000); *patients within palliative care and with acute heart failure*) as well as Angst et al. [30] (*ES* = 0.09, *p* = 0.034 resp. 0.18, *p* = 0.559, Hedges’ g; *patients with CP*).

The PHQ-9 (Patient Health Questionnaire), BDI (Beck Depression Inventory), GHQ-20 (General Health Questionnaire), DASS-21 (Depression Anxiety Stress Scale), EDE-Q, and DIKJ (Depression Inventory for Children and Adolescents) were used for evaluating *psychiatric morbidity* in seven RCTs [18, 20, 21, 23, 26, 28, 29]. Moreover, the HADS (Hospital Anxiety and Depression Scale), ADS (General Depression Scale, “Allgemeine Depressions-Skala”), K10 (Kessler Psychological Distress Scale), and SCL-90-R (Symptom Checklist-90-R) were used in three CTs [30, 32, 34]. Overall, significant unstandardized MD are presented in Sidebottom et al. [26] (*MD* = 1.42 (1.12, 1.73; *p* = 0.000); *patients with acute heart failure within palliative care*) as well as Mangels et al. [23] who focused on *patients with CLBP* and report a significant between group difference at t1 (*p* < 0.01). The remaining studies do either report no [21, 28, 29, 32] or statistically insignificant estimates [18, 20, 30, 34] of treatment effects (see Additional file 10).

*Managing one’s own health care* was assessed in the RCT by Hamnes et al. [18] by using the EC-17 (Effective Musculoskeletal Consumer Scale) which revealed a positive effect in the questioning of *patients with fibromyalgia* (*ES* = 0.24, Cohens’ d; *MD* = 4.26 (95% *CI*: 0.8, 7.7) (see Additional file 11).

In contrast, in the RCT by O’Leary et al. [25], a positive, but statistically insignificant effect of IPC on *general medical patients* was found regarding the assessment of *therapeutic relationship* by using the PAM-SF (Patient Activation Measure – Short Form) (*MD* = 0.69, (95% *CI*: −2.82, 4.19); *p* = 0.58) (see Additional file 12).

*Treatment success* in *patients with PD* was evaluated in the RCTs by Monticone et al. [24] and Ziser et al. [29]. Neither MD nor standardized ES are reported in these studies. However, the between-group *p*-value revealed a significant difference between IG and CG at an 8-week follow-up in Monticone et al. [24] (*p* < 0.001; see Additional file 13).

Due to highly heterogenous (or unobserved) intervention characteristics, medical fields, and/or study populations, no further conclusions can be drawn regarding varying effects by these aspects.

## Discussion

The aim of this systematic review was to study whether IPC affects PRO in inpatient care and, if so, whether these effects vary by type of intervention, indication, and/or study population. In order to answer these questions, we systematically searched six electronic databases, and Google Scholar, tracked citations of included studies, and contacted relevant authors. The search yielded 10,213 records, from which 22 studies fulfilled the inclusion criteria in a two-step screening process. Most of the included RCTs are considered to have a high RoB [14–17, 19, 20, 22, 23, 25, 26, 28, 29]. Likewise, the RoB of NRS and CBA is mostly rated as serious [30, 31, 34, 35]. Only two studies [21, 27] have a low RoB. To summarize, while some studies do not report effect estimates, and some of the reported effects appear to be imprecisely estimated, the overall results indicate that IPC may affect PRO positively across all outcomes. Nevertheless, there are also some studies that do not report any effect. Moreover, due to heterogeneity, neither the RoBs nor the type of intervention, medical field, or study population allow further conclusions on heterogenous impacts of IPC on PRO.

To our knowledge, this is the first systematic attempt to evaluate the effectiveness of IPC on PRO including RCTs, NRS, and CBAs as well as all three types of IPC interventions and a multitude of indications. In using a purposely broad search strategy and inclusion criteria, we explicitly attempted to investigate which outcomes and indications have already been studied to contribute to an overall overview to the current state of literature. Due to the broadness of the research question, the systematic search strategy was very sensitive and yielded a lot of results. It is therefore surprising that there were only 22 studies that were included in this review. In accordance with Pannick et al. [4], we were also unable to show a clear effect of IPC on PRO. The Cochrane review by Reeves et al. [1] aimed to assess the impact of “interprofessional practice” interventions on both objective and PRO, as well as clinical process and efficiency outcomes. They also concluded that the heterogeneity of studies does not allow for a meta-analysis and a clear conclusion on the effect of IPC interventions.

While screening the literature, it became obvious that there seems to be a lack of a clear and generally valid definition of IPC. There were a lot of different synonyms used to define IPC interventions, such as interdisciplinary [15, 16, 21, 25, 30, 33] or interprofessional [34], multidisciplinary [14, 18, 23, 24, 27–29, 31, 32, 35] or comprehensive [20]/enhanced [17]/intensive [19]/complex [22]/integrating [26] care. Since we were aware of this before finalizing our search, we were able to address this circumstance in our search strategy. In addition, we were careful to apply a broad definition of IPC in advance so that the definitions and synonyms of the study authors could be subordinated. Nevertheless, this does not change the fact that the different wording can lead to difficulties in the classification of interventions and can make it difficult to reliably assess the effects of IPC in a comparative context. As a result, the classification of the interventions into the three types of interventions was not easily applicable, since in most cases combined interventions were used. IPC as a multicomponent intervention is difficult to delineate for this reason and thus makes it difficult to study its relative effectiveness.

In addition, the definition of PRO measures seems to vary as well [36]. For example, the question of whether satisfaction is a PRO is easier to answer than for functional outcomes, such as physical function. Whereas “satisfaction” cannot be answered without asking a patient, the outcome “physical functioning” such as the “mastery of activities of daily living,” can not only be answered by the patient himself, but it can also be observer ministered. This circumstance had to be considered in the selection of literature. Therefore, we decided to include all assessments in which the patients were asked to answer the question(s) and exclude all observer-ministered outcome measures. This is in line with the definition of the Food and Drug Administration (FDA) which defines a PRO as “any report of the status of a patient’s health condition that comes directly from the patient without interpretation of the patient’s response by a clinician or anyone else” [3]. However, proxy answers were allowed to avoid systematic exclusion of study populations who are not able to answer the questions themselves (old age, cognitive impairment, pediatric). Since relatives are closely involved in the treatment process and usually also play a decisive role in deciding it, they can equally be regarded as recipients of the healthcare services. There are two studies in which proxy answers were included in analysis. Firstly, Goldberg et al. [17] asked patients with cognitive impairment in old age (> 65 years old) as well as their proxies to report QoL (EQ-5D, EuroQuol-5D). No statistically significant effects have been found, neither in self-reports nor in proxy answers. Secondly, Hechler et al. [21] included patients aged 9 to 17 years and, among others, evaluated the “functional ability and health status” using the P-PDI (Pediatric Pain Disability Index). Whereas children aged 11 and older answered the questionnaire themselves, for children under 11, it was their parents who answered the P-PDI. Here, the description of results did not distinguish between self-, and proxy reports. Both studies have been marked with “proxy completed” in Table 2.

Nevertheless, as definitions of PRO measures vary across studies, one outcome measure can be observer ministered in one study and patient reported in another pointing out the important role of a sufficient validation in the respective application and study population. In the included studies of this review, there were 15 assessments in which scales were only implemented partially (14, 21, 24–26, 30–34) (see Additional file 6), and references to validation studies are missing in three studies [15, 16, 25].

This review has several limitations. First of all, our results are limited to the fact that included studies are conceptually heterogeneous and with high risk of bias, which was assessed by only one person. Only two studies [21, 27] have a low risk of bias, and a lot of different terms were used to describe IPC, and a lot of outcome measures were used to assess PRO. The included studies took place in ten different countries (Switzerland, Germany, Great Britain, Australia, Netherlands, Denmark, France, Italy, Norway, and USA) with different healthcare systems and different vocational trainings and professional roles. Additionally, only one study [21] reported treatment effects which were adjusted for multiple hypotheses testing, thus yielding the possibility of type 1 error inflation of the reported unadjusted effects. Therefore, quantitative meta-analysis was not feasible, and description of results is limited to the effect sizes which were reported in the studies. The results within some studies are ambiguous as well, for example, in cases where an outcome was assessed with several outcome measures. For this reason, the question whether interprofessional collaboration affect PRO cannot be answered conclusively. Nonetheless, most of the reported effect estimates suggest a positive effect on interprofessional interventions on PRO.

Secondly, psychometric properties of PRO measures as well as minimal important differences (MIDs) were not considered in presentation of results, although they are important when it comes to assessing whether the respective effects are also relevant from the patients’ perspective. However, we recorded which study reports validation studies to the outcome measures used and present our records in Additional file 6.

Previous reviews sought to measure the effect of IPC by focusing on objective patient outcomes [4, 37], collaborative behavior and team satisfaction [38], or specific settings and indications [39–42]. Our aim was to add the effects on PRO to the existing knowledge on the effectiveness of IPC. Even though it remains challenging to make a clear statement, this systematic review shows the current state of what has been established so far and thus points to the following research implications:

- Methodically rigorous studies are needed to contribute to the current state of literature and enable a reliable statement with regard to IPC. Specifically, randomized controlled trials reporting the underlying definition of IPC as well as the psychometric properties of PRO measures along with corresponding MIDs would be desirable. Especially in cases in which only single parts of questionnaires are used, the validity and reliability of measurement scales should be discussed.
- As our review highlighted the importance of standardized terminology, future studies are needed that focus on the definition and conceptualization of IPC.
- This review focused on inpatient care. For a comprehensive overview, the outpatient setting should be subject of future research.

## Conclusion

Twenty-two studies were included in this systematic review. There was a broad variety of different definitions of IPC, and studies covered a wide range of populations, interventions, indications, and outcomes. Thus, the high expected clinical heterogeneity and high RoB made it impractical to aggregate the treatment effect estimates statistically. While heterogenous effects depending on indication and outcome may be possible in the broader set of studies, the results considered here are indicative of a generally positive effect of IPC on PRO, irrespective of these observable study characteristics. Future methodically rigorous studies are needed to answer the question of effectiveness of IPC on PROs.

## Supplementary Information

**Additional file 1.** PRISMA Checklist.

**Additional file 2.** Search strategies.

**Additional file 3.** Excluded studies.

**Additional file 4.** Extraction.

**Additional file 5.** Quality assessment.

**Additional file 6.** Outcome Measures.

**Additional file 7.** Effects satisfaction.

**Additional file 8.** Effects functional ability.

**Additional file 9.** Effects pain.

**Additional file 10.** Effects psychiatric morbidity.

**Additional file 11.** Effects management of own healthcare.

**Additional file 12.** Effects therapeutic relationship.

**Additional file 13.** Effects treatment success.

## Data Availability

All data generated or analyzed during this study are included in this published article and its supplementary information files.

## Abbreviations

ADL: Activities of daily living
ADS: General Depression Scale (Allgemeine Depressions-Skala)
AEQ: Avoidance-endurance questionnaire
AQoL-4D: Assessment of quality of life
ASES: Arthritis Self-Efficacy Scale
Assign.: Assignment
BDI: Beck Depression Inventory
CBA: Controlled before-and-after studies
CLBP: Chronic low back pain
CP: Chronic pain
CSQ: Coping strategies questionnaire
CSQ-8: Client satisfaction questionnaire
DASS-21: Depression Anxiety Stress Scale
DEMQOL: Dementia quality of life measure
DIKJ: Depression inventory for children and adolescents
EC-17: Effective musculoskeletal consumer scale
EPOC: Cochrane Effective Practice and Organization of Care Group
ES: Effect sizes
ESAS: Edmonton System Assessment Scale
EORTC IN-PATSAT32: EORTC Inpatient Satisfaction with Cancer Care questionnaire
EORTC QLQ-C30: EORTC Quality of Life with Cancer Questionnaire
EQ VAS: EuroQol visual analogue scale
EQ-5D (-5L): EuroQol 5D (-5L, long version)
FACT-G: Functional Assessment of Cancer Therapy-General measure
FAMS: Functional Assessment of Multiple Sclerosis questionnaire
FDA: Food and Drug Administration
FESV: Pain management questionnaire
FFbH-R: Hannover Functional Ability Questionnaire-back pain
FFkA: Freiburg Questionnaire of physical activity
FIQ: Fibromyalgia Impact Questionnaire
FPS-R: Faces Pain Scale — Revised
GHQ-20: General Health Questionnaire
GPE: Global perceived effect
HADS: Hospital Anxiety and Depression Scale
HCAHPS: Hospital Consumer Assessment of Healthcare Providers and Systems
IADL: Independent activities of daily living
IPC: Interprofessional collaboration
I-PDQ-39: Italian 39-question Parkinson’s disease questionnaire
K10: Kessler psychological distress scale
LHS: London handicap scale
LSQ-G: German Life Satisfaction Questionnaire (Fragebogen zur Lebenszufriedenheit)
MCOHPQ: Modified City of Hope QoL Patient Questionnaire
MD: Mean differences
MDS-UPDRS: Italian Movement Disorder Society Unified Parkinson's Disease Rating Scale
MID: Minimal important differences
MLHF: Minnesota Living with Heart Failure questionnaire
MS: Multiple sclerosis
MSIS-29: Multiple Sclerosis Impact Scale-29 version 2
NRS: Non-randomized studies
PAM-SF: Patient Activation Measure (Short Form)
PCT: Palliative care team
PHQ-9: Patient Health Questionnaire
PD: Parkinson’s disease
PDCA: Plan-do-check-act cycle
PDI: Pain Disability Index
P-PDI: Pediatric pain disability index
PRCQ-C: Pain-related cognitions questionnaire for children
PRO: Patient-reported outcome(s)
PSEQ: Pain self-efficacy questionnaire
QPP: Quality of care from the patient’s perspective
RCT: Randomized controlled trial(s)
RoB: Risk of bias
ROBINS-I: Risk Of Bias in Non-randomized Studies of Interventions tool
SE: Standard error
SF-12: Short Form 12
SF-36: Short Form 36
QoL: Quality of life
SCL-90-R: Symptom Checklist-90-R
SES: Pain Perception Scale
WHO: World Health Organization
WHOQOL-BREF: World Health Organization Quality of Life Assessment scale
WHYMPI: West Haven-Yale Multidimensional Pain Inventory

## References

[1] Reeves S, Pelone F, Harrison R, Goldman J, Zwarenstein M. Interprofessional collaboration to improve professional practice and healthcare outcomes. Cochrane Database Syst Rev. 2017. 10.1002/14651858.CD000072.pub3. 10.1002/14651858.CD000072.pub3 PMC6481564 28639262

[2] Kaiser L, Bartz S, Neugebauer EAM, Pietsch B, Pieper D (2018). Interprofessional collaboration and patient-reported outcomes in inpatient care: protocol for a systematic review. Syst Rev.

[3] Johnston BC, Patrick DL, Devji T, Maxwell LJ, Bingham III CO, Beaton D, Boers M, Briel M, Busse JW, Carrasco-Labra A, Christensen R, da Costa BR, El Dib R, Lyddiatt A, Ostelo RW, Shea B, Singh J, Terwee CB, Williamson PR, Gagnier JJ, Tugwell P, Guyatt GH. Chapter 18: Patient-reported outcomes. In: Higgins JPT, Thomas J, Chandler J, Cumpston M, Li T, Page MJ, Welch VA (editors). Cochrane Handbook for Systematic Reviews of Interventions version 6.1 (updated September 2020). Cochrane, 2020. 2020. www.training.cochrane.org/handbook.

[4] Pannick S, Davis R, Ashrafian H, Byrne BE, Beveridge I, Athanasiou T (2015). Effects of interdisciplinary team care interventions on general medical wards: a systematic review. JAMA Intern Med.

[5] Moher D, Liberati A, Tetzlaff J, Altman DG (2009). Preferred Reporting Items for Systematic Reviews and Meta-Analyses: the PRISMA statement. BMJ..

[6] Cochrane Effective Practice and Organisation of Care Review Group (EPOC). Data Collection Checklist. 2002. https://methods.cochrane.org/sites/methods.cochrane.org.bias/files/public/uploads/EPOC%20Data%20Collection%20Checklist.pdf. Accessed 14 Nov 2020.

[7] Sterne JA, Hernán MA, Reeves BC, Savović J, Berkman ND, Viswanathan M (2016). ROBINS-I: a tool for assessing risk of bias in non-randomised studies of interventions. BMJ..

[8] McGowan J, Sampson M, Salzwedel DM, Cogo E, Foerster V, Lefebvre C (2016). PRESS Peer Review of Electronic Search Strategies: 2015 Guideline Statement. J Clin Epidemiol.

[9] Reeves S, Clark E, Lawton S, Ream M, Ross F. Examining the nature of interprofessional interventions designed to promote patient safety: a narrative review. Int J Qual Health Care. 2017;1–7. 10.1093/intqhc/mzx008. 10.1093/intqhc/mzx008 28453828

[10] Lidstone SC, Bayley M, Lang AE (2020). The evidence for multidisciplinary care in Parkinson’s disease. Expert Rev Neurother.

[11] Cochrane Effective Practice and Organisation of Care (EPOC). What study designs can be considered for inclusion in an EPOC review and what should they be called? 2017. https://epoc.cochrane.org/sites/epoc.cochrane.org/files/public/uploads/Resources-for-authors2017/what_study_designs_should_be_included_in_an_epoc_review.pdf. Accessed 11 Nov 2020.

[12] World Health Organization (WHO). List of Member States by WHO region and mortality stratum. https://www.who.int/choice/demography/mortality_strata/en/. Accessed 13 Feb 2021.

[13] Sterne JAC, Savović J, Page MJ, Elbers RG, Blencowe NS, Boutron I, et al. RoB 2: a revised tool for assessing risk of bias in randomised trials. BMJ. 2019;366. 10.1136/bmj.l4898. 10.1136/bmj.l4898 31462531

[14] Boesen F, Norgaard M, Trenel P, Rasmussen PV, Petersen T, Lovendahl B (2018). Longer term effectiveness of inpatient multidisciplinary rehabilitation on health-related quality of life in MS patients: a pragmatic randomized controlled trial - the Danish MS Hospitals Rehabilitation Study. Mult Scler J.

[15] Counsell SR, Holder CM, Liebenauer LL, Palmer RM, Fortinsky RH, Kresevic DM (2000). Effects of a multicomponent intervention on functional outcomes and process of care in hospitalized older patients: a randomized controlled trial of Acute Care for Elders (ACE) in a community hospital. J Am Geriatr Soc.

[16] Gade G, Venohr I, Conner D, McGrady K, Beane J, Richardson RH (2008). Impact of an inpatient palliative care team: a randomized controlled trial. J Palliat Med.

[17] Goldberg SE, Bradshaw LE, Kearney FC, Russell C, Whittamore KH, Foster PER (2013). Care in specialist medical and mental health unit compared with standard care for older people with cognitive impairment admitted to general hospital: randomised controlled trial (NIHR TEAM trial). BMJ..

[18] Hamnes B, Mowinckel P, Kjeken I, Hagen KB. Effects of a one week multidisciplinary inpatient self-management programme for patients with fibromyalgia: a randomised controlled trial. BMC Musculoskelet Disord. 2012;13. 10.1186/1471-2474-13-189. 10.1186/1471-2474-13-189 PMC3551734 23013162

[19] Cheung W, Aggarwal G, Fugaccia E, Thanakrishnan G, Milliss D, Anderson R (2010). Palliative care teams in the intensive care unit: a randomised, controlled, feasibility study. Crit Care Resusc.

[20] Grudzen CR, Richardson LD, Johnson PN, Hu M, Wang B, Ortiz JM (2016). Emergency department–initiated palliative care in advanced cancer: a randomized clinical trial. JAMA Oncol.

[21] Hechler T, Ruhe A-K, Schmidt P, Hirsch J, Wager J, Dobe M (2014). Inpatient-based intensive interdisciplinary pain treatment for highly impaired children with severe chronic pain: randomized controlled trial of efficacy and economic effects. Pain..

[22] Hewett N, Buchman P, Musariri J, Sargeant C, Johnson P, Abeysekera K (2016). Randomised controlled trial of GP-led in-hospital management of homeless people ('Pathway’). Clin Med.

[23] Mangels M, Schwarz S, Worringen U, Holme M, Rief W (2009). Evaluation of a behavioral-medical inpatient rehabilitation treatment including booster sessions: a randomized controlled study. Clin J Pain.

[24] Monticone M, Ambrosini E, Laurini A, Rocca B, Foti C (2015). In-patient multidisciplinary rehabilitation for Parkinson’s disease: a randomized controlled trial. Mov Disord.

[25] O’Leary KJ, Killarney A, Hansen LO, Jones S, Malladi M, Marks K (2016). Effect of patient-centred bedside rounds on hospitalised patients’ decision control, activation and satisfaction with care. BMJ Qual Saf.

[26] Sidebottom AC, Jorgenson A, Richards H, Kirven J, Sillah A (2015). Inpatient palliative care for patients with acute heart failure: outcomes from a randomized trial. J Palliat Med.

[27] Singer S, Danker H, Meixensberger J, Briest S, Dietz A, Kortmann R-D (2019). Structured multi-disciplinary psychosocial care for cancer patients and the perceived quality of care from the patient perspective: a cluster-randomized trial. J Cancer Res Clin Oncol.

[28] Wu J, Vratsistas-Curto A, Shiner CT, Faux SG, Harris I, Poulos CJ (2019). Can in-reach multidisciplinary rehabilitation in the acute ward improve outcomes for critical care survivors? A pilot randomized controlled trial. J Rehabil Med.

[29] Ziser K, Rheindorf N, Keifenheim K, Becker S, Resmark G, Giel KE, et al. Motivation-enhancing psychotherapy for inpatients with anorexia nervosa (MANNA): a randomized controlled pilot study. Front Psychiatry. 2021;12. 10.3389/fpsyt.2021.632660. 10.3389/fpsyt.2021.632660 PMC7882628 33597901

[30] Angst F, Verra ML, Lehmann S, Brioschi R, Aeschlimann A (2009). Clinical effectiveness of an interdisciplinary pain management programme compared with standard inpatient rehabilitation in chronic pain: a naturalistic, prospective controlled cohort study. J Rehabil Med.

[31] Brédart A, Dolbeault S, Savignoni A, Simard S, Gomme S, Asselain B (2009). Pilot evaluation of a French interdisciplinary supportive care department. Support Care Cancer.

[32] Hampel P, Tlach L (2015). Cognitive-behavioral management training of depressive symptoms among inpatient orthopedic patients with chronic low back pain and depressive symptoms: a 2-year longitudinal study. J Back Musculoskelet Rehabil.

[33] Semrau J, Hentschke C, Buchmann J, Meng K, Vogel H, Faller H, et al. Long-term effects of interprofessional biopsychosocial rehabilitation for adults with chronic non-specific low back pain: a multicentre, quasi-experimental study. PLoS One. 2015;10. 10.1371/journal.pone.0118609. 10.1371/journal.pone.0118609 PMC4359119 25768735

[34] Marcussen M, Norgaard B, Borgnakke K, Arnfred S. Improved patient-reported outcomes after interprofessional training in mental health: a nonrandomized intervention study. BMC Psychiatry. 2020;20. 10.1186/s12888-020-02616-x. 10.1186/s12888-020-02616-x PMC7227283 32410668

[35] Deenik J, Tenback DE, Tak ECPM, Hendriksen IJM, van Harten PN (2018). Improved psychosocial functioning and quality of life in inpatients with severe mental illness receiving a multidisciplinary lifestyle enhancing treatment. The MULTI study II. Ment Health Phys Act.

[36] Hamilton DF, Giesinger JM, Giesinger K. It is merely subjective opinion that patient-reported outcome measures are not objective tools. Bone Joint Res 2017;6:665–6. 10.1302/2046-3758.612.BJR-2017-0347. 10.1302/2046-3758.612.BJR-2017-0347 PMC5935812 29212762

[37] Parajuli DR, Kourbelis C, Franzon J, Newman P, Mckinnon RA, Shakib S (2019). Effectiveness of the pharmacist-involved multidisciplinary management of heart failure to improve hospitalizations and mortality rates in 4630 patients: a systematic review and meta-analysis of randomized controlled trials. J Card Fail.

[38] Wranik WD, Price S, Haydt SM, Edwards J, Hatfield K, Weir J (2019). Implications of interprofessional primary care team characteristics for health services and patient health outcomes: a systematic review with narrative synthesis. Health Policy.

[39] Lee H, Ryu K, Sohn Y, Kim J, Suh GY, Kim E (2019). Impact on patient outcomes of pharmacist participation in multidisciplinary critical care teams: a systematic review and meta-analysis. Crit Care Med.

[40] Patel JN, Klein DS, Sreekumar S, Liporace FA, Yoon RS (2020). Outcomes in multidisciplinary team-based approach in geriatric hip fracture care: a systematic review. J Am Acad Orthop Surg.

[41] Kamper SJ, Apeldoorn AT, Chiarotto A, Smeets RJEM, Ostelo RWJG, Guzman J (2015). Multidisciplinary biopsychosocial rehabilitation for chronic low back pain: Cochrane systematic review and meta-analysis. BMJ..

[42] Gougeon L, Johnson J, Morse H (2017). Interprofessional collaboration in health care teams for the maintenance of community-dwelling seniors’ health and well-being in Canada: a systematic review of trials. J Interprofessional Educ Pract.

