# Supplementary material for: Interprofessional collaboration and patient-reported outcomes in inpatient care: a systematic review
Source: Syst Rev. 2022 Aug 13;11:169. doi: 10.1186/s13643-022-02027-x (PMC9375378; doi:10.1186/s13643-022-02027-x)
Supplement: Supplementary file 2 — Additional file 2. Search strategies. [file 13643_2022_2027_MOESM2_ESM.docx]

**Additional file 2: Search strategies**

| **MEDLINE (PubMed) search strategy, searched on 21 July 2017 (1^st^) and 17 June 2019 (2^nd^)** | | | |
| --- | --- | --- | --- |
| **Concept** | **Search #** | **Search string** | **Hits** |
| Inpatient Care | 1  2 | Inpatients [MeSH]  ward* [ti,ab] |  |
| Interprofessional collaboration (IPC) | 3 | interprofessional relations [MeSH] |  |
|  | 4 | patient care team [MeSH] |  |
|  | 5 | intersectoral collaboration [MeSH] |  |
|  | 6 | team* [ti,ab] |  |
|  | 7 | cooperat* [ti,ab] |  |
|  | 8 | collaborat* [ti,ab] |  |
|  | 9 | transprofession* [ti,ab] |  |
|  | 10 | transdisciplinar* [ti,ab] |  |
|  | 11 | multiprofession* [ti,ab] |  |
|  | 12 | multidisciplinar* [ti,ab] |  |
|  | 13 | interprofession* [ti,ab] |  |
|  | 14 | interdisciplinar* [ti,ab] |  |
| Patient-reported outcomes (PRO),  Patient-reported experiences (PRE) | 15 | Patient Outcome Assessment [MeSH] |  |
|  | 16 | patient reported [ti,ab] |  |
|  | 17 | outcom* [ti,ab] |  |
|  | 18 | experienc* [ti,ab] |  |
|  | 19 | patients’ [ti,ab] |  |
|  | 20 | satisfact* [ti,ab] |  |
|  | 21 | rating* [ti,ab] |  |
|  | 22 | perspect* [ti,ab] |  |
|  | 23 | percept* [ti,ab] |  |
|  | 24 | quality of life [ti,ab] |  |
| Combination within concepts | 25  26 | 1 or 2  3 or 4 or 5 or 6 or 7 or 8 or 9 or 10 or 11 or 12 or 13 or 14 |  |
|  | 27 | 15 or 16 or 17 or 18 or 19 or 20 or 21 or 22 or 23 or 24 |  |
| Combination of concepts | 28 | 25 and 26 and 27 [year 1997-2017 (1^st^)/ 2017-2019 (2^nd^); humans; language English or German; clinical trial] | **2274 (1^st^), 433 (2^nd^)** |

| **Web of Science/SSCI search strategy, searched on 23 July 2017 (1^st^), 19 June 2019 (2^nd^), and 28 April 2021 (3^rd^)** | | | |
| --- | --- | --- | --- |
| **Concept** | **Search #** | **Search string** | **Hits** |
| Inpatient Care | 1  2 | Inpatient* [ts]  ward* [ts] |  |
| Interprofessional collaboration (IPC) | 3 | interprofessional relation* [ts] |  |
|  | 4 | patient care team [ts] |  |
|  | 5 | intersectoral collaboration* [ts] |  |
|  | 6 | team* [ts] |  |
|  | 7 | cooperat* [ts] |  |
|  | 8 | collaborat* [ts] |  |
|  | 9 | transprofession* [ts] |  |
|  | 10 | transdisciplinar* [ts] |  |
|  | 11 | multiprofession* [ts] |  |
|  | 12 | multidisciplinar* [ts] |  |
|  | 13 | interprofession* [ts] |  |
|  | 14 | interdisciplinar* [ts] |  |
| Patient-reported outcomes (PRO),  Patient-reported experiences (PRE) | 15 | Patient Outcome Assessment [ts] |  |
|  | 16 | patient reported [ts] |  |
|  | 17 | outcom* [ts] |  |
|  | 18 | experienc* [ts] |  |
|  | 19 | patients’ [ts] |  |
|  | 20 | satisfact* [ts] |  |
|  | 21 | rating* [ts] |  |
|  | 22 | perspect* [ts] |  |
|  | 23 | percept* [ts] |  |
|  | 24 | quality of life [ts] |  |
| Combination within concepts | 25  26 | 1 or 2  3 or 4 or 5 or 6 or 7 or 8 or 9 or 10 or 11 or 12 or 13 or 14 |  |
|  | 27 | 15 or 16 or 17 or 18 or 19 or 20 or 21 or 22 or 23 or 24 |  |
| Combination of concepts | 28 | 25 and 26 and 27 [year 1997-2017 (1^st^)/ 2017-2019 (2^nd^); language English or German; document type: article] | **3084 (1^st^), 983 (2^nd^), 1155 (3^rd^)** |

| **Cochrane Library (CENTRAL) search strategy, searched on 21 July 2017 (1^st^) and 17 June 2019 (2^nd^)** | | | |
| --- | --- | --- | --- |
| **Concept** | **Search #** | **Search string** | **Hits** |
| Inpatient Care | 1  2 | Inpatients [MeSH]  ward* [ti,ab] |  |
| Interprofessional collaboration (IPC) | 3 | interprofessional relations [MeSH] |  |
|  | 4 | patient care team [MeSH] |  |
|  | 5 | intersectoral collaboration [MeSH] |  |
|  | 6 | team* [ti,ab] |  |
|  | 7 | cooperat* [ti,ab] |  |
|  | 8 | collaborat* [ti,ab] |  |
|  | 9 | transprofession* [ti,ab] |  |
|  | 10 | transdisciplinar* [ti,ab] |  |
|  | 11 | multiprofession* [ti,ab] |  |
|  | 12 | multidisciplinar* [ti,ab] |  |
|  | 13 | interprofession* [ti,ab] |  |
|  | 14 | interdisciplinar* [ti,ab] |  |
| Patient-reported outcomes (PRO),  Patient-reported experiences (PRE) | 15 | Patient Outcome Assessment [MeSH] |  |
|  | 16 | patient reported [ti,ab] |  |
|  | 17 | outcom* [ti,ab] |  |
|  | 18 | experienc* [ti,ab] |  |
|  | 19 | patients’ [ti,ab] |  |
|  | 20 | satisfact* [ti,ab] |  |
|  | 21 | rating* [ti,ab] |  |
|  | 22 | perspect* [ti,ab] |  |
|  | 23 | percept* [ti,ab] |  |
|  | 24 | quality of life [ti,ab] |  |
| Combination within concepts | 25  26 | 1 or 2  3 or 4 or 5 or 6 or 7 or 8 or 9 or 10 or 11 or 12 or 13 or 14 |  |
|  | 27 | 15 or 16 or 17 or 18 or 19 or 20 or 21 or 22 or 23 or 24 |  |
| Combination of concepts | 28 | 25 and 26 and 27 [publication year 1997-2017 (1^st^)/ 2017-2019 (2^nd^); trials; without EMBASE or PubMed] | **60 (1^st^), 135 (2^nd^)** |

| **Current Contents (LIVIVO) search strategy, searched on 21 July 2017 (1^st^) and 17 June 2019 (2^nd^)** | | | |
| --- | --- | --- | --- |
| **Concept** | **Search #** | **Search string** | **Hits** |
| Inpatient Care | 1  2 | Inpatients [MeSH]  ward* [kw] |  |
| Interprofessional collaboration (IPC) | 3 | interprofessional relations [MeSH] |  |
|  | 4 | patient care team [MeSH] |  |
|  | 5 | intersectoral collaboration [MeSH] |  |
|  | 6 | team* [kw] |  |
|  | 7 | cooperat* [kw] |  |
|  | 8 | collaborat* [kw] |  |
|  | 9 | transprofession* [kw] |  |
|  | 10 | transdisciplinar* [kw] |  |
|  | 11 | multiprofession* [kw] |  |
|  | 12 | multidisciplinar* [kw] |  |
|  | 13 | interprofession* [kw] |  |
|  | 14 | interdisciplinar* [kw] |  |
| Patient-reported outcomes (PRO),  Patient-reported experiences (PRE) | 15 | Patient Outcome Assessment [MeSH] |  |
|  | 16 | patient reported [kw] |  |
|  | 17 | outcom* [kw] |  |
|  | 18 | experienc* [kw] |  |
|  | 19 | patients’ [kw] |  |
|  | 20 | satisfact* [kw] |  |
|  | 21 | rating* [kw] |  |
|  | 22 | perspect* [kw] |  |
|  | 23 | percept* [kw] |  |
|  | 24 | quality of life [kw] |  |
| Combination within concepts | 25  26 | 1 or 2  3 or 4 or 5 or 6 or 7 or 8 or 9 or 10 or 11 or 12 or 13 or 14 |  |
|  | 27 | 15 or 16 or 17 or 18 or 19 or 20 or 21 or 22 or 23 or 24 |  |
| Combination of concepts | 28 | 25 and 26 and 27 [year 1997-2017 (1^st^)/ 2017-2019 (2^nd^); database: current contents] | **4 (1^st^), 5 (2^nd^)** |

| **CINAHL (EBSCO) search strategy, searched on 20 July 2017 (1^st^) and 03 July 2019 (2^nd^)** | | | |
| --- | --- | --- | --- |
| **Concept** | **Search #** | **Search string** | **Hits** |
| Inpatient Care | 1  2 | Inpatients [MM]  ward* [ab] |  |
| Interprofessional collaboration (IPC) | 3 | interprofessional relations [MM] |  |
|  | 4 | multidisciplinary care team [MM] |  |
|  | 5 | collaboration [MM] |  |
|  | 6 | team* [ab] |  |
|  | 7 | cooperat*[ab] |  |
|  | 8 | collaborat*[ab] |  |
|  | 9 | transprofession*[ab] |  |
|  | 10 | transdisciplinar*[ab] |  |
|  | 11 | multiprofession*[ab] |  |
|  | 12 | multidisciplinar*[ab] |  |
|  | 13 | interprofession*[ab] |  |
|  | 14 | interdisciplinar*[ab] |  |
| Patient-reported outcomes (PRO),  Patient-reported experiences (PRE) | 15  16 | Outcome Assessment [MM]  Patient-Reported Outcomes [MM] |  |
|  | 17 | patient reported [ab] |  |
|  | 18 | outcom*[ab] |  |
|  | 19 | experienc*[ab] |  |
|  | 20 | patients’ [ab] |  |
|  | 21 | satisfact*[ab] |  |
|  | 22 | rating*[ab] |  |
|  | 23 | perspect*[ab] |  |
|  | 24 | percept*[ab] |  |
|  | 25 | quality of life [ab] |  |
| Combination within concepts | 26  27 | 1 or 2  3 or 4 or 5 or 6 or 7 or 8 or 9 or 10 or 11 or 12 or 13 or 14 |  |
|  | 28 | 15 or 16 or 17 or 18 or 19 or 20 or 21 or 22 or 23 or 24 or 25 |  |
| Combination of concepts | 29 | 26 and 27 and 28 [1997-2017 (1^st^)/ 2017-2019 (2^nd^); English or German] | **1472 (1^st^), 443 (2^nd^)** |

| **EMBASE search strategy, searched on 21 July 2017 (1^st^), 27 June 2019 (2^nd^), and 28 April 2021 (3^rd^)** | | | |
| --- | --- | --- | --- |
| **Concept** | **Search #** | **Search string** | **Hits** |
| Inpatient Care | 1 | hospital patient [exp] |  |
|  | 2 | ward* [ti,ab] |  |
| Interprofessional collaboration (IPC) | 3 | public relations [exp] |  |
|  | 4 | patient care [exp] |  |
|  | 5 | intersectoral collaboration [exp] |  |
|  | 6 | team* [ti,ab] |  |
|  | 7 | cooperat* [ti,ab] |  |
|  | 8 | collaborat* [ti,ab] |  |
|  | 9 | transprofession* [ti,ab] |  |
|  | 10 | transdisciplinar* [ti,ab] |  |
|  | 11 | multiprofession* [ti,ab] |  |
|  | 12 | multidisciplinar* [ti,ab] |  |
|  | 13 | interprofession* [ti,ab] |  |
|  | 14 | interdisciplinar* [ti,ab] |  |
| Patient-reported outcomes (PRO),  Patient-reported experiences (PRE) | 15 | outcome assessment [exp] |  |
|  | 16 | patient reported [ti,ab] |  |
|  | 17 | outcom* [ti,ab] |  |
|  | 18 | experienc* [ti,ab] |  |
|  | 19 | patients’ [ti,ab] |  |
|  | 20 | satisfact* [ti,ab] |  |
|  | 21 | rating* [ti,ab] |  |
|  | 22 | perspect* [ti,ab] |  |
|  | 23 | percept* [ti,ab] |  |
|  | 24 | quality of life [ti,ab] |  |
| Combination within concepts | 25 | 1 or 2 |  |
|  | 26 | 3 or 4 or 5 or 6 or 7 or 8 or 9 or 10 or 11 or 12 or 13 or 14 or 15 or 16 or 17 or 18 or 19 or 20 or 21 or 22 or 23 or 24 or 25 |  |
|  | 27 | 26 or 27 or 28 or 29 or 30 or 31 or 32 or 33 or 34 or 35 |  |
| Combination of concepts | 28 | 25 and 26 and 27 [year 1997-2017 (1^st^)/ 2017-2019 (2^nd^); language English or German] | **1400 (1^st^), 549 (2^nd^), 584 (3^rd^)** |
