# Supplementary material for: Interprofessional collaboration and patient-reported outcomes in inpatient care: a systematic review
Source: Syst Rev. 2022 Aug 13;11:169. doi: 10.1186/s13643-022-02027-x (PMC9375378; doi:10.1186/s13643-022-02027-x)
Supplement: Supplementary file 3 — Additional file 3. Excluded studies. [file 13643_2022_2027_MOESM3_ESM.docx]

**Additional file 5: Studies excluded in second screening**

1. Abrahamson K, Hass Z, Morgan K, Fulton B, Ramanujam R. The Relationship Between Nurse-Reported Safety Culture and the Patient Experience. JOURNAL OF NURSING ADMINISTRATION. 2016;46:662–8. (A10)
2. ACTRN12617000762358. Does an online diabetes education program for health care professionals improve inpatient diabetes care? 2017. Http://www.who.int/trialsearch/trial2.aspx? Trialid=actrn12617000762358. (A10)
3. ACTRN12617000832370. Effectiveness of cardiac rehabilitation program on subjective well-being of myocardial infarction patients. 2017. Http://www.who.int/trialsearch/trial2.aspx? Trialid=actrn12617000832370. (A7)
4. ACTRN12617001283369. Cancer And Physical ACtivITY (CAPACITY) trial: a randomised control trial of exercise and self-management for people with lung cancer. 2017. Http://www.who.int/trialsearch/trial2.aspx? Trialid=actrn12617001283369. (A1)
5. ACTRN12618000539235. Does very early therapy reduce the time spent hospital and improve the recovery of people with serious illness? 2018. Http://www.who.int/trialsearch/trial2.aspx? Trialid=actrn12618000539235. (A1)
6. Angeles RN, Guenter D, McCarthy L, Bauer M, Wolfson M, Chacon M, et al. Group interprofessional chronic pain management in the primary care setting: a pilot study of feasibility and effectiveness in a family health team in Ontario. Pain Res Manag. 2013;18:237–42. (A1)
7. Angst F, Gantenbein AR, Lehmann S, Gysi-Klaus F, Aeschlimann A, Michel BA, et al. Multidimensional associative factors for improvement in pain, function, and working capacity after rehabilitation of whiplash associated disorder: a prognostic, prospective outcome study. BMC MUSCULOSKELETAL DISORDERS. 2014;15. (A3)
8. Angst F., Brioschi R., Main C.J., Lehmann S., Aeschlimann A. Interdisciplinary Rehabilitation in Fibromyalgia and Chronic Back Pain: A Prospective Outcome Study. J Pain. 2006;7:807–15. (A3)
9. Arbaje A.I., Maron D.D., Yu Q., Wendel V.I., Tanner E., Boult C., et al. The geriatric floating interdisciplinary transition team. J Am Geriatr Soc. 2010;58:364–70. (A1)
10. Aripoli A.M., Fishback S.J., Morgan R.L., Hill J.D., Robinson A.L. Rounding Radiologists: Clinical Collaboration between Radiology Residents and Internal Medicine Teams. J Am Coll Radiol. 2016;13:562–5. (A1)
11. Auerbach AD, Sehgal NL, Blegen MA, Maselli J, Alldredge BK, Vittinghoff E, et al. Effects of a multicentre teamwork and communication programme on patient outcomes: results from the Triad for Optimal Patient Safety (TOPS) project. BMJ QUALITY & SAFETY. 2012;21:118–26. (A3)
12. Auerbach AD, Wachter RM, Cheng HQ, Maselli J, McDermott M, Vittinghoff E, et al. Comanagement of surgical patients between neurosurgeons and hospitalists. Arch Intern Med. 2010;170:2004–10. (A3)
13. Bacon CT, Hughes LC, Mark BA. Organizational influences on patient perceptions of symptom management. Res Nurs Health. 2009;32:321–34. (A1)
14. Bacon CT, Mark B. Organizational effects on patient satisfaction in hospital medical-surgical units. J Nurs Adm. 2009;39:220–7. (A1)
15. Baggs JG, Schmitt MH, Mushlin AI, Mitchell PH, Eldredge DH, Oakes D, et al. Association between nurse-physician collaboration and patient outcomes in three intensive care units. Critical Care Medicine. 1999;27. http://journals.lww.com/ccmjournal/Fulltext/1999/09000/Association_between_nurse_physician_collaboration.45.aspx. (A1)
16. Bailey J, Dijkers MP, Gassaway J, Thomas J, Lingefelt P, Kreider SED, et al. Relationship of nursing education and care management inpatient rehabilitation interventions and patient characteristics to outcomes following spinal cord injury: The SCIRehab project. Journal of Spinal Cord Medicine. 2012;35:593–610. (A1)
17. Bailey JE, Surbhi S, Wan JY, Munshi KD, Waters TM, Binkley BL, et al. Effect of Intensive Interdisciplinary Transitional Care for High-Need, High-Cost Patients on Quality, Outcomes, and Costs: a Quasi-Experimental Study. JOURNAL OF GENERAL INTERNAL MEDICINE. 2019;34:1815–24. (A1)
18. Bakitas M, Lyons K, Hegel MT, et al. Effects of a palliative care intervention on clinical outcomes in patients with advanced cancer: The project enable ii randomized controlled trial. JAMA. 2009;302:741–9. (A1)
19. Ballangrud R, Husebø SE, Aase K, Aaberg OR, Vifladt A, Berg GV, et al. “Teamwork in hospitals”: a quasi-experimental study protocol applying a human factors approach. BMC Nursing. 2017;16:1–7. (A5)
20. Bandemer-Greulich U, Bosse B, Fikentscher E, Konzag TA, Bahrke U. Efficacy of psychological interventions on pain coping strategies in orthopedic rehabilitation of chronic low back pain. PSYCHOTHERAPIE PSYCHOSOMATIK MEDIZINISCHE PSYCHOLOGIE. 2008;58:32–7. (A4)
21. Banez G.A., Frazier T.W., Wojtowicz A.A., Buchannan K., Henry D.E., Benore E. Chronic pain in children and adolescents: 24-42 month outcomes of an inpatient/day hospital interdisciplinary pain rehabilitation program. J Pediatr Rehabit Med. 2014;7:197–206. (A2)
22. Bano G, Dianin M, Biz C, Bedogni M, Alessi A, Bordignon A, et al. Efficacy of an interdisciplinary pathway in a first level trauma center orthopaedic unit: A prospective study of a cohort of elderly patients with hip fractures. ARCHIVES OF GERONTOLOGY AND GERIATRICS. 2020;86. (A10)
23. Baumgardt J, Schwarz J, Von Peter S, Holzke M, Längle G, Brieger P, et al. Crisis resolution teams in integrated care. Nervenheilkunde. 2020;39:739–45. (A5)
24. Beaird G, Baernholdt M, Do Byon H, White KR. Interprofessional rounding design features and association with collaboration and team effectiveness. JOURNAL OF INTERPROFESSIONAL CARE. (A3)
25. Berntsen GKR, Dalbakk M, Hurley JS, Bergmo T, Solbakken B, Spansvoll L, et al. Person-centred, integrated and pro-active care for multi-morbid elderly with advanced care needs: a propensity score-matched controlled trial. BMC HEALTH SERVICES RESEARCH. 2019;19. (A1)
26. Bhattacharya B, Davis KA. Geriatric Trauma Systems. Curr Trauma Rep. 2021;7:8–14. (A1)
27. Bilodeau BL, Stanford DA, Goldszmidt M, Appleton A. Simulated co-location of patients admitted to an inpatient internal medicine teaching unit: potential impacts on efficiency and physician-nurse collaboration. INFOR. 2020;58:109–23. (A1)
28. Black V, Bobier C, Thomas B, Prest F, Ansley C, Loomes B, et al. Reducing seclusion and restraint in a child and adolescent inpatient area: implementation of a collaborative problem-solving approach. AUSTRALASIAN PSYCHIATRY. 2020;28:578–84. (A3)
29. Blaettler LT, Stewart JA, Gubler DA, Egloff N, von Kanel R, Holtforth MG. Alexithymia moderates effects of psychotherapeutic treatment expectations on depression outcome in interdisciplinary chronic pain treatment. JOURNAL OF PSYCHOSOMATIC RESEARCH. 2019;122:69–72. (A10)
30. Boermeester MA. Effect of a ward-based pharmacy team on preventable adverse drug events in surgical patients (SUREPILL study). Br J Surg. 2015;102:1204–12. (A1)
31. Bogner J, Hade EM, Peng J, Beaulieu CL, Horn SD, Corrigan JD, et al. Family Involvement in Traumatic Brain Injury LO!) Inpatient Rehabilitation: A Propensity Score Analysis of Effects on Outcomes During the First Year After Discharge. ARCHIVES OF PHYSICAL MEDICINE AND REHABILITATION. 2019;100:1801–9. (A1)
32. Borg DN, Nielsen M, Kennedy A, Drovandi C, Beadle E, Bohan JK, et al. The effect of access to a designated interdisciplinary post-acute rehabilitation service on participant outcomes after brain injury. BRAIN INJURY. 2020;34:1358–66. (A1)
33. Bott N, Wexler S, Drury L, Pollak C, Wang V, Scher K, et al. A Protocol-Driven, Bedside Digital Conversational Agent to Support Nurse Teams and Mitigate Risks of Hospitalization in Older Adults: Case Control Pre-Post Study. JOURNAL OF MEDICAL INTERNET RESEARCH. 2019;21. (A1)
34. Brearly TW, Goodman CS, Haynes C, McDermott K, Rowland JA. Improvement of postinpatient psychiatric follow-up for veterans using telehealth. AMERICAN JOURNAL OF HEALTH-SYSTEM PHARMACY. 2020;77:288–94. (A3)
35. Bridges J, Griffiths P, Oliver E, Pickering RM. Hospital nurse staffing and staff-patient interactions: an observational study. BMJ QUALITY & SAFETY. 2019;28:706–13. (A1)
36. Brooke J, Herring S. Development of an activities care crew to support patients. Nursing Older People. 2016;28:20–5. (A10)
37. Brown T, May A, Beverley-Stone M. The adaptation and implementation of the Health Improvement Profile to Australian standards in public mental health settings. JOURNAL OF PSYCHIATRIC AND MENTAL HEALTH NURSING. 2020;27:628–39. (A1)
38. Browne AL, Appleton S, Fong K, Wood F, Coll F, de Munck S, et al. A pilot randomized controlled trial of an early multidisciplinary model to prevent disability following traumatic injury. DISABILITY AND REHABILITATION. 2013;35:1149–63. (A1)
39. Burkhardt H, Burger M. [Outcome and predictors of early geriatric rehabilitation in an acute care setting]. Z Gerontol Geriatr. 2012;45:138–45. (A1)
40. Burn E, Conneely M, Leverton M, Giacco D. Giving Patients Choices During Involuntary Admission: A New Intervention. FRONTIERS IN PSYCHIATRY. 2019;10. (A3)
41. Cabanel N., Kundermann B., Hautzinger M., Hornig W., Kuhl H., Mirk C., et al. MVT - A Multiprofessional Behavioural Therapy Program for Inpatient Treatment of Depression in Old Age. Psychiatr Prax. 2016;43:222–4. (A3)
42. Caceres JW, Alter SM, Shih RD, Fernandez JD, Williams FK, Paley R, et al. Standardized Physician-Administered Patient-Centered Discharge Protocol Improves Patients’ Comprehension. SOUTHERN MEDICAL JOURNAL. 2017;110:359–62. (A10)
43. Canales PL, Dorson PG, Crismon ML. Outcomes assessment of clinical pharmacy services in a psychiatric inpatient setting. Am J Health Syst Pharm. 2001;58:1309–16. (A1)
44. Capodaglio P, Cimolin V, Tacchini E, Precilios H, Brunani A. Effectiveness of in-patient rehabilitation in obesity-related orthopedic conditions. J Endocrinol Invest. 2013;36:628–31. (A3)
45. Carlton P, Pyle R. A program for parents of teens with anorexia nervosa and eating disorder not otherwise specified. INTERNATIONAL JOURNAL OF PSYCHIATRY IN CLINICAL PRACTICE. 2007;11:9–15. (A1)
46. Catangui EJ, Slark J. Nurse-led ward rounds: a valuable contribution to acute stroke care. British Journal of Nursing. 2012;21:801–5. (A1)
47. Cefalu C, Colbourne G, Duffy M, Johnson E, Lestter M, Wright J. A University-Affiliated Community Hospital Inpatient Geriatrics Program functioning in an administrative and educational capacity. JOURNAL OF THE AMERICAN GERIATRICS SOCIETY. 1997;45:355–60. (A3)
48. Chan AY, Vadera S. Implementation of interdisciplinary neurosurgery morning huddle: Cost-effectiveness and increased patient satisfaction. J Neurophysiol. 2018;128:258–61. (A3)
49. Chang C.F., Winsett R.P., Gaber A.O., Hathaway D.K. Cost-effectiveness of post-transplantation quality of life intervention among kidney recipients. Clin Transplant. 2004;18:407–14. (A1)
50. Chang H-H, Tsai S-L, Chen C-Y, Liu W-J. Outcomes of hospitalized elderly patients with geriatric syndrome: report of a community hospital reform plan in Taiwan. ARCHIVES OF GERONTOLOGY AND GERIATRICS. 2010;50:S30–3. (A7)
51. Cheung R., Hoffman R.S., Vlahov D., Manini A.F. Prognostic Utility of Initial Lactate in Patients With Acute Drug Overdose: A Validation Cohort. Ann Emerg Med. 2018;72:16–23. (A1)
52. Cheung W, Sahai V, Mann-Farrar J, Skylas K, Uy J, Doyle B. Incidents resulting from staff leaving normal duties to attend medical emergency team calls. Med J Aust. 2014;201:528–31. (A1)
53. Cheung W, Team CME. Outcomes following changing from a two-tiered to a three-tiered hospital rapid response system. AUSTRALIAN HEALTH REVIEW. 2017;43:178–87. (A10)
54. Chiu C-C, Wang J-J, Hung C-M, Lin H-F, Hsien H-H, Hung K-W, et al. Impact of multidisciplinary stroke post-acute care on cost and functional status: A prospective study based on propensity score matching. Brain Sci. 2021;11:1–10. (A7)
55. Choi N, Kim J, Kim H. The influence of patient-centeredness on patient safety perception among inpatients. PLOS ONE. 2021;16. (A3)
56. Clark LL, Lekkai F, Murphy A, Perrino L, Bapir-Tardy S, Barley EA. The use of positive behaviour support plans in mental health inpatient care: A mixed methods study. JOURNAL OF PSYCHIATRIC AND MENTAL HEALTH NURSING. 2020;27:140–50. (A3)
57. Cole M, McCusker J, Bellavance F, Primeau F, Bailey R, Bonnycastle M, et al. Systematic detection and multidisciplinary care of delirium in older medical inpatients: a randomized trial. CANADIAN MEDICAL ASSOCIATION JOURNAL. 2002;167:753–9. (A1)
58. Coleman SA, Cunningham CJ, Walsh JB, Coakley D, Harbison J, Casey M, et al. Outcomes among older people in a post-acute inpatient rehabilitation unit. Disabil Rehabil. 2012;34:1333–8. (A3)
59. Connor K I, Siebens HC, Mittman BS, Ganz DA, Barry F, Ernst EJ, et al. Quality and extent of implementation of a nurse-led care management intervention: care coordination for health promotion and activities in Parkinson’s disease (CHAPS). BMC HEALTH SERVICES RESEARCH. 2020;20. (A3)
60. Corbett HM, Lim WK, Davis SJ, Elkins AM. Care coordination in the Emergency Department: improving outcomes for older patients. Aust Health Rev. 2005;29:43–50. (A3)
61. Couppe C, Comins J, Beyer N, Hansen SE, Stodolsky DS, Siersma V. Health-related quality of life in patients with chronic rheumatic disease after a multidisciplinary rehabilitation regimen. QUALITY OF LIFE RESEARCH. 2017;26:381–91. (A3)
62. Cramer H, Hehlke M, Vasmer J, Rampp T, Anheyer D, Saha FJ, et al. Integrated care for migraine and chronic tension-type headaches: A prospective observational study. COMPLEMENTARY THERAPIES IN CLINICAL PRACTICE. 2019;36:1–6. (A1)
63. Cropp C, Streeck-Fischer A, Jaeger U, Masuhr O, Schroder A, Leichsenring F. [The relationship between the experience and success of inpatient psychotherapy treatment among children and adolescents]. Z Kinder Jugendpsychiatr Psychother. 2008;36:205–13. (A1)
64. Cuesta-Gomez A, Sanchez-Herrera-Baeza P, Ona-Simbana ED, Martinez-Medina A, Ortiz-Comino C, Balaguer-Bernaldo-de-Quiros C, et al. Effects of virtual reality associated with serious games for upper limb rehabilitation inpatients with multiple sclerosis: randomized controlled trial. JOURNAL OF NEUROENGINEERING AND REHABILITATION. 2020;17. (A1)
65. Curran JA, Breneol S, Vine J. Improving transitions in care for children with complex and medically fragile needs: a mixed methods study. BMC PEDIATRICS. 2020;20. (A1)
66. Czaplijski T, Marshburn D, Hobbs T, Bankard S, Bennett W. Creating a culture of mobility: an interdisciplinary approach for hospitalized patients. Hosp Top. 2014;92:74–9. (A1)
67. Dams-O’Connor K, Ketchum JM, Cuthbert JP, Corrigan JD, Hammond FM, Haarbauer-Krupa J, et al. Functional Outcome Trajectories Following Inpatient Rehabilitation for TBI in the United States: A NIDILRR TBIMS and CDC Interagency Collaboration. JOURNAL OF HEAD TRAUMA REHABILITATION. 2020;35:127–39. (A3)
68. Day C, Briskman J, Crawford MJ, Foote L, Harris L, Boadu J, et al. An intervention for parents with severe personality difficulties whose children have mental health problems: a feasibility RCT. HEALTH TECHNOLOGY ASSESSMENT. 2020;24:1+. (A3)
69. de Graaf MA, Antoni ML, ter Kuile MM, Arbous MS, Duinisveld AJF, Feltkamp MCW, et al. Short-term outpatient follow-up of COVID-19 patients: A multidisciplinary approach. EClinicalMedicine. 2021;32. doi:10.1016/j.eclinm.2021.100731. (A1)
70. Deenik J, Tenback DE, Tak ECPM, Henkemans OAB, Rosenbaum S, Hendriksen IJM, et al. Implementation barriers and facilitators of an integrated multidisciplinary lifestyle enhancing treatment for inpatients with severe mental illness: the MULTI study IV. BMC HEALTH SERVICES RESEARCH. 2019;19. (A3)
71. Del Giudice E, Ferretti E, Omiciuolo C, Sceusa R, Zanata C, Manganaro D, et al. The hospital-based, post-acute geriatric evaluation and management unit: the experience of the acute geriatric unit in Trieste. Arch Gerontol Geriatr. 2009;49 Suppl 1:49–60. (A3)
72. Demartini B, Batla A, Petrochilos P, Fisher L, Edwards MJ, Joyce E. Multidisciplinary treatment for functional neurological symptoms: a prospective study. J Neurol. 2014;261:2370–7. (A3)
73. Deschodt M, Braes T, Broos P, Sermon A, Boonen S, Flamaing J, et al. Effect of an Inpatient Geriatric Consultation Team on Functional Outcome, Mortality, Institutionalization, and Readmission Rate in Older Adults with Hip Fracture: A Controlled Trial. Journal of the American Geriatrics Society. 2011;59:1299–308. (A1)
74. Deschodt M, Jeuris A, Van Grootven B, Van Waerebeek E, Gantois E, Flamaing J, et al. Adherence to recommendations of inpatient geriatric consultation teams: a multicenter observational study. EUROPEAN GERIATRIC MEDICINE. 2021;12:175–84. (A3)
75. Dinius J, Philippe R, Ernstmann N, Heier L, Goeritz AS, Pfisterer-Heise S, et al. Inter-professional teamwork and its association with patient safety in German hospitals-A cross sectional study. PLOS ONE. 2020;15. (A1)
76. Dobbins M, Gunson J, Bale S, Neary M, Ingrams D, Brown M. Head and neck nursing. Improving patient care and quality of life after laryngectomy/glossectomy. British Journal of Nursing. 2005;14:634–40. (A10)
77. Dorscht L, Schoen C, Geiss C, Graessel E, Donath C. Access to Pain Management Programs: A Multifactorial Analysis of the Pathways of Care for Chronic Pain Patients in the University Clinic Erlangen. GESUNDHEITSWESEN. 2020;82:E94–107. (A1)
78. Dougherty M.J. Client satisfaction survey of inpatient trauma and dissociative disorders program. J Trauma Dissociation. 2002;3:97–105. (A3)
79. Ebrahimi Z, Eklund K, Dahlin-Ivanoff S, Jakobsson A, Wilhelmson K. Effects of a continuum of care intervention on frail elders’ self-rated health, experiences of security/safety and symptoms: A randomised controlled trial. Nordic Journal of Nursing Research. 2017;37:33–43. (A6)
80. Ebrahimi Z, Eklund K, Dahlin-Ivanoff S, Jakobsson A, Wilhelmson K. Effects of a continuum of care intervention on frail elders’ self-rated health, experiences of security/safety and symptoms: A randomised controlled trial. Nordic Journal of Nursing Research. 2017;37:33–43. (A2)
81. Ekerstad N, Ostberg G, Johansson M, Karlson BW. Are frail elderly patients treated in a CGA unit more satisfied with their hospital care than those treated in conventional acute medical care? PATIENT PREFERENCE AND ADHERENCE. 2018;12:233–40. (A4)
82. Eklund K, Wilhelmson K, Gustafsson H, Landahl S, Dahlin-Ivanoff S. One-year outcome of frailty indicators and activities of daily living following the randomised controlled trial: “Continuum of care for frail older people”. BMC Geriatr. 2013;13:76. (A2)
83. Ellis TE, Rufino KA, Allen JG. A controlled comparison trial of the Collaborative Assessment and Management of Suicidality (CAMS) in an inpatient setting: Outcomes at discharge and six-month follow-up. PSYCHIATRY RESEARCH. 2017;249:252–60. (A1)
84. Engel PT, Thavayogarajah T, Goerlich D, Lenz P. Establishment of a Palliative Care Consultation Service (PCCS) in an Acute Hospital Setting. INTERNATIONAL JOURNAL OF ENVIRONMENTAL RESEARCH AND PUBLIC HEALTH. 2020;17. (A3)
85. Engelhardt J.B., Rizzo V.M., Della Penna R.D., Feigenbaum P.A., Kirkland K.A., Nicholson J.S., et al. Effectiveness of care coordination and health counseling in advancing illness. Am J Managed Care. 2009;15:817–25. (A2)
86. Faerden A, Bolgen B, Lovhaug L, Thoresen C, Dieset I. Patient satisfaction and acute psychiatric inpatient treatment. NORDIC JOURNAL OF PSYCHIATRY. 2020;74:577–84. (A3)
87. Fagerberg B, Claesson L, Gosman-Hedström G, Blomstrand C, Fagerberg B, Claesson L, et al. Effect of acute stroke unit care integrated with care continuum versus conventional treatment: A randomized 1-year study of elderly patients: the Göteborg 70+ Stroke Study. Stroke (00392499). 2000;31:2578–84. (A4)
88. Ferguson A, Coates DE, Osborn S, Blackmore CC, Williams B. Early, Nurse-Directed Sepsis Care. AMERICAN JOURNAL OF NURSING. 2019;119:52–8. (A10)
89. Fielding R, Kause J, Arnell-Cullen V, Sandeman D. The impact of consultant-delivered multidisciplinary inpatient medical care on patient outcomes. Clin Med (Lond). 2013;13:344–8. (A1)
90. Fowler R, Congdon P, Hamilton S. Assessing health status and outcomes in a geriatric day hospital. PUBLIC HEALTH. 2000;114:440–5. (A2)
91. Frojd C, Swenne CL, Rubertsson C, Gunningberg L, Wadensten B. Patient information and participation still in need of improvement: evaluation of patients’ perceptions of quality of care. J Nurs Manag. 2011;19:226–36. (A1)
92. Fuller TE, Pong DD, Piniella N, Pardo M, Bessa N, Yoon C, et al. Interactive Digital Health Tools to Engage Patients and Caregivers in Discharge Preparation: Implementation Study. JOURNAL OF MEDICAL INTERNET RESEARCH. 2020;22. (A3)
93. Gaertner J., Siemens W., Meerpohl J.J., Antes G., Meffert C., Xander C., et al. Effect of specialist palliative care services on quality of life in adults with advanced incurable illness in hospital, hospice, or community settings: Systematic review and meta-analysis. BMJ (Online). 2017;357; doi:10.1136/bmj.j2925. (A3)
94. Garduño-López AL, Nava VMA, Garcés LC, Martínez DMR, Cuellarguzmán LF, Villanueva MEF, et al. Towards better perioperative pain management in Mexico: A study in a network of hospitals using quality improvement methods from pain out. J Pain Res. 2021;14: 415–30. (A7)
95. Geller J, Maiolino N, Samson L, Srikameswaran S. Is experiencing care as collaborative associated with enhanced outcomes in inpatient eating disorders treatment? EATING DISORDERS. (A3)
96. Gill FJ, Leslie GD, Marshall AP. Parent escalation of care for the deteriorating child in hospital: A health-care improvement study. HEALTH EXPECTATIONS. 2019;22:1078–88. (A3)
97. Glick AF, Goonan M, Sherman J, Sandmeyer D, Gold-von Simson G. Parent Perspectives on Participation in Family-Centered Rounds and Informational Resource Use. Front Pediatr. 2020;8. doi:10.3389/fped.2020.00343. (A3)
98. Glinka L, Januszkiewicz J, Gutysz-Wojnicka A, Karakina N, Braczkowska M, Żechowicz M. Expectations of families of patients hospitalized in intensive care units. Pol Ann Med. 2020;27:21–6. (A7)
99. Gormley DK, Costanzo AJ, Goetz J, Israel J, Hill-Clark J, Pritchard T, et al. Impact of Nurse-Led Interprofessional Rounding on Patient Experience. NURSING CLINICS OF NORTH AMERICA. 2019;54:115+. (A4)
100. Goyal A, Glanzman H, Quinn M, Tur K, Singh S, Winter S, et al. Do bedside whiteboards enhance communication in hospitals? An exploratory multimethod study of patient and nurse perspectives. BMJ QUALITY & SAFETY. 2020;29:795–802. (A3)
101. Graven C., Brock K., Hill K.D., Cotton S., Joubert L. First year after stroke: An integrated approach focusing on participation goals aiming to reduce depressive symptoms. Stroke. 2016;47:2820–7. (A2)
102. Groeneveld IF, Goossens PH, van Braak I, van der Pas S, Meesters JJL, Mishre RDR, et al. Patient’s outcome expectations and their fulfilment in multidisciplinary stroke rehabilitation. ANNALS OF PHYSICAL AND REHABILITATION MEDICINE. 2019;62:21–7. (A3)
103. Gruther W, Pieber K, Steiner I, Hein C, Hiesmayr JM, Paternostro-Sluga T. Can Early Rehabilitation on the General Ward After an Intensive Care Unit Stay Reduce Hospital Length of Stay in Survivors of Critical Illness?: A Randomized Controlled Trial. Am J Phys Med Rehabil. 2017;96:607–15. (A1)
104. Guhn A, Koehler S, Brakemeier E-L, Sterzer P. Cognitive Behavioral Analysis System of Psychotherapy for inpatients with persistent depressive disorder: a naturalistic trial on a general acute psychiatric unit. EUROPEAN ARCHIVES OF PSYCHIATRY AND CLINICAL NEUROSCIENCE. 2021;271:495–505. (A3)
105. Gupta N, Brown C, Deneke J, Maha J, Kong M. Utilization of a Novel Pathway in a Tertiary Pediatric Hospital to Meet the Sensory Needs of Acutely III Pediatric Patients. FRONTIERS IN PEDIATRICS. 2019;7. (A1)
106. Gupta R, Fitzgibbons C, Ramsay C, Vanderheiden L, Toppozini C, Lobos A-T. Development and pilot of an interprofessional pediatric resuscitation program for non-acute care inpatient providers. MEDICAL EDUCATION ONLINE. 2019;24. (A1)
107. Gutierrez-Valencia M, Izquierdo M, Beobide-Telleria I, Ferro-Uriguen A, Alonso-Renedo J, Casas-Herrero A, et al. Medicine optimization strategy in an acute geriatric unit: The pharmacist in the geriatric team. GERIATRICS & GERONTOLOGY INTERNATIONAL. 2019;19:530–6. (A3)
108. Haapamaki J, Heikkinen E, Sipponen T, Roine RP, Arkkila P. The impact of an adaptation course on health-related quality of life and functional capacity of patients with inflammatory bowel disease. SCANDINAVIAN JOURNAL OF GASTROENTEROLOGY. 2018;53:1074–8. (A1)
109. Haefner J, Dunn I, McFarland M. A Quality Improvement Project Using Verbal De-Escalation to Reduce Seclusion and Patient Aggression in an Inpatient Psychiatric Unit. ISSUES IN MENTAL HEALTH NURSING. 2021;42:138–44. (A1)
110. Haggarty J., O’Connor B., Dubois S., Blackadar A.M., McKinnon T., Boudreau D., et al. A pilot study of a Canadian shared mental health care programme: Changes in patient symptoms and disability. Prim Care Community Psychiatry. 2008;13:27–35. (A10)
111. Hallin K, Henriksson P, Dalen N, Kiessling A. Effects of interprofessional education on patient perceived quality of care. Med Teach. 2011;33:e22-26. (A1)
112. Halvorsen MR, Austad HO, Landmark AD, Ausen D, Svagård I, Tomasevic T, et al. Redesigning Work With a Lightweight Approach to Coordination Technology. CIN: Computers, Informatics, Nursing. 2019;37:124–32. (A1)
113. Hang Mui So. Evaluation of the Effect of a Critical Care Follow-up Program on Patient Outcomes. Evaluation of the Effect of a Critical Care Follow-Up Program on Patient Outcomes. 2017; Ph.D.:1–1.(A10)
114. Hanks GW, Robbins M, Sharp D, Forbes K, Done K, Peters TJ, et al. The imPaCT study: a randomised controlled trial to evaluate a hospital palliative care team. Br J Cancer. 2002;87:733–9. (A3)
115. Hart T, Ferraro M, Rabinowitz A, Fitzpatrick DeSalme E, Nelson L, Marcy E, et al. Improving communication with patients in post-traumatic amnesia: development and impact of a clinical protocol. BRAIN INJURY. 2020;34:1518–24. (A1)
116. Hartelt E, Scherbaum R, Kinkel M, Gold R, Muhlack S, Toenges L. Parkinson’s Disease Multimodal Complex Treatment (PD-MCT): Analysis of Therapeutic Effects and Predictors for Improvement. JOURNAL OF CLINICAL MEDICINE. 2020;9. (A3)
117. Harwood RH, Goldberg SE, Whittamore KH, Russell C, Gladman JR, Jones RG, et al. Evaluation of a Medical and Mental Health Unit compared with standard care for older people whose emergency admission to an acute general hospital is complicated by concurrent “confusion”: a controlled clinical trial. Acronym: TEAM: Trial of an Elderly Acute care Medical and mental health unit. Trials. 2011;12:123. (A5)
118. Haubitz-Eschelbach A, Mirsada D, Sebastian H, Alexander K, Beat M, Jeffrey GL, et al. The glory of the age is the wisdom of grey hair: association of physician appearance with outcomes in hospitalised medical patients - an observational study. SWISS MEDICAL WEEKLY. 2019;149. (A3)
119. Haugum M, Iversen HH, Helgeland J, Lindahl AK, Bjertnaes O. Patient experiences with interdisciplinary treatment for substance dependence: an assessment of quality indicators based on two national surveys in Norway. PATIENT PREFERENCE AND ADHERENCE. 2019;13:453–64. (A1)
120. Hebert C, Behel JM, Pal G, Kasi R, Kompoliti K. Multidisciplinary inpatient rehabilitation for Functional Movement Disorders: A prospective study with long term follow up. PARKINSONISM & RELATED DISORDERS. 2021;82:50–5. (A3)
121. Heinemann AW, Nitsch KP, Ehrlich-Jones L, Malamut L, Semik P, Srdanovic N, et al. Effects of an Implementation Intervention to Promote Use of Patient-Reported Outcome Measures on Clinicians’ Perceptions of Evidence-Based Practice, Implementation Leadership, and Team Functioning. JOURNAL OF CONTINUING EDUCATION IN THE HEALTH PROFESSIONS. 2019;39:103–11. (A10)
122. Higaonna M, Morimoto T, Ueda S. Association between nursing care delivery models and patients’ health outcomes in a university hospital: A retrospective cohort study based on the Diagnostic Procedure Combination database. JAPAN JOURNAL OF NURSING SCIENCE. 2020;17. (A1)
123. Hino K, Nomoto M, Endo E, Watanabe A, Sakamoto O, Takahashi Y. Effect of a Psychiatric Department Liaison Team on Patient Treatment and Outcomes. PSYCHIATRIC ANNALS. 2020;50:355–63. (A10)
124. Howard I, Potts A. Interprofessional Care for Neuromuscular Disease. Curr Treat Options Neurol. 2019;21. doi:10.1007/s11940-019-0576-z. (A1)
125. Huffman JC, Mastromauro CA, Beach SR, Celano CM, DuBois CM, Healy BC, et al. Collaborative Care for Depression and Anxiety Disorders in Patients With Recent Cardiac Events The Management of Sadness and Anxiety in Cardiology (MOSAIC) Randomized Clinical Trial. JAMA INTERNAL MEDICINE. 2014;174:927–35. (A1)
126. Huffman JC, Mastromauro CA, Sowden G, Fricchione GL, Healy BC, Januzzi JL. Impact of a depression care management program for hospitalized cardiac patients. Circ Cardiovasc Qual Outcomes. 2011;4:198–205. (A1)
127. Ishii M., Okumura Y., Sugiyama N., Hasegawa H., Noda T., Hirayasu Y., et al. Feasibility and efficacy of shared decision making for first-admission schizophrenia: A randomized clinical trial. BMC Psychiatry. 2017;17. doi:10.1186/s12888-017-1218-1. (A1)
128. Jakobsson L, Holmberg L. Quality from the patient’s perspective: a one-year trial. Int J Health Care Qual Assur. 2012;25:177–88. (A1)
129. Jepegnanam C, Bull E, Bansal S, McCarthy D, Booth M, Purser E, et al. The role of the psychologist in the inpatient pain service: development and initial outcomes. Br J Pain. 2020; doi:10.1177/2049463720926212. (A1)
130. Johansen I, Lindbaek M, Stanghelle JK, Brekke M. Structured community-based inpatient rehabilitation of older patients is better than standard primary health care rehabilitation: an open comparative study. Disabil Rehabil. 2012;34:2039–46. (A1)
131. Johansson C, Dahl J, Jannert M, Melin L, Andersson G. Effects of a cognitive-behavioral pain-management program. BEHAVIOUR RESEARCH AND THERAPY. 1998;36:915–30. (A1)
132. Johnson S, Nolan F, Pilling S, Sandor A, Hoult J, McKenzie N, et al. Randomised controlled trial of acute mental health care by a crisis resolution team: the north Islington crisis study. BRITISH MEDICAL JOURNAL. 2005;331:599–602. (A2)
133. Jonasdottir RJ, Jones C, Sigurdsson GH, Jonsdottir H. Structured nurse-led follow-up for patients after discharge from the intensive care unit: Prospective quasi-experimental study. JOURNAL OF ADVANCED NURSING. 2018;74:709–23. (A1)
134. Jones CH, O’Neill S, McLean KA, Wigmore SJ, Harrison EM. Patient experience and overall satisfaction after emergency abdominal surgery. BMC Surg. 2017;17:76. (A1)
135. Jordhoy MS, Fayers P, Loge JH, Ahlner-Elmqvist M, Kaasa S. Quality of life in palliative cancer care: results from a cluster randomized trial. J Clin Oncol. 2001;19:3884–94. (A2)
136. Jorgenson A, Sidebottom AC, Richards H, Kirven J. A Description of Inpatient Palliative Care Actions for Patients With Acute Heart Failure. Am J Hosp Palliat Care. 2016;33:863–70. (A1)
137. Kalra L, Evans A, Perez I, Knapp M, Swift C, Donaldson N. A randomised controlled comparison of alternative strategies in stroke care. Health Technol Assess. 2005;9:iii–iv, 1–79. (A1)
138. Karlsson A, Lindelof N, Olofsson B, Berggren M, Gustafson Y, Nordstrom P, et al. Effects of Geriatric Interdisciplinary Home Rehabilitation on Independence in Activities of Daily Living in Older People With Hip Fracture: A Randomized Controlled Trial. ARCHIVES OF PHYSICAL MEDICINE AND REHABILITATION. 2020;101:571–8. (A1)
139. Kaselionyte J, Conneely M, Giacco D. “It’s a matter of building bridges ... “ - feasibility of a carer involvement intervention for inpatients with severe mental illness. BMC PSYCHIATRY. 2019;19. (A3)
140. Katon WJ, Lin EHB, Von Korff M, Ciechanowski P, Ludman EJ, Young B, et al. Collaborative Care for Patients with Depression and Chronic Illnesses. N Engl J Med. 2010;363:2611–20. (A1)
141. Kawabata N, Nin M. Effect of continual quality improvement of palliative care consultation teams by iterative, customer satisfaction survey-driven evaluation. BMC PALLIATIVE CARE. 2021;20. (A1)
142. Kempen TGH, Kalvemark A, Gillespie U, Stewart D. Comprehensive medication reviews by ward-based pharmacists in Swedish hospitals: What does the patient have to say? JOURNAL OF EVALUATION IN CLINICAL PRACTICE. 2020;26:149–57. (A3)
143. Kerper LF, Spies CD, Salz A-L, Weiß-Gerlach E, Balzer F, Neumann T, et al. Effects of an Innovative Psychotherapy Program for Surgical Patients: Bridging Intervention in Anesthesiology--A Randomized Controlled Trial. Anesthesiology. 2015;123:148–59. (A1)
144. Khan F, Amatya B, Drummond K, Galea M. EFFECTIVENESS OF INTEGRATED MULTIDISCIPLINARY REHABILITATION IN PRIMARY BRAIN CANCER SURVIVORS IN AN AUSTRALIAN COMMUNITY COHORT: A CONTROLLED CLINICAL TRIAL. JOURNAL OF REHABILITATION MEDICINE. 2014;46:754–60. (A2)
145. King CA, Klaus N, Kramer A, Venkataraman S, Quinlan P, Gillespie B. The Youth-Nominated Support Team-Version II for Suicidal Adolescents: A Randomized Controlled Intervention Trial. JOURNAL OF CONSULTING AND CLINICAL PSYCHOLOGY. 2009;77:880–93. (A2)
146. King R, Seeger T, Wang M, Shan RLP, McGovern C, Knox J, et al. Early Supported Discharge for Neurorehabilitation Following Acquired Brain Injury. FRONTIERS IN NEUROLOGY. 2020;11. (A3)
147. Kinoshita S, Abo M, Okamoto T. Effectiveness of ICF-based multidisciplinary rehabilitation approach with serial assessment and discussion using the ICF rehabilitation set in a convalescent rehabilitation ward. INTERNATIONAL JOURNAL OF REHABILITATION RESEARCH. 2020;43:255–60. (A10)
148. Kircher TTJ, Wormstall H, Muller PH, Schwarzler F, Buchkremer G, Wild K, et al. A randomised trial of a geriatric evaluation and management consultation services in frail hospitalised patients. Age Ageing. 2007;36:36–42. (A1)
149. Kirk J, Collins K. Difference in quality of life of referred hospital patients after hospital palliative care team intervention. S Afr Med J. 2006;96:101–2. (A3)
150. Klinger R, Nutzinger D, Geissner E, Hafenbrack K, Hahn B, Apelt M. Follow-up results of an in patient behavioral pain treatment program. ZEITSCHRIFT FUR KLINISCHE PSYCHOLOGIE-FORSCHUNG UND PRAXIS. 1999;28:267–72. (A1)
151. Kominski G, Andersen R, Bastani R, Gould R, Hackman C, Huang D, et al. UPBEAT: The impact of a psychogeriatric intervention in VA medical centers. MEDICAL CARE. 2001;39:500–12. (A2)
152. Konieczny M, Cipora E, Sawicka J, Fal A. Patient satisfaction with oncological care during the sars-cov-2 virus pandemic. Int J Environ Res Public Health. 2021;18. doi:10.3390/ijerph18084122. (A7)
153. Krawczyk M, Sawatzky R. Relational use of an electronic quality of life and practice support system in hospital palliative consult care: A pilot study. PALLIATIVE & SUPPORTIVE CARE. 2019;17:208–13. (A3)
154. Laguna J., Goldstein R., Allen J., Braun W., Enguídanos S. Inpatient palliative care and patient pain: Pre- and post-outcomes. J Pain Symptom Manage. 2012;43:1051–9. (A3)
155. Laird-Fick HS, Solomon D, Jodoin C, Dwamena FC, Alexander K, Rawsthorne L, et al. Training residents and nurses to work as a patient-centered care team on a medical ward. Patient Education & Counseling. 2011;84:90–7. (A1)
156. Lawton R, O’Hara JK, Sheard L, Armitage G, Cocks K, Buckley H, et al. Can patient involvement improve patient safety? A cluster randomised control trial of the Patient Reporting and Action for a Safe Environment (PRASE) intervention. BMJ Quality & Safety. 2017;26:622–31. (A1)
157. Lechien JR, Ducarme M, Place S, Chiesa-Estomba CM, Khalife M, De Riu G, et al. Objective olfactory findings in hospitalized severe COVID-19 patients. Pathogens. 2020;9:1–6. (A3)
158. Lee MK, Yih Y, Griffin PM. Quantifying the Impact of Acute Stroke System of Care Transfer Protocols on Patient Outcomes. MEDICAL DECISION MAKING. 2020;40:873–84. (A3)
159. Lee Y-Y, Lin JL. Do patient autonomy preferences matter? Linking patient-centered care to patient–physician relationships and health outcomes. Social Science & Medicine. 2010;71:1811–8. (A1)
160. Lehmann A I, Brauchli R, Bauer GF. Goal Pursuit in Organizational Health Interventions: The Role of Team Climate, Outcome Expectancy, and Implementation Intentions. FRONTIERS IN PSYCHOLOGY. 2019;10. (A1)
161. Lehmann LS, Brancati FL, Chen MC, Roter D, Dobs AS. The effect of bedside case presentations on patients’ perceptions of their medical care. N Engl J Med. 1997;336:1150–5. (A1)
162. Leziak K, Yee LM, Grobman WA, Badreldin N. Patient Experience with Postpartum Pain Management in the Face of the Opioid Crisis. JOURNAL OF MIDWIFERY & WOMENS HEALTH. (A3)
163. Lidstone SC, Bayley M, Lang AE. The evidence for multidisciplinary care in Parkinson’s disease. EXPERT REVIEW OF NEUROTHERAPEUTICS. 2020;20:539–49. (A3)
164. Lindpaintner LS, Gasser JT, Schramm MS, Cina-Tschumi B, Muller B, Beer JH. Discharge intervention pilot improves satisfaction for patients and professionals. Eur J Intern Med. 2013;24:756–62. (A1)
165. Lisby M, Thomsen A, Nielsen LP, Lyhne NM, Breum-Leer C, Fredberg U, et al. The effect of systematic medication review in elderly patients admitted to an acute ward of internal medicine. Basic Clin Pharmacol Toxicol. 2010;106:422–7. (A1)
166. LITAKER D, MION LC, PLANAVSKY L, KIPPES C, MEHTA N, FROLKIS J. Physician–nurse practitioner teams in chronic disease management: the impact on costs, clinical effectiveness, and patients’ perception of care. Journal of Interprofessional Care. 2003;17:223–37. (A2)
167. Liu M, McCurry SM, Belza B, Dobra A, Buchanan DT, Vitiello MV, et al. Effects of Osteoarthritis Pain and Concurrent Insomnia and Depression on Health Care Use in a Primary Care Population of Older Adults. ARTHRITIS CARE & RESEARCH. 2019;71:748–57. (A1)
168. Lloyd-Evans B, Johnson S, Morant N, Gilburt H, Osborn DPJ, Jagielska D, et al. Alternatives to standard acute in-patient care in England: differences in content of care and staff-patient contact. Br J Psychiatry Suppl. 2010;53:s46-51. (A2)
169. Locher C, Mansour R, Koechlin H, Buechi S. Patient-appraised beneficial moments during inpatient psychiatric treatment. BMC HEALTH SERVICES RESEARCH. 2020;20. (A3)
170. Lok SD, Marciniuk J, Baranyi M, Hasany A, Marciniuk DD. Enhancing pulmonary rehabilitation enrollment study (PRESS III). Can J Respir Crit Care Sleep Med. 2020; doi:10.1080/24745332.2020.1743212. (A1)
171. Lorenzen Molina K, Schibella Souto de Moura GM. Patient satisfaction of hospital stay according to the form at a teaching hospital. Acta Paulista de Enfermagem. 2016;29:17–25. (A1)
172. Loustalot M-C, Berdot S, Sabatier P, Durieux P, Perrin G, Karras A, et al. The impact of interventions by pharmacists collected in a computerised physician order entry context: a prospective observational study with a 10-year reassessment. SWISS MEDICAL WEEKLY. 2019;149. (A1)
173. Lu Chen. Effectiveness of a Patient-Centered Self-Management Empowerment Intervention during Transition Care on Stroke Survivors. Effectiveness of a Patient-Centered Self-Management Empowerment Intervention During Transition Care on Stroke Survivors. 2017; Ph.D.:1–1. (A10)
174. Luthy C., Gerstel P.F., Pugliesi A., Piguet V., Allaz A.-F., Cedraschi C. Bedside or not bedside: Evaluation of patient satisfaction in intensive medical rehabilitation wards. PLoS ONE. 2017;12. doi:10.1371/journal.pone.0170474. (A1)
175. Maben J, Adams M, Peccei R, Murrells T, Robert G. “Poppets and parcels”: the links between staff experience of work and acutely ill older peoples’ experience of hospital care. Int J Older People Nurs. 2012;7:83–94. (A1)
176. Mackie BR, Marshall AP, Mitchell ML. Exploring family participation in patient care on acute care wards: A mixed-methods study. INTERNATIONAL JOURNAL OF NURSING PRACTICE. 2021;27. (A1)
177. Mai M V, Orenstein EW, Manning JD, Luberti AA, Dziorny AC. Attributing Patients to Pediatric Residents Using Electronic Health Record Features Augmented with Audit Logs. APPLIED CLINICAL INFORMATICS. 2020;11:442–51. (A10)
178. Major J, Varga ZK, Gyimesi-Szikszai A, Adam S. A two-week inpatient programme with a booster improved long-term management of severe chronic paediatric pain. JOURNAL OF CHILD HEALTH CARE. 2017;21:171–80. (A3)
179. Manges K, Groves PS, Farag A, Peterson R, Harton J, Greysen SR. A mixed methods study examining teamwork shared mental models of interprofessional teams during hospital discharge. BMJ QUALITY & SAFETY. 2020;29:499–508. (A1)
180. Mar S.H., Leng T.Y., Yan J., Oo A.M., Wai B.C., Sien N.Y. Early oncology rehabilitation (EOR) for lymphoma patients: A review of functional outcomes and inpatient rehabilitation pilot program in Singapore General Hospital (SGH). J Pain Manage. 2018;11:123–32. (A10)
181. Marchetta CM, Maruyama R, Galifi L, O’Reilly C. Evaluating a multidisciplinary inpatient program for youth with type 1 diabetes mellitus. PEDIATRIC DIABETES. 2020;21:1232–9. (A3)
182. Marcussen M, Norgaard B, Borgnakke K, Arnfred S. Interprofessional clinical training in mental health improves students’ readiness for interprofessional collaboration: a non-randomized intervention study. BMC MEDICAL EDUCATION. 2019;19. (A1)
183. Martin-Rodriguez L.S., D’Amour D., Leduc N. Outcomes of interprofessional collaboration for hospitalized cancer patients. Cancer Nurs. 2008;31:E18–27. (A1)
184. Martínez-Velilla N, Garrués-Irisarri M, Ibañez-Beroiz B, Gil-Cabañas J, Richarte-García A, Idoate-Saralegui F, et al. An exercise program with patient’s involvement and family support can modify the cognitive and affective trajectory of acutely hospitalized older medical patients: a pilot study. Aging Clinical & Experimental Research. 2016;28:483–90. (A1)
185. Mascaro JS, Waller AV, Wright L, Leonard T, Haack C, Waller EK. Individualized, Single Session Yoga Therapy to Reduce Physical and Emotional Symptoms in Hospitalized Hematological Cancer Patients. INTEGRATIVE CANCER THERAPIES. 2019;18:1–8. (A1)
186. Mast LJ, Rahman A, Bridges D, Horslex NLD. Exploring the impact of an interprofessional care protocol on the patient experience and outcomes for seniors with diabetes. Patient Experience Journal. 2014;1:117–23. (A2)
187. McAlearney AS, Fareed N, Gaughan A, MacEwan SR, Volney J, Sieck CJ. Empowering Patients during Hospitalization: Perspectives on Inpatient Portal Use. APPLIED CLINICAL INFORMATICS. 2019;10:103–12. (A1)
188. McAlearney AS, Gaughan A, MacEwan SR, Fareed N, Huerta TR. Improving Acceptance of Inpatient Portals: Patients’ and Care Team Members’ Perspectives. ℡EMEDICINE AND E-HEALTH. 2020;26:310–26. (A1)
189. Meterko M, Mohr D, Young G. Teamwork culture and patient satisfaction in hospitals. MEDICAL CARE. 2004;42:492–8. (A1)
190. Michael R, Wheeler B, Wichmann H, Horner B, Downie J. The healthy ageing unit: a comparative controlled intervention. Journal of the Australasian Rehabilitation Nurses’ Association (JARNA). 2005;8:9–15. (A1)
191. Mirkovic B, Cohen D, de la Riviere SG, Pellerin H, Guile J-M, Consoli A, et al. Repeating a suicide attempt during adolescence: risk and protective factors 12 months after hospitalization. EUROPEAN CHILD & ADOLESCENT PSYCHIATRY. 2020;29:1729–40. (A1)
192. Mitchell SE, Martin JM, Krizman K, Sadikova E, Culpepper L, Stewart SK, et al. y Design and rationale for a randomized controlled trial to reduce readmissions among patients with depressive symptoms. CONTEMPORARY CLINICAL TRIALS. 2015;45 B:151–6. (A1)
193. Mohammadipour F, Atashzadeh‐Shoorideh F, Parvizy S, Hosseini M. An explanatory study on the concept of nursing presence from the perspective of patients admitted to hospitals. Journal of Clinical Nursing (John Wiley & Sons, Inc). 2017;26:4313–24. (A3)
194. Molony B, Horgan S, Graham I. Patient perceptions of the cardiology ward round. Ir Med J. 2012;105:189–90. (A10)
195. Mudge A, Laracy S, Richter K, Denaro C. Controlled trial of multidisciplinary care teams for acutely ill medical inpatients: enhanced multidisciplinary care. Intern Med J. 2006;36:558–63. (A4)
196. Mudge A.M., Denaro C.P., O’rourke P. Improving hospital outcomes in patients admitted from residential aged care: Results from a controlled trial. Age Ageing. 2012;41:670–3. (A3)
197. Mudge AM, McRae P, Donovan PJ, Reade MC. Multidisciplinary quality improvement programme for older patients admitted to a vascular surgery ward*. INTERNAL MEDICINE JOURNAL. 2020;50:741–8. (A1)
198. Mueller K, Hamilton G, Rodden B, DeHeer HD. Functional Assessment and Intervention by Nursing Assistants in Hospice and Palliative Care Inpatient Care Settings: A Quality Improvement Pilot Study. Am J Hosp Palliat Care. 2016;33:136–43. (A1)
199. Mulugeta H, Afenigus AD, Wagnew F, Haile D, Tadesse A, Kibret GD. The effect of hourly nursing rounds on patient satisfaction at Debre Markos Referral Hospital, Northwest Ethiopia: A non-randomized controlled clinical trial. Int J Afr Nurs Sci. 2020;13 doi:10.1016/j.ijans.2020.100239. (A7)
200. Natale JE, Boehmer J, Blumberg DA, Dimitriades C, Hirose S, Kair LR, et al. Interprofessional/interdisciplinary teamwork during the early COVID-19 pandemic: experience from a children’s hospital within an academic health center. JOURNAL OF INTERPROFESSIONAL CARE. 2020;34:682–6. (A1)
201. Nicholas MK, Asghari A, Sharpe L, Beeston L, Brooker C, Glare P, et al. Reducing the use of opioids by patients with chronic pain: an effectiveness study with long-term follow-up. PAIN. 2020;161:509–19. (A3)
202. Nielsen C, Siersma V, Ghaziani E, Beyer N, Magnusson SP, Couppe C. Health-Related Quality of Life and Physical Function in Individuals with Parkinson’s Disease after a Multidisciplinary Rehabilitation Regimen-A Prospective Cohort Feasibility Study. INTERNATIONAL JOURNAL OF ENVIRONMENTAL RESEARCH AND PUBLIC HEALTH. 2020;17. (A3)
203. Nielsen CM, Hjorthoj C, Killaspy H, Nordentoft M. The effect of flexible assertive community treatment in Denmark: a quasi-experimental controlled study. LANCET PSYCHIATRY. 2021;8:27–35. (A10)
204. Nijhawan AE, Bhattatiry M, Chansard M, Zhang S, Halm EA. HIV care cascade before and after hospitalization: impact of a multidisciplinary inpatient team in the US South. AIDS CARE-PSYCHOLOGICAL AND SOCIO-MEDICAL ASPECTS OF AIDS/HIV. 2020;32:1343–52. (A1)
205. Nikolaus T, Jamour M. [Effectiveness of special stroke units in treatment of acute stroke]. Z Gerontol Geriatr. 2000;33:96–101. (A10)
206. Norman E, Sherburn M, Osborne RH, Galea MP. An exercise and education program improves well-being of new mothers: a randomized controlled trial. Phys Ther. 2010;90:348–55. (A1)
207. Oslin D, Thompson R, Kallan M, TenHave T, Blow F, Bastani R, et al. Treatment effects from UPBEAT: A randomized trial of care management for behavioral health problems in hospitalized elderly patients. JOURNAL OF GERIATRIC PSYCHIATRY AND NEUROLOGY. 2004;17:99–106. (A1)
208. Ostovari M, Yu D. Impact of care provider network characteristics on patient outcomes: Usage of social network analysis and a multi-scale community detection. PLOS ONE. 2019;14. (A1)
209. Oyeflaten I, Hysing M, Eriksen HR. Prognostic factors associated with return to work following multidisciplinary vocational rehabilitation. JOURNAL OF REHABILITATION MEDICINE. 2008;40:548–54. (A1)
210. Oyeflaten I, Midtgarden IJ, Maeland S, Eriksen HR, Magnussen LH. Functioning, coping and work status three years after participating in an interdisciplinary, occupational rehabilitation program. SCANDINAVIAN JOURNAL OF PUBLIC HEALTH. 2014;42:425–33. (A1)
211. Palese A, Comuzzi C, Bresadola V. Global case management: the “nurse case manager” model applied to day surgery in Italy. Lippincotts Case Manag. 2005;10:83–92. (A10)
212. Pannill FC. In older hospitalized patients, adding transitional care to in-hospital geriatric assessment did not improve ADL. ACP Journal Club. 2016;164:1–1. (A5)
213. Pękacz A, Kądalska E, Skoczylas A, Targowski T. Patient satisfaction as an element of healthcare quality - A single-center Polish survey. Reumatologia. 2019;57:135–44. (A7)
214. Peltan ID, Poll JB, Guidry D, Brown SM, Beninati W. Acceptability and Perceived Utility of Telemedical Consultation during Cardiac Arrest Resuscitation A Multicenter Survey. ANNALS OF THE AMERICAN THORACIC SOCIETY. 2020;17:321–8. (A1)
215. Phelan EA, Balderson B, Levine M, Erro JH, Jordan L, Grothaus L, et al. Delivering effective primary care to older adults: A randomized, controlled trial of the senior resource team at group health cooperative. JOURNAL OF THE AMERICAN GERIATRICS SOCIETY. 2007;55:1748–56. (A1)
216. Pitkanen A, Alanen H-M, Kampman O, Leinonen E. Outcome of neuropsychiatric symptoms and daily functioning of patients with dementia treated on an acute psychogeriatric ward. NORDIC JOURNAL OF PSYCHIATRY. 2019;72:521–5. (A1)
217. Pitkanen A, Alanen H-M, Kampman O, Suontaka-Jamalainen K, Leinonen E. Implementing physical exercise and music interventions for patients suffering from dementia on an acute psychogeriatric inpatient ward. NORDIC JOURNAL OF PSYCHIATRY. 2019;73:401–8. (A1)
218. Porter J, Hanna L. Evidence-based analysis of protected mealtime policies on patient nutrition and care. Risk Manage Healthc Policy. 2020;13: 713–21. (A3)
219. Portillo MC, Corchon S, Lopez-Dicastillo O, Cowley S. Evaluation of a nurse-led social rehabilitation programme for neurological patients and carers: An action research study. INTERNATIONAL JOURNAL OF NURSING STUDIES. 2009;46:204–19. (A1)
220. Potter EL, Lew TE, Sooriyakumaran M, Edwards AM, Tong E, Aungu AK. Evaluation of pharmacist-led physician-supported inpatient deprescribing model in older patients admitted to an acute general medical unit. AUSTRALASIAN JOURNAL ON AGEING. 2019;38:206–10. (A1)
221. Pretz C, Kowalski RG, Cuthbert JP, Whiteneck GG, Miller AC, Ketchum JM, et al. Return to Productivity Projections for Individuals With Moderate to Severe TBI Following Inpatient Rehabilitation: A NIDILRR TBIMS and CDC Interagency Collaboration. JOURNAL OF HEAD TRAUMA REHABILITATION. 2020;35:140–51. (A1)
222. Qian C., Zhong D., Shen Y., Du Q. Evaluation of clinical efficacy of transitional care mode for patients with strokes. Int J Clin Exp Med. 2019;12:981–8. (A2)
223. Ramsay P, Huby G, Merriweather J, Salisbury L, Rattray J, Griffith D, et al. Patient and carer experience of hospital-based rehabilitation from intensive care to hospital discharge: mixed methods process evaluation of the RECOVER randomised clinical trial. BMJ OPEN. 2016;6. (A1)
224. Ravid NL, Zamora K, Rehm R, Okumura M, Takayama J, Kaiser S. Implementation of a multidisciplinary discharge videoconference for children with medical complexity: A pilot study. Pilot Feasibility Stud. 2020;6. doi:10.1186/s40814-020-00572-7. (A1)
225. Razon AN, Greenberg A, Trachtenberg S, Stollon N, Wu K, Ford L, et al. A Multidisciplinary Transition Consult Service: Patient Referral Characteristics. JOURNAL OF PEDIATRIC NURSING-NURSING CARE OF CHILDREN & FAMILIES. 2019;47:136–41. (A1)
226. Richards DA, Bower P, Chew-Graham C, Gask L, Lovell K, Cape J, et al. Clinical effectiveness and cost-effectiveness of collaborative care for depression in UK primary care (CADET): a cluster randomised controlled trial. HEALTH TECHNOLOGY ASSESSMENT. 2016;20:1+. (A1)
227. Rodgers H, Atkinson C, Bond S, Suddes M, Dobson R, Curless R. Randomized controlled trial of a comprehensive stroke education program for patients and caregivers. STROKE. 1999;30:2585–91. (A1)
228. Rosen P, Stenger E, Bochkoris M, Hannon MJ, Kwoh CK. Family-centered multidisciplinary rounds enhance the team approach in pediatrics. Pediatrics. 2009;123:e603-8. (A1)
229. Royse L, Nolan N, Hoffman K. Using a Sociogram to Characterize Communication During an Interprofessional Team Huddle. JOURNAL OF MULTIDISCIPLINARY HEALTHCARE. 2020;13:1583–93. (A1)
230. Russell BS, Guite JW, Homan KJ, Tepe RM, Williams SE. Complementary Parent Components for Pediatric Pain Families: Innovations in Treatment. CHILDREN-BASEL. 2020;7. (A1)
231. Salhofer-Polanyi S, Windt J, Sumper H, Grill H, Essmeister M, Diermayr G, et al. Benefits of inpatient multidisciplinary rehabilitation in multiple sclerosis. NeuroRehabilitation. 2013;33:285–92. (A1)
232. Saltvedt I., Jordhøy M., Mo E.-S.O., Fayers P., Kaasa S., Sletvold O. Randomised trial of in-hospital geriatric intervention: Impact on function and morale. Gerontology. 2006;52:223–30. (A4)
233. Scherbaum R, Hartelt E, Kinkel M, Gold R, Muhlack S, Toenges L. Parkinson’s Disease Multimodal Complex Treatment improves motor symptoms, depression and quality of life. JOURNAL OF NEUROLOGY. 2020;267:954–65. (A3)
234. Schindl M, Wassipaul S, Wagner T, Gstaltner K, Bethge M. Impact of Functional Capacity Evaluation on Patient-Reported Functional Ability: An Exploratory Diagnostic Before-After Study. JOURNAL OF OCCUPATIONAL REHABILITATION. 2019;29:711–7. (A3)
235. Schmidt H, Boese S, Bauer A, Landenberger M, Lau A, Stoll O, et al. Interdisciplinary care programme to improve self-management for cancer patients undergoing stem cell transplantation: a prospective non-randomised intervention study. EUROPEAN JOURNAL OF CANCER CARE. 2017;26. (A1)
236. Scovil CY, Delparte JJ, Walia S, Flett HM, Guy SD, Wallace M, et al. Implementation of Pressure Injury Prevention Best Practices Across 6 Canadian Rehabilitation Sites: Results From the Spinal Cord Injury Knowledge Mobilization Network. ARCHIVES OF PHYSICAL MEDICINE AND REHABILITATION. 2019;100:327–35. (A3)
237. Seitz T, Stastka K, Schiffinger M, Turk BR, Loeffler-Stastka H. Interprofessional care improves health-related well-being and reduces medical costs for chronic pain patients. BULLETIN OF THE MENNINGER CLINIC. 2019;83:105–27. (A3)
238. Sharda N, Mattoon E, Matters L, Prewitt J, McDonald S, Sloane R, et al. Bach to the Basics: Implementation and Impact of a Postoperative, Inpatient Personalized Music Program for Older Adults. JOURNAL OF PERIANESTHESIA NURSING. 2019;34:347–53. (A1)
239. Sharpe M., Walker J., Hansen C.H., Martin P., Symeonides S., Gourley C., et al. Integrated collaborative care for comorbid major depression in patients with cancer (SMaRT Oncology-2): a multicentre randomised controlled effectiveness trial. Lancet. 2014; (Sharpe M., michael.sharpe@psych.ox.ac.uk; Walker J.) Psychological Medicine Research, University of Oxford Department of Psychiatry, Warneford Hospital, Oxford, UK. doi:10.1016/S0140-6736(14)61231-9. (A2)
240. Shaygan M, Boeger A, Kroener-Herwig B. Predicting factors of outcome in multidisciplinary treatment of chronic neuropathic pain. JOURNAL OF PAIN RESEARCH. 2018;11:2433–43. (A3)
241. Shaygan M, Boger A, Kroner-Herwig B. How does reduction in pain lead to reduction in disability in patients with musculoskeletal pain? JOURNAL OF PAIN RESEARCH. 2019;12:1879–90. (A3)
242. Shike C. An Inpatient and Oupatient Nursing Collaborative Improves the Patient Experience. Oncol Issues. 2018;33:18–23. (A5)
243. Shyu Y-IL, Liang J, Wu C-C, Su J-Y, Cheng H-S, Chou S-W, et al. A Pilot Investigation of the Short-Term Effects of an Interdisciplinary Intervention Program on Elderly Patients with Hip Fracture in Taiwan. Journal of the American Geriatrics Society. 2005;53:811–8. (A1)
244. Skagseth M, Fimland MS, Rise MB, Johnsen R, Borchgrevink PC, Aasdahl L. Effectiveness of adding a workplace intervention to an inpatient multimodal occupational rehabilitation program: A randomized clinical trial. SCANDINAVIAN JOURNAL OF WORK ENVIRONMENT & HEALTH. 2020;46:356–63. (A1)
245. Skudlik C., Weisshaar E., Scheidt R., Elsner P., Wulfhorst B., Schönfeld M., et al. First results from the multicentre study Rehabilitation of Occupational Skin Diseases - Optimization and Quality Assurance of Inpatient Management (ROQ). Contact Dermatitis. 2012;66:140–7. (A3)
246. Skudlik C., Weisshaar E., Scheidt R., Wulfhorst B., Elsner P., Schönfeld M., et al. Current state and intermediate results from the multicentre study “Rehabilitation of Occupational Skin Diseases - Optimisation and Quality Assurance of Inpatient Management (ROQ).” Dermatol Beruf Umwelt. 2013;61:28–31. (A10)
247. Slaets J, Kauffmann R, Duivenvoorden H, Pelemans W, Schudel W. A randomized trial of geriatric liaison intervention in elderly medical inpatients. PSYCHOSOMATIC MEDICINE. 1997;59:585–91. (A1)
248. Sowden GL, Mastromauro CA, Seabrook RC, Celano CM, Rollman BL, Huffman JC. Baseline physical health-related quality of life and subsequent depression outcomes in cardiac patients. Psychiatry Res. 2013;208:288–90. (A1)
249. Spencer S, Stephens K, Swanson-Biearman B, Whiteman K. HEALTH CARE PROVIDER IN TRIAGE TO IMPROVE OUTCOMES. JOURNAL OF EMERGENCY NURSING. 2019;45:561+. (A3)
250. Sprave T, Nicolay NH, Grosu A-L, Lindenmeier J, Tscheulin DK. The introduction of a permanent survey system to measure the quality of life of ENT patients at the Department of Radiotherapy at the University Hospital of Freiburg: Implementation Report. Gesundh.okon Qual.manage. 2020;25:195–200. (A3)
251. Staines A, Lecureux E, Rubin P, Baralon C, Farin A. Impact of TeamSTEPPS on patient safety culture in a Swiss maternity ward. INTERNATIONAL JOURNAL FOR QUALITY IN HEALTH CARE. 2020;32:618–24. (A1)
252. Stewart D.G., Drake D.F., Robertson C., Marwitz J.H., Kreutzer J.S., Cifu D.X. Benefits of an inpatient pulmonary rehabilitation program: A prospective analysis. Arch Phys Med Rehabil. 2001;82:347–52. (A3)
253. Straub C, Bode SFN. Patients’ and parents’ percetion of care on a paediatric interprofessional training ward. BMC MEDICAL EDUCATION. 2019;19. (A3)
254. Sundberg F, Fridh I, Lindahl B, Kareholt I. Visitor’s Experiences of an Evidence-Based Designed Healthcare Environment in an Intensive Care Unit. HERD-HEALTH ENVIRONMENTS RESEARCH & DESIGN JOURNAL. (A1)
255. Surgery and Pharmacy in Liaison (SUREPILL) Study Group. Effect of a ward-based pharmacy team on preventable adverse drug events in surgical patients (SUREPILL study). Br J Surg. 2015;102:1204–12. (A1)
256. Suriyaarachchi P, Chu L, Bishop A, Thew T, Matthews K, Cowan R, et al. Evaluating Effectiveness of an Acute Rehabilitation Program in Hospital-Associated Deconditioning. JOURNAL OF GERIATRIC PHYSICAL THERAPY. 2020;43:172–8. (A10)
257. Swinnen N, Vandenbulcke M, de Bruin ED, Akkerman R, Stubbs B, Firth J, et al. The efficacy of exergaming in people with major neurocognitive disorder residing in long-term care facilities: a pilot randomized controlled trial. ALZHEIMERS RESEARCH & THERAPY. 2021;13. (A1)
258. Szczepanska-Gieracha J, Mazurek J. The Role of Self-Efficacy in the Recovery Process of Stroke Survivors. PSYCHOLOGY RESEARCH AND BEHAVIOR MANAGEMENT. 2020;13:897–906. (A1)
259. Takahashi N, Takatsuki K, Kasahara S, Yabuki S. Multidisciplinary pain management program for patients with chronic musculoskeletal pain in Japan: a cohort study. JOURNAL OF PAIN RESEARCH. 2019;12:2563–76. (A3)
260. Talbot R, Malas N. Addressing mental health stigma: A pilot educational video intervention for caregivers to facilitate psychiatric consultation in inpatient pediatric care settings. CLINICAL CHILD PSYCHOLOGY AND PSYCHIATRY. 2019;24:754–66. (A1)
261. Tanaka S, Ishikawa E, Mochida A, Kawano K, Kobayashi M. Effects of Early-Stage Group Psychoeducation Programme for Patients with Depression. OCCUPATIONAL THERAPY INTERNATIONAL. 2015;22:195–205. (A1)
262. Teeling SP, Coetzee H, Phillips M, McKiernan M, She EN, Igoe A. Reducing risk of development or exacerbation of nutritional deficits by optimizing patient access to mealtime assistance. INTERNATIONAL JOURNAL FOR QUALITY IN HEALTH CARE. 2019;31:6–13. (A1)
263. Theodoridou A, Hengartner MP, Gairing SK, Jaeger M, Ketteler D, Kawohl W, et al. Evaluation of a New Person-Centered Integrated Care Model in Psychiatry. PSYCHIATRIC QUARTERLY. 2015;86:153–68. (A1)
264. Theurna M, Read J, Moskowitz A, Stewart A. Evaluation of a New Zealand early intervention service for psychosis. NEW ZEALAND JOURNAL OF PSYCHOLOGY. 2007;36:136–45. (A2)
265. Thoegersen MH, Morthorst BR, Nordentoft M. Assertive community treatment versus standard treatment for severely mentally ill patients in Denmark: a quasi-experimental trial. NORDIC JOURNAL OF PSYCHIATRY. 2019;73:149–58. (A1)
266. Thomas KP, Salas RE, Gamaldo C, Chik Y, Huffman L, Rasquinha R, et al. Sleep rounds: a multidisciplinary approach to optimize sleep quality and satisfaction in hospitalized patients. J Hosp Med. 2012;7:508–12. (A1)
267. Tijhuis G, Zwinderman A, Hazes J, Breedveld F, Vlieland P. Two-year follow-up of a randomized controlled trial of a clinical nurse specialist intervention, inpatient, and day patient team care in rheumatoid arthritis. JOURNAL OF ADVANCED NURSING. 2003;41:34–43. (A1)
268. Tijhuis GJ, Zwinderman AH, Hazes JMW, Van Den Hout WB, Breedveld FC, Vliet Vlieland TPM. A randomized comparison of care provided by a clinical nurse specialist, an inpatient team, and a day patient team in rheumatoid arthritis. Arthritis Rheum. 2002;47:525–31. (A1)
269. Tinland A, Loubiere S, Boucekine M, Boyer L, Fond G, Girard V, et al. Effectiveness of a housing support team intervention with a recovery-oriented approach on hospital and emergency department use by homeless people with severe mental illness: a randomised controlled trial. EPIDEMIOLOGY AND PSYCHIATRIC SCIENCES. 2020;29. (A1)
270. Treadwell M, Franck L, Vichinsky E. Using quality improvement strategies to enhance pediatric pain assessment. INTERNATIONAL JOURNAL FOR QUALITY IN HEALTH CARE. 2002;14:39–47. (A1)
271. Tremblay D, Roberge D, Touati N, Maunsell E, Berbiche D. Effects of interdisciplinary teamwork on patient-reported experience of cancer care. BMC Health Services Research. 2017;17:218. (A2)
272. Turner-Stokes L, Williams H, Bill A, Bassett P, Sephton K. Cost-efficiency of specialist inpatient rehabilitation for working-aged adults with complex neurological disabilities: a multicentre cohort analysis of a national clinical data set. BMJ Open. 2016;6:e010238. (A1)
273. Tyrer P, Evans K, Gandhi N, Lamont A, Harrison-Read P, Johnson T. Randomised controlled trial of two models of care for discharged psychiatric patients. BRITISH MEDICAL JOURNAL. 1998;316:106–9. (A1)
274. Uhm J-Y, Kim HS. Impact of the mother-nurse partnership programme on mother and infant outcomes in paediatric cardiac intensive care unit. INTENSIVE AND CRITICAL CARE NURSING. 2019;50:79–87. (A5)
275. Uhm J-Y, Kim HS. Impact of the mother–nurse partnership programme on mother and infant outcomes in paediatric cardiac intensive care unit. Intensive & Critical Care Nursing. 2019;50:79–87. (A1)
276. Valaker I, Fridlund B, Wentzel-Larsen T, Nordrehaug JE, Rotevatn S, Raholm M-B, et al. Continuity of care and its associations with self-reported health, clinical characteristics and follow-up services after percutaneous coronary intervention. BMC HEALTH SERVICES RESEARCH. 2020;20. (A1)
277. Valdes-Stauber J, Kendel U. The differences between referred and non-referred patients to a psychiatric consultation-liaison service in a general hospital. INTERNATIONAL JOURNAL OF PSYCHIATRY IN MEDICINE. (A1)
278. van der Sluis CK, Datema L, Saan I, Stant D, Dijkstra PU. Effects of a nurse practitioner on a multidisciplinary consultation team. JOURNAL OF ADVANCED NURSING. 2009;65:625–33. (A2)
279. van Melle AL, van der Ham AJ, Widdershoven GAM, Voskes Y. Implementation of High and Intensive Care (HIC) in the Netherlands: a Process Evaluation. PSYCHIATRIC QUARTERLY. (A3)
280. van Wilgen CP, Dijkstra PU, Versteegen GJ, Fleuren MJT, Stewart R, van Wijhe M. CHRONIC PAIN AND SEVERE DISUSE SYNDROME: LONG-TERM OUTCOME OF AN INPATIENT MULTIDISCIPLINARY COGNITIVE BEHAVIOURAL PROGRAMME. JOURNAL OF REHABILITATION MEDICINE. 2009;41:122–8. (A4)
281. Verhoef J, Toussaint PJ, Zwetsloot-Schonk JHM, Breedveld FC, Putter H, Vliet Vlieland TPM. Effectiveness of the introduction of an International Classification of Functioning, Disability and Health-based rehabilitation tool in multidisciplinary team care in patients with rheumatoid arthritis. Arthritis Rheum. 2007;57:240–8. (A1)
282. Verkleij M, Beelen A, van Ewijk BE, Geenen R. Multidisciplinary Treatment in Children With Problematic Severe Asthma: A Prospective Evaluation. PEDIATRIC PULMONOLOGY. 2017;52:588–97. (A3)
283. Vidán M, Serra JA, Moreno C, Riquelme G, Ortiz J. Efficacy of a comprehensive geriatric intervention in older patients hospitalized for hip fracture: a randomized, controlled trial. Journal of the American Geriatrics Society. 2005;53:1476–82. (A1)
284. Vluggen TPMM, van Haastregt JCM, Tan FE, Verbunt JA, van Heugten CM, Schols JMGA. Effectiveness of an integrated multidisciplinary geriatric rehabilitation programme for older persons with stroke: a multicentre randomised controlled trial. BMC GERIATRICS. 2021;21. (A1)
285. von Knorring M, Griffiths P, Ball J, Runesdotter S, Lindqvist R. Patient experience of communication consistency amongst staff is related to nurse-physician teamwork in hospitals. NURSING OPEN. 2020;7:613–7. (A1)
286. von Peter S, Ignatyev Y, Johne J, Indefrey S, Kankaya OA, Rehr B, et al. Evaluation of Flexible and Integrative Psychiatric Treatment Models in Germany - A Mixed-Method Patient and Staff-Oriented Exploratory Study. FRONTIERS IN PSYCHIATRY. 2019;9. (A3)
287. Wachter R, Katz P, Showstack J, Bindman A, Goldman L. Reorganizing an academic medical service - Impact on cost, quality, patient satisfaction, and education. JAMA-JOURNAL OF THE AMERICAN MEDICAL ASSOCIATION. 1998;279:1560–5. (A1)
288. Wagner E, Ehrenhofer B, Lackerbauer E, Pawelak U, Siegmeth W. [Rehabilitation of non-specific low back pain. Results of a multidisciplinary in-patient program]. Schmerz. 2007;21:226,-228–33. (A4)
289. Wallen GR, Baker K, Stolar M, Miller-Davis C, Ames N, Yates J, et al. Palliative care outcomes in surgical oncology patients with advanced malignancies: a mixed methods approach. Qual Life Res. 2012;21:405–15. (A1)
290. Walsh TS, Salisbury LG, Merriweather JL, Boyd JA, Griffith DM, Huby G, et al. Increased Hospital-Based Physical Rehabilitation and Information Provision After Intensive Care Unit Discharge: The RECOVER Randomized Clinical Trial. JAMA Internal Medicine. 2015;175:901. (A1)
291. Walton V, Hogden A, Long JC, Johnson JK, Greenfield D. Patients, health professionals, and the health system: influencers on patients’ participation in ward rounds. PATIENT PREFERENCE AND ADHERENCE. 2019;13:1415–29. (A3)
292. Wang M, Wang Y, Meng N, Li X. The factors of patient-reported readiness for hospital discharge in patients with depression: A cross-sectional study. JOURNAL OF PSYCHIATRIC AND MENTAL HEALTH NURSING. (A7)
293. Watterson D, Walter K, O’Brien L, Terrill D, Philip K, Swan I, et al. Trans-disciplinary advanced allied health practitioners for acute hospital inpatients: a feasibility study. INTERNATIONAL JOURNAL FOR QUALITY IN HEALTH CARE. 2019;31:103–9. (A9)
294. Weigl M, Hornung S, Angerer P, Siegrist J, Glaser J. The effects of improving hospital physicians working conditions on patient care: a prospective, controlled intervention study. BMC HEALTH SERVICES RESEARCH. 2013;13. (A1)
295. Williams LJ, Waller K, Chenoweth RP, Ersig AL. Stakeholder perspectives: Communication, care coordination, and transitions in care for children with medical complexity. JOURNAL FOR SPECIALISTS IN PEDIATRIC NURSING. 2021;26. (A3)
296. Williams NH, Roberts JL, Din NU, Charles JM, Totton N, Williams M, et al. Developing a multidisciplinary rehabilitation package following hip fracture and testing in a randomised feasibility study: Fracture in the Elderly Multidisciplinary Rehabilitation (FEMuR). Health Technol Assess. 2017;21:1–528. (A1)
297. Willoch K, Blix HS, Pedersen-Bjergaard AM, Eek AK, Reikvam A. Handling drug-related problems in rehabilitation patients: a randomized study. Int J Clin Pharm. 2012;34:382–8. (A1)
298. Wittink MN, Cross W, Goodman J, Jackson H, Lee HB, Olivares T, et al. Taking the Long View in an Inpatient Medical Unit: A Person-Centered, Integrated Team Approach for Patients With Severe Mental Illnesses. PSYCHIATRIC SERVICES. 2020;71:885–92. (A1)
299. Wittmann M, Spohn S, Schultz K, Pfeifer M, Petro W. [Patient education in COPD during inpatient rehabilitation improves quality of life and morbidity]. Pneumologie. 2007;61:636–42. (A1)
300. Wood HJ, Padilla SB. Peer support work on an inpatient unit for adults experiencing psychosis. PSYCHOSIS-PSYCHOLOGICAL SOCIAL AND INTEGRATIVE APPROACHES. 2019;11:128–37. (A1)
301. Woods JA, Johnson CE, Ngo HT, Katzenellenbogen JM, Murray K, Thompson SC. Symptom-Related Distress among Indigenous Australians in Specialist End-of-Life Care: Findings from the Multi-Jurisdictional Palliative Care Outcomes Collaboration Data. INTERNATIONAL JOURNAL OF ENVIRONMENTAL RESEARCH AND PUBLIC HEALTH. 2020;17. (A3)
302. Woolhouse I, Treml J. The impact of consultant-delivered multidisciplinary inpatient medical care on patient outcomes. Clin Med (Lond). 2013;13:631. (A5)
303. Wynnychuk LA, Otal D, Davidson H, Pyakurel A, Stilos K. Implementation of an educational intervention pilot for residents on acute care general internal medicine wards around the ‘comfort measures strategy’ for end of life care. Prog Palliative Care. 2021;29:20–5. (A3)
304. Yagura H, Miyai I, Suzuki T, Yanagihara T. Patients with Severe Stroke Benefit Most by Interdisciplinary Rehabilitation Team Approach. Cerebrovascular Diseases. 2005;20:258–63. (A1)
305. Yagura H., Miyai I., Suzuki T., Yanagihara T. Patients with severe stroke benefit most by interdisciplinary rehabilitation team approach. Cerebrovasc Dis. 2005;20:258–63. (A6)
306. Zabari A, Lubart E, Ganz FD, Leibovitz A. The effect of a pain management program on the rehabilitation of elderly patients following hip fracture surgery. Isr Med Assoc J. 2012;14:104–6. (A1)
307. Zastrow A, Faude V, Seyboth F, Niehoff D, Herzog W, Lowe B. [Outcome of simultaneous psychosomatic/internal-medicine inpatient care--a naturalistic follow-up study]. Z Psychosom Med Psychother. 2009;55:229–47. (A1)
308. Zatzick D, Roy-Byrne P, Russo J, Rivara F, Droesch R, Wagner A, et al. A randomized effectiveness trial of stepped collaborative care for acutely injured trauma survivors. ARCHIVES OF GENERAL PSYCHIATRY. 2004;61:498–506. (A1)
309. Zatzick D., Russo J., Lord S.P., Varley C., Wang J., Berliner L., et al. Collaborative care intervention targeting violence risk behaviors, substance use, and posttraumatic stress and depressive symptoms in injured adolescents a randomized clinical trial. JAMA Pediatr. 2014;168:532–9. (A1)
310. Zhang C, Zhang L, Huang L, Luo R, Wen J. Clinical pharmacists on medical care of pediatric inpatients: a single-center randomized controlled trial. PLoS One. 2012;7:e30856. (A1)
311. Zobel I, Karim A, Kech S, Berger M, Schramm E. The Adherence of Clinical Management in a Randomized Controlled Trial Results from a Psychotherapy Study. PSYCHOTHERAPIE PSYCHOSOMATIK MEDIZINISCHE PSYCHOLOGIE. 2008;58:395–402. (A1)
312. Zuber P, Tsagkas C, Papadopoulou A, Gaetano L, Huerbin M, Geiter E, et al. Efficacy of inpatient personalized multidisciplinary rehabilitation in multiple sclerosis: behavioural and functional imaging results. JOURNAL OF NEUROLOGY. 2020;267:1744–53. (A1)
313. Zuercher-Huerlimann E, Stewart JA, Egloff N, von Kanel R, Studer M, Holtforth MG. Internal health locus of control as a predictor of pain reduction in multidisciplinary inpatient treatment for chronic pain: a retrospective study. JOURNAL OF PAIN RESEARCH. 2019;12:2095–9. (A3)
314. Zwarenstein M, Goldman J, Reeves S. Interprofessional collaboration: effects of practice-based interventions on professional practice and healthcare outcomes. Cochrane Database Syst Rev. 2009;:CD000072. (A3)
315. Zwarenstein M, Reeves S, Russell A, Kenaszchuk C, Conn LG, Miller K-L, et al. Structuring communication relationships for interprofessional teamwork (SCRIPT): a cluster randomized controlled trial. TRIALS. 2007;8. (A3)
316. Zwarenstein M., Bryant W., Reeves S. In-service interprofessional education improves impatient care and patient satisfaction. J Interprof Care. 2003;17:401–2. (A5)
