# Supplementary material for: Interprofessional collaboration and patient-reported outcomes in inpatient care: a systematic review
Source: Syst Rev. 2022 Aug 13;11:169. doi: 10.1186/s13643-022-02027-x (PMC9375378; doi:10.1186/s13643-022-02027-x)
Supplement: Supplementary file 7 — Additional file 7. Effects satisfaction. [file 13643_2022_2027_MOESM7_ESM.docx]

*Table: Reported adjusted unstandardized mean differences, odds ratios, standardized effect sizes and p-values (between groups) in studies measuring satisfaction*

| **Source (Study type)** | **Study population** | **Measures Satisfaction (total score)** | **Adjusted mean differences**  **(95% CI or SE)** | **Odds ratio (OR) (95% CI)** | **Standardized effect sizes** | **p-value** |
| --- | --- | --- | --- | --- | --- | --- |
| Cheung et al. 2010 [1] (RCT) | Palliative care for patients with preterminal or terminal condition | Unknown measure/ self-developed (.) | . | . | . | 0.56 |
| Counsell et al. 2000 [2] (RCT) | Old age (>70 years old) | Unknown measure/ self-developed (.) | . | . | . | 0.012 |
| Gade et al. 2008 [3] (RCT) | Palliative care | MCOHPQ: Place of care environment scale (0-10) | . | . | . | <0.001 |
|  |  | Doctors, nurses/ other health care providers communication scale (0- 10) | . | . | . | <0.001 |
| O’Leary et al. 2016 [4] (RCT) | General medical patients | Picker Questionnaire (self-adapted): |  |  |  |  |
|  |  | Doctors and nurses do not say different things. (.) | 0.96 (0.47, 1.97) | . | . | 0.91 |
|  |  | Doctors and nurses do not give conflicting information. (.) | 1.84 (1.39, 2.44) | . | . | <0.001 |
|  |  | Doctors and nurses do not talk in front of you as if you were not there. (.) | 1.06 (0.93, 1.21) | . | . | 0.38 |
|  |  | You were involved in decisions about your care. (.) | 0.92 (0.50, 1.71) | . | . | 0.80 |
|  |  | Doctors and nurses worked as a team to care for you. (.) | 1.28 (0.95, 1.73) | . | . | 0.10 |
|  |  | Overall satisfaction (.) | 1.15 (0.77, 1.74) | . | . | 0.50 |
|  |  | Press Ganey: |  |  |  |  |
|  |  | How often did nurses and doctors work as a team to care for you? (.) | 0.84 (0.64, 1.09) | . | . | 0.19 |
|  |  | Staff effort to include you in decisions about your treatment? (.) | 1.01 (0.58, 1.77) | . | . | 0.97 |
|  |  | HCAHPS global ratings: |  |  |  |  |
|  |  | Overall rating of hospital (0-10) | 0.96 (0.73, 1.26) | . | . | 0.75 |
|  |  | Likelihood to recommend (0-10) | 0.73 (0.47, 1.19) | . | . | 0.21 |
| Singer et al. 2019 (RCT) [5] | cancer patients with high distress level (HADS Score $\geq$13) | QPP:  Possibility to converse with doctors and/or psychologists/ social workers (.)  Shared decision-making (.)  Doctors, empathic (.)  Patient orientation (.) | .  .  .  . | 2.3 (1.0, 5.3)  1.3 (0.6, 3.0)  1.1 (0.3, 3.7)  0.6 (0.2, 1.5) | .  .  .  . | 0.05  0.49  0.86  0.27 |
|  | cancer patients with moderate or low distress level (HADS Score <13) | QPP:  Possibility to converse with doctors and/or psychologists/ social workers (.)  Shared decision-making (.)  Doctors, empathic (.)  Patient orientation (.) | .  .  .  . | 0.7 (0.3, 1.8)  0.5 (0.2, 1.2)  0.9 (0.3, 2.8)  0.8 (0.3, 2.3) | .  .  .  . | 0.52  0.12  0.88  0.71 |
| Brédart et al. 2009 [6] (NRS) | cancer | EORTC IN-PATSAT32:  Doctors:  Technical competence (.) | . | . | . | NS |
|  |  | Interpersonal quality (.) | . | . | . | NS |
|  |  | Information (.) | . | . | . | NS |
|  |  | Availability (.) | . | . | . | NS |
|  |  | Nurses/paramedical personnel:  Technical competence (.) | . | . | . | NS |
|  |  | Interpersonal quality (.) | . | . | . | NS |
|  |  | Information (.) | . | . | . | NS |
|  |  | Availability (.) | . | . | . | 0.04 |
|  |  | general satisfaction (.) | . | . | . | NS |
| Marcussen et al. 2020 (NRS) [7] | severe mental illness | CSQ-8 (8-32) | 1.01 (0.6, 1.96) | . | . | 0.04 |

Estimates of adjusted mean differences, standardized effect sizes or p values refer to tests for difference in means between treatment and control groups at the time of follow-up (t1) or to the difference in change scores (t0-t1) between groups.

. = not reported; CI = Confidence interval; CSQ-8 = Client Satisfaction Questionnaire; EORTC IN-PAT32 = EORTC Inpatient Satisfaction with Cancer Care Questionnaire; HCAHPS = Hospital Consumer Assessment of Healthcare Providers and Systems; MCOHPQ = Modified City of Hope Quality of Life Patient Questionnaire; OR = odds ratio; QPP = Quality of Care from the Patient’s Perspective; SE = standard error

References:

1. Cheung W, Aggarwal G, Fugaccia E, Thanakrishnan G, Milliss D, Anderson R, et al. Palliative care teams in the intensive care unit: a randomised, controlled, feasibility study. Crit Care Resusc. 2010;12:28–35.

2. Counsell SR, Holder CM, Liebenauer LL, Palmer RM, Fortinsky RH, Kresevic DM, et al. Effects of a Multicomponent Intervention on Functional Outcomes and Process of Care in Hospitalized Older Patients: A Randomized Controlled Trial of Acute Care for Elders (ACE) in a Community Hospital. Journal of the American Geriatrics Society. 2000;48:1572–81.

3. Gade G, Venohr I, Conner D, McGrady K, Beane J, Richardson RH, et al. Impact of an inpatient palliative care team: a randomized controlled trial. Journal of Palliative Medicine. 2008;11:180–90.

4. O’Leary K.J., Killarney A., Hansen L.O., Jones S., Malladi M., Marks K., et al. Effect of patient-centred bedside rounds on hospitalised patients’ decision control, activation and satisfaction with care. BMJ Qual Saf. 2016;25:921–8.

5. Singer S, Danker H, Meixensberger J, Briest S, Dietz A, Kortmann R-D, et al. Structured multi-disciplinary psychosocial care for cancer patients and the perceived quality of care from the patient perspective: a cluster-randomized trial. J Cancer Res Clin Oncol. 2019;145:2845–54.

6. Brédart A., Dolbeault S., Savignoni A., Simard S., Gomme S., Asselain B., et al. Pilot evaluation of a French interdisciplinary supportive care department. Supportive Care Cancer. 2009;17:1507–16.

7. Marcussen M, Norgaard B, Borgnakke K, Arnfred S. Improved patient-reported outcomes after interprofessional training in mental health: a nonrandomized intervention study. BMC PSYCHIATRY. 2020;20.
