# Supplementary material for: Interprofessional collaboration and patient-reported outcomes in inpatient care: a systematic review
Source: Syst Rev. 2022 Aug 13;11:169. doi: 10.1186/s13643-022-02027-x (PMC9375378; doi:10.1186/s13643-022-02027-x)
Supplement: Supplementary file 8 — Additional file 8. Effects functional ability. [file 13643_2022_2027_MOESM8_ESM.docx]

*Table: Reported adjusted unstandardized mean differences, standardized effect sizes and p-values (between groups) in studies measuring functional ability*

| **Source (Study type)** | **Study population** | **Measures Functional ability (total score)** | **Adjusted mean differences**  **(95% CI or SE)** | **Standardized effect sizes** | **p-value** |
| --- | --- | --- | --- | --- | --- |
| Counsell et al. 2000 [1] (RCT) | Old age (>70 years old) | ADL decline (yes/no) | . | . | 0.11 |
|  |  | Instrumental ADL (number of independent ADL) | . | . | 0.39 |
| Goldberg et al. 2013 [2] (RCT) | Cognitive impairment in old age (>65 years old) | London handicap scale (100) ^‡^ | 0.5 (-5.2, 6.2) | . | 0.87 |
| Hamnes et al. 2012 [3] (RCT) | fibromyalgia | Fibromyalgia Impact Questionnaire (0-10) ^‡^ | -2.76 (-7.7, 2.1) | 0.15 (Cohens‘ d) | 0.265 |
| Hechler et al. 2014 [4] (RCT) | Chronic pain (pediatric) | P-PDI (12-60) ^‡^ | . | . | . |
| Mangels et al. 2009 [5] (RCT) | Chronic low back pain | PDI (.)^‡^ | . | . | . |
|  |  | PSEQ (.) | . | . | . |
|  |  | SES: affective pain perception (.)^‡^ | . | . | . |
|  |  | sensory pain perception (.)^‡^ | . | . | . |
| Monticone et al. 2015 [6] (RCT) | Parkinson’s disease | MDS-UPDRS: Part III (0-132) ^‡^ | -24.5 (3.2) | . | . |
| Wu et al. 2019 [7] (RCT) | critical care survivors | Lawton’s Instrumental Activities of Daily Living Scale (8-30) | . | . | . |
| Semrau et al. 2015 [8] (NRS) | Chronic low back pain | FFbH-R (percentage points) | 0.91 (-1.43, 3.24) | 0.03 (Cohens’ d) | 0.447 |
|  |  | FFkA: sport activity (hours/week) | 0.63 (0.12, 1.13) | 0.106 (Cohens’ d) | 0.015 |

Estimates of adjusted mean differences, standardized effect sizes or p values refer to tests for difference in means between treatment and control groups at the time of follow-up (t1) or to the difference in change scores (t0-t1) between groups.

. = not reported; * = unadjusted; ^‡^ inverted scale (lower score indicate greater impact); FFbH-R = Hannover Functional Ability Questionnaire -back pain; FFkA = Freiburg Questionnaire of physical activity; MDS-UPDRS = Italien Movement Disorder Society Unified Parkinson's Disease Rating Scale; P-PDI = pediatric Pain-Related Disability Index; PDI = Pain Disability Index; PSEQ = Pain Self-efficacy Questionnaire; SES = Pain Perception Scale

References:

1. Counsell SR, Holder CM, Liebenauer LL, Palmer RM, Fortinsky RH, Kresevic DM, et al. Effects of a Multicomponent Intervention on Functional Outcomes and Process of Care in Hospitalized Older Patients: A Randomized Controlled Trial of Acute Care for Elders (ACE) in a Community Hospital. Journal of the American Geriatrics Society. 2000;48:1572–81.

2. Goldberg SE, Bradshaw LE, Kearney FC, Russell C, Whittamore KH, Foster PER, et al. Care in specialist medical and mental health unit compared with standard care for older people with cognitive impairment admitted to general hospital: randomised controlled trial (NIHR TEAM trial). BMJ. 2013;347:f4132.

3. Hamnes B, Mowinckel P, Kjeken I, Hagen KB. Effects of a one week multidisciplinary inpatient self-management programme for patients with fibromyalgia: a randomised controlled trial. BMC MUSCULOSKELETAL DISORDERS. 2012;13.

4. Hechler T, Ruhe A-K, Schmidt P, Hirsch J, Wager J, Dobe M, et al. Inpatient-based intensive interdisciplinary pain treatment for highly impaired children with severe chronic pain: Randomized controlled trial of efficacy and economic effects. PAIN. 2014;155:118–28.

5. Mangels M, Schwarz S, Worringen U, Holme M, Rief W. Evaluation of a behavioral-medical inpatient rehabilitation treatment including booster sessions: a randomized controlled study. Clinical Journal of Pain. 2009;25:356–64.

6. Monticone M, Ambrosini E, Laurini A, Rocca B, Foti C. In-patient multidisciplinary rehabilitation for Parkinson’s disease: A randomized controlled trial. MOVEMENT DISORDERS. 2015;30:1050–8.

7. Wu J, Vratsistas-Curto A, Shiner CT, Faux SG, Harris I, Poulos CJ. CAN IN-REACH MULTIDISCIPLINARY REHABILITATION IN THE ACUTE WARD IMPROVE OUTCOMES FOR CRITICAL CARE SURVIVORS? A PILOT RANDOMIZED CONTROLLED TRIAL. JOURNAL OF REHABILITATION MEDICINE. 2019;51:598–606.

8. Semrau J., Hentschke C., Buchmann J., Meng K., Vogel H., Faller H., et al. Long-term effects of interprofessional biopsychosocial rehabilitation for adults with chronic non-specific low Back pain: A multicentre, quasi-experimental study. PLoS ONE. 2015;10. doi:10.1371/journal.pone.0118609.
