# Supplementary material for: Interprofessional collaboration and patient-reported outcomes in inpatient care: a systematic review
Source: Syst Rev. 2022 Aug 13;11:169. doi: 10.1186/s13643-022-02027-x (PMC9375378; doi:10.1186/s13643-022-02027-x)
Supplement: Supplementary file 9 — Additional file 9. Effects pain. [file 13643_2022_2027_MOESM9_ESM.docx]

*Table: Reported adjusted unstandardized mean differences, standardized effect sizes and p-values (between groups) in studies measuring pain*

| **Source (Study type)** | **Study population** | **Measures Pain (total score)** | **Adjusted mean differences**  **(95% CI or SE)** | **Standardized effect sizes** | **p-value** |
| --- | --- | --- | --- | --- | --- |
| Hechler et al. 2014 [1] (RCT) | Chronic pain (pediatric) | Faces Pain Scale-Revised (0-10) ^‡^ | . | . | . |
| Sidebottom et al. 2015 [2] (RCT) | patients with acute heart failure in palliative care | ESAS (0-90) ^‡^ | 3.69 (3.39, 3.99) | . | 0.000 |
| Angst et al. 2009 [3] (NRS) | Chronic pain | WHYMPI: pain severity (0-100) | . | 0.09 (Hedges‘ g) | 0.034 |
|  |  | life control (0-100) | . | 0.18 (Hedges’ g) | 0.559 |
| Semrau et al. 2015 [4] (NRS) | Chronic low back pain | German Pain Questionnaire (3-33) ^‡^ | -0.05 (-0.34, 0.25) | -0.013 (Cohens’ d) | 0.755 |

Estimates of adjusted mean differences, standardized effect sizes or p values refer to tests for difference in means between treatment and control groups at the time of follow-up (t1) or to the difference in change scores (t0-t1) between groups.

. = not reported; ^‡^ inverted scale (lower score indicate greater impact); ESAS = Edmonton System Assessment Scale; WHYMPI = West Haven-Yale Multidimensional Pain Inventory

References:

1. Hechler T, Ruhe A-K, Schmidt P, Hirsch J, Wager J, Dobe M, et al. Inpatient-based intensive interdisciplinary pain treatment for highly impaired children with severe chronic pain: Randomized controlled trial of efficacy and economic effects. PAIN. 2014;155:118–28.

2. Sidebottom AC, Jorgenson A, Richards H, Kirven J, Sillah A. Inpatient palliative care for patients with acute heart failure: outcomes from a randomized trial. J Palliat Med. 2015;18:134–42.

3. Angst F, Verra ML, Lehmann S, Brioschi R, Aeschlimann A. Clinical effectiveness of an interdisciplinary pain management programme compared with standard inpatient rehabilitation in chronic pain: a naturalistic, prospective controlled cohort study. J Rehabil Med. 2009;41:569–75.

4. Semrau J., Hentschke C., Buchmann J., Meng K., Vogel H., Faller H., et al. Long-term effects of interprofessional biopsychosocial rehabilitation for adults with chronic non-specific low Back pain: A multicentre, quasi-experimental study. PLoS ONE. 2015;10. doi:10.1371/journal.pone.0118609.
