# Supplementary material for: Interprofessional collaboration and patient-reported outcomes in inpatient care: a systematic review
Source: Syst Rev. 2022 Aug 13;11:169. doi: 10.1186/s13643-022-02027-x (PMC9375378; doi:10.1186/s13643-022-02027-x)
Supplement: Supplementary file 10 — Additional file 10. Effects psychiatric morbidity. [file 13643_2022_2027_MOESM10_ESM.docx]

*Table: Reported adjusted unstandardized mean differences, standardized effect sizes and p-values (between groups) in studies measuring psychiatric morbidity*

| **Source (Study type)** | **Study population** | **Measures Psychiatric morbidity (total score)** | **Adjusted mean differences**  **(95% CI or SE)** | **Standardized effect sizes** | **p-value** |
| --- | --- | --- | --- | --- | --- |
| Grudzen et al. 2016 [1] (RCT) | Patients with advanced cancer in palliative care | PHQ-9 (.) | . | . | 0.97 |
| Hamnes et al. 2012 [2] (RCT) | fibromyalgia | GHQ-20 (0-60) ^‡^ | 0.96 (-2.2, 4.1) | 0.10 (Cohens‘ d) | 0.552 |
| Hechler et al. 2014 [3] (RCT) | Chronic pain (pediatric) | DIKJ (0-100) ^‡^ | . | . | . |
| Mangels et al. 2009 [4] (RCT) | Chronic low back pain | BDI (0-63) ^‡^ | . | . | <0.01 |
| Sidebottom et al. 2015 [5] (RCT) | Patients with acute heart failure in palliative care | PHQ-9 (0-27) | 1.42 (1.12, 1.73) | . | 0.000 |
| Wu et al. 2019 [6] (RCT) | Critical care survivors | DASS-21 (.)^‡^ | . | . | . |
| Ziser et al. 2021 [7] (RCT) | Patients with anorexia nervosa | EDE-Q (.)^‡^ | . | . | . |
| Angst et al. 2009 [8] (NRS) | Chronic pain | HADS: depression (0-100) | . | 0.10 (Hedges‘ g) | 0.730 |
|  |  | anxiety (0-100) | . | -0.01 (Hedges‘ g) | 0.229 |
| Hampel et al. 2015 [9] (NRS) | Chronic low back pain and depressive symptoms | ADS (0-3) ^‡^  HADS: anxiety (0-3) ^‡^  SCL-90-R: somatization (0-4)^‡^ | .  .  . | .  .  . | .  .  . |
| Marcussen et al. 2020 [10] (NRS) | Severe mental illness | K10 (10-50)^‡^ | -0.33 (-1.9, 1.2) | . | 0.7 |

Estimates of adjusted mean differences, standardized effect sizes or p values refer to tests for difference in means between treatment and control groups at the time of follow-up (t1) or to the difference in change scores (t0-t1) between groups.

. = not reported; ^‡^ inverted scale (lower score indicate greater impact); ADS = General Depression Scale (Allgemeine Depressions-Skala); BDI = Beck Depression Inventory; DASS-21 = Depression Anxiety Stress Scale; DIKJ = Depression Inventory for Children and Adolescents; EDE-Q = Eating Disorder Examination Questionnaire; GHQ-20 = General Health Questionnaire; HADS = Hospital Anxiety and Depression Scale; K10 = Kessler Psychological Distress Scale; PHQ-9 = Patient Health Questionnaire; SCL-90-R = Symptom Checklist-90-R

References:

1. Grudzen CR, Richardson LD, Johnson PN, Hu M, Wang B, Ortiz JM, et al. Emergency Department–Initiated Palliative Care in Advanced Cancer: A Randomized Clinical Trial. JAMA Oncology. 2016;2:591.

2. Hamnes B, Mowinckel P, Kjeken I, Hagen KB. Effects of a one week multidisciplinary inpatient self-management programme for patients with fibromyalgia: a randomised controlled trial. BMC MUSCULOSKELETAL DISORDERS. 2012;13.

3. Hechler T, Ruhe A-K, Schmidt P, Hirsch J, Wager J, Dobe M, et al. Inpatient-based intensive interdisciplinary pain treatment for highly impaired children with severe chronic pain: Randomized controlled trial of efficacy and economic effects. PAIN. 2014;155:118–28.

4. Mangels M, Schwarz S, Worringen U, Holme M, Rief W. Evaluation of a behavioral-medical inpatient rehabilitation treatment including booster sessions: a randomized controlled study. Clinical Journal of Pain. 2009;25:356–64.

5. Sidebottom AC, Jorgenson A, Richards H, Kirven J, Sillah A. Inpatient palliative care for patients with acute heart failure: outcomes from a randomized trial. J Palliat Med. 2015;18:134–42.

6. Wu J, Vratsistas-Curto A, Shiner CT, Faux SG, Harris I, Poulos CJ. CAN IN-REACH MULTIDISCIPLINARY REHABILITATION IN THE ACUTE WARD IMPROVE OUTCOMES FOR CRITICAL CARE SURVIVORS? A PILOT RANDOMIZED CONTROLLED TRIAL. JOURNAL OF REHABILITATION MEDICINE. 2019;51:598–606.

7. Ziser K, Rheindorf N, Keifenheim K, Becker S, Resmark G, Giel KE, et al. Motivation-Enhancing Psychotherapy for Inpatients With Anorexia Nervosa (MANNA): A Randomized Controlled Pilot Study. FRONTIERS IN PSYCHIATRY. 2021;12.

8. Angst F, Verra ML, Lehmann S, Brioschi R, Aeschlimann A. Clinical effectiveness of an interdisciplinary pain management programme compared with standard inpatient rehabilitation in chronic pain: a naturalistic, prospective controlled cohort study. J Rehabil Med. 2009;41:569–75.

9. Hampel P, Tlach L. Cognitive-behavioral management training of depressive symptoms among inpatient orthopedic patients with chronic low back pain and depressive symptoms: A 2-year longitudinal study. JOURNAL OF BACK AND MUSCULOSKELETAL REHABILITATION. 2015;28:49–60.

10. Marcussen M, Norgaard B, Borgnakke K, Arnfred S. Improved patient-reported outcomes after interprofessional training in mental health: a nonrandomized intervention study. BMC PSYCHIATRY. 2020;20.
