# Supplementary material for: Interprofessional collaboration and patient-reported outcomes in inpatient care: a systematic review
Source: Syst Rev. 2022 Aug 13;11:169. doi: 10.1186/s13643-022-02027-x (PMC9375378; doi:10.1186/s13643-022-02027-x)
Supplement: Supplementary file 11 — Additional file 11. Effects management of own healthcare. [file 13643_2022_2027_MOESM11_ESM.docx]

*Table: Reported adjusted unstandardized mean differences, standardized effect sizes and p-values (between groups) in studies measuring management of one' s own health care*

| **Source (Study type)** | **Study population** | **Measures Management of one’s own health care (total score)** | **Adjusted mean differences**  **(95% CI or SE)** | **Standardized effect sizes** | **p-value** |
| --- | --- | --- | --- | --- | --- |
| Hamnes et al. 2012 [1] (RCT) | Patients with fibromyalgia | EC-17 | 4.26 (0.8, 7.7) | 0.24 (Cohens‘ d) | . |

Estimates of adjusted mean differences, standardized effect sizes or p values refer to tests for difference in means between treatment and control groups at the time of follow-up (t1) or to the difference in change scores (t0-t1) between groups.

. = not reported; EC-17 = Effective Musculoskeletal Consumer Scale

Reference:

1. Hamnes B, Mowinckel P, Kjeken I, Hagen KB. Effects of a one week multidisciplinary inpatient self-management programme for patients with fibromyalgia: a randomised controlled trial. BMC MUSCULOSKELETAL DISORDERS. 2012;13.
