# Supplementary material for: Interprofessional collaboration and patient-reported outcomes in inpatient care: a systematic review
Source: Syst Rev. 2022 Aug 13;11:169. doi: 10.1186/s13643-022-02027-x (PMC9375378; doi:10.1186/s13643-022-02027-x)
Supplement: Supplementary file 12 — Additional file 12. Effects therapeutic relationship. [file 13643_2022_2027_MOESM12_ESM.docx]

*Table: Reported adjusted unstandardized mean differences, standardized effect sizes and p-values (between groups) in studies measuring therapeutic relationship*

| **Source (Study type)** | **Study population** | **Measures Therapeutic relationship (total score)** | **Adjusted mean differences**  **(95% CI or SE)** | **Standardized effect sizes** | **p-value** |
| --- | --- | --- | --- | --- | --- |
| O’Leary et al. 2016 [1] (RCT) | General medical patients | PAM-SF (0-100) | 0.69 (-2.82, 4.19) | . | 0.58 |
| Ziser et al. 2021 [2] (RCT) | Patients with anorexia nervosa | HAQ (.) | . | . | . |

Estimates of adjusted mean differences, standardized effect sizes or p values refer to tests for difference in means between treatment and control groups at the time of follow-up (t1) or to the difference in change scores (t0-t1) between groups.

. = not reported; HAQ= Helping Alliance Questionnaire; PAM-SF = Patient Activation Measure (Short Form)

Reference:

1. O’Leary K.J., Killarney A., Hansen L.O., Jones S., Malladi M., Marks K., et al. Effect of patient-centred bedside rounds on hospitalised patients’ decision control, activation and satisfaction with care. BMJ Qual Saf. 2016;25:921–8.

2. Ziser K, Rheindorf N, Keifenheim K, Becker S, Resmark G, Giel KE, et al. Motivation-Enhancing Psychotherapy for Inpatients With Anorexia Nervosa (MANNA): A Randomized Controlled Pilot Study. FRONTIERS IN PSYCHIATRY. 2021;12.
