# Supplementary material for: Interprofessional collaboration and patient-reported outcomes in inpatient care: a systematic review
Source: Syst Rev. 2022 Aug 13;11:169. doi: 10.1186/s13643-022-02027-x (PMC9375378; doi:10.1186/s13643-022-02027-x)
Supplement: Supplementary file 13 — Additional file 13. Effects treatment success. [file 13643_2022_2027_MOESM13_ESM.docx]

*Table: Reported adjusted unstandardized mean differences, standardized effect sizes and p-values (between groups) in studies measuring treatment success*

| **Source (Study type)** | **Study population** | **Measures Treatment success (total score)** | **Adjusted mean differences**  **(95% CI or SE)** | **Standardized effect sizes** | **p-value** |
| --- | --- | --- | --- | --- | --- |
| Monticone et al. 2015 [1] (RCT) | Parkinson’s disease | GPE (1-5) ^‡^ | . | . | <0.001 |
| Ziser et al. 2021 [2] (RCT) | Anorexia nervosa | URICA-S:  Precontemplation (.)  Contemplation (.)  Action (.)  Maintenance (.) | .  .  .  . | .  .  .  . | .  .  .  . |

Estimates of adjusted mean differences, standardized effect sizes or p values refer to tests for difference in means between treatment and control groups at the time of follow-up (t1) or to the difference in change scores (t0-t1) between groups.

. = not reported; ^‡^ inverted scale (lower score indicate greater impact); GPE = global perceived effect; URICA-S = University of Rhode Island Change Assessment (short version)

Reference:

1. Monticone M, Ambrosini E, Laurini A, Rocca B, Foti C. In-patient multidisciplinary rehabilitation for Parkinson’s disease: A randomized controlled trial. MOVEMENT DISORDERS. 2015;30:1050–8.

2. Ziser K, Rheindorf N, Keifenheim K, Becker S, Resmark G, Giel KE, et al. Motivation-Enhancing Psychotherapy for Inpatients With Anorexia Nervosa (MANNA): A Randomized Controlled Pilot Study. FRONTIERS IN PSYCHIATRY. 2021;12.
